# Economic evaluation of whole genome sequencing for pathogen identification and surveillance – results of case studies in Europe and the Americas 2016 to 2019 

**DOI:** 10.2807/1560-7917.ES.2021.26.9.1900606

**Published:** 2021-03-04

**Authors:** Frank Alleweldt, Şenda Kara, Kris Best, Frank M Aarestrup, Martin Beer, Theo M Bestebroer, Josefina Campos, Gabriele Casadei, Isabel Chinen, Gary Van Domselaar, Catherine Dominguez, Helen E Everett, Ron AM Fouchier, Kathie Grant, Jonathan Green, Dirk Höper, Jonathan Johnston, Marion PG Koopmans, Bas B Oude Munnink, Robert Myers, Celine Nadon, Ami Patel, Anne Pohlmann, Stefano Pongolini, Aleisha Reimer, Shane Thiessen, Claudia Wylezich

**Affiliations:** 1 Civic Consulting, Berlin, Germany; 2 National Food Institute, Technical University of Denmark, Kgs. Lyngby, Denmark; 3 Friedrich-Loeffler-Institut, Greifswald, Germany; 4 Erasmus University Medical Center, Rotterdam, The Netherlands; 5 INEI-ANLIS Dr Carlos G Malbrán, Buenos Aires, Argentina; 6 Istituto Zooprofilattico Sperimentale della Lombardia e dell'Emilia Romagna, Parma, Italy; 7 Public Health Agency of Canada, Winnipeg, Canada; 8 Maryland Department of Health, Baltimore, United States; 9 Animal and Plant Health Agency, Addlestone, United Kingdom; 10 Public Health England, London, United Kingdom; 11 Retired

**Keywords:** Economic evaluation, Whole Genome Sequencing, Next Generation Sequencing, Costs and benefits of pathogen surveillance using WGS, surveillance systems

## Abstract

**Background:**

Whole genome sequencing (WGS) is increasingly used for pathogen identification and surveillance.

**Aim:**

We evaluated costs and benefits of routine WGS through case studies at eight reference laboratories in Europe and the Americas which conduct pathogen surveillance for avian influenza (two laboratories), human influenza (one laboratory) and food-borne pathogens (five laboratories).

**Methods:**

The evaluation focused on the institutional perspective, i.e. the ‘investment case’ for implementing WGS compared with conventional methods, based on costs and benefits during a defined reference period, mostly covering at least part of 2017. A break-even analysis estimated the number of cases of illness (for the example of *Salmonella* surveillance) that would need to be avoided through WGS in order to ‘break even’ on costs.

**Results:**

On a per-sample basis, WGS was between 1.2 and 4.3 times more expensive than routine conventional methods. However, WGS brought major benefits for pathogen identification and surveillance, substantially changing laboratory workflows, analytical processes and outbreaks detection and control. Between 0.2% and 1.1% (on average 0.7%) of reported salmonellosis cases would need to be prevented to break even with respect to the additional costs of WGS.

**Conclusions:**

Even at cost levels documented here, WGS provides a level of additional information that more than balances the additional costs if used effectively. The substantial cost differences for WGS between reference laboratories were due to economies of scale, degree of automation, sequencing technology used and institutional discounts for equipment and consumables, as well as the extent to which sequencers are used at full capacity.

## Introduction

Whole genome sequencing (WGS) is transforming the work of microbiological reference laboratories across the globe. Complete genomic sequences from an isolate or sample have the potential to improve infectious disease surveillance programmes and strengthen epidemiological investigations. Examples include the potential to identify outbreaks earlier through the added value of genome-based cluster detection, the tracking of strains with specific markers relevant for health (for instance antigenicity, virulence, transmissibility, resistance markers) and the monitoring of effectiveness of control measures (for instance vaccination, elimination programmes) [1]. Development of pathogen genomics and the tools, infrastructure and necessary analytics for WGS can be used across sectors (public health, veterinary health or food safety) and pathogen types (viruses, bacteria or parasites), providing potential for further integration of surveillance activities and thus for economies of scale [1,2].

However, in practice, a model currently favoured involves the introduction of WGS into individual pathogen-focused programmes, where the costs of implementing WGS in routine diagnostics and surveillance remain high in comparison to the mainly phenotypic testing currently in use [2]. To better understand the cost differential between conventional methods and WGS in the context of pathogen identification and surveillance, and to identify the main factors affecting the costs and benefits of WGS-based surveillance systems, we conducted an economic evaluation in eight reference laboratories in seven countries (Argentina, Canada, Germany, Italy, the Netherlands, the United States (US) and two institutes from the United Kingdom (UK)). In a second step, we wanted to understand whether the benefits derived from the additional information obtained through the sequencing of pathogens is likely to balance out the additional cost of WGS. For this purpose, we estimated for the example of salmonellosis the number of cases of illness that would need to be prevented each year through the use of WGS in order to ‘break even’ on costs, i.e. in order to make the use of WGS cost-neutral.

## Methods

### Criteria for case study selection

Eight reference laboratories that have started to use WGS on a routine basis for pathogen identification and surveillance were selected for case studies. Five of these institutions – Istituto Zooprofilattico Sperimentale della Lombardia e dell'Emilia-Romagna (IZSLER, Italy), Administración Nacional de Laboratorios e Institutos de Salud (INEI-ANLIS, Argentina), Maryland Department of Health (MDH, US), Public Health Agency Canada (PHAC, Canada), and Public Health England (PHE, UK) – use WGS for characterisation of bacterial isolates in food-borne pathogen surveillance (mostly *Salmonella*, *Listeria*, *Escherichia coli* and *Shigella*). Two reference laboratories use WGS to support avian influenza outbreak investigations, the Animal and Plant Health Agency (APHA, UK) and Friedrich-Loeffler-Institut (FLI, Germany). The last case study concerned the introduction of WGS on clinical samples to direct selection of human influenza virus strains for further characterisation through a culture-based routine at Erasmus Medical Centre (EMC, the Netherlands). The institutions were selected to ensure broad coverage of diverse surveillance contexts and applications, including sector of application (food safety, animal health and public health), coverage of viral (influenza) and bacterial (food-borne) pathogens, routine surveillance and outbreak contexts, as well as the use of different sequencing technologies.

### Study perspective

The economic evaluation of costs and benefits on the basis of the case studies focused on the institutional perspective, i.e. the ‘investment case’ for implementing WGS from the perspective of the reference laboratories. The costs considered therefore included equipment, consumables, staff and other costs that were directly accrued by each of the eight institutions. We assessed the benefits primarily from the perspective of the reference laboratories, focusing on the effects of using WGS on sampling and sampling strategies, analytical results and processes, research and methods applied, and outbreak identification and response, as experienced by each institution. Although the focus was on the costs and benefits accruing to the reference laboratories, we followed the recommendation of the World Health Organization (WHO) to adopt a broader societal perspective where possible [3] and also considered potential effects of the intervention for society (e.g. reduction of disease burden).

### Comparators

For each of the eight reference laboratories, the economic evaluation compared the costs of using WGS to a counterfactual of processing the same number of samples during the specified reference period with the next-best conventional methods for pathogen identification and characterisation. The next-best conventional methods were defined by each individual reference laboratory, taking into account their own standard practice before the implementation of WGS. These next-best conventional methods varied considerably by institution (as specified in the Results section and – in more detail – in Supplementary Table S3). The focus of the analysis was therefore on the measurement and valuation of the marginal (incremental) costs and benefits of using WGS in the surveillance systems subject to this research.

### Time horizon

In line with WHO recommendations for the economic evaluation of surveillance systems, the time horizon of the analysis was limited to a reference period [4]. For the five reference laboratories that conduct surveillance of food-borne pathogens, the reference period was typically 1 year, usually the last 12-month period for which data was available. For the two reference laboratories that conduct surveillance of avian influenza in an outbreak context, the reference period was limited to the duration of the outbreak, which in practice was 3 and 8 months. Case studies covered different reference periods between April 2016 and April 2019, with seven of the eight case studies covering at least a part of the year 2017. The human influenza case study (EMC) covered the influenza season from December 2018 to April 2019. For more details, see case study reports in the Supplement.

### Evaluation of costs

Based on a combination of the relevant WHO guidance and previous studies concerning the evaluation of genomic sequencing technologies [3,5], the costs assessed for each case study were broken down by both analytical step and type of cost. The evaluation of costs focused on the analytical process from receipt and opening of an incoming sample until interpretation and reporting of results by the reference laboratory, both when using WGS and when using conventional methods; the key result of the assessment was the differential cost between both methods on a per-sample basis. We selected four cost categories for the assessment based on the relevant WHO guidance and past studies [3,6]: equipment costs, consumables costs, staff costs and other costs (e.g. for sub-contracting). The assessment of equipment costs was based on the original purchase costs for sequencers and other major laboratory equipment as reported by each institution. It used estimated lifespans for equipment (5 years for computers and 10 years for major laboratory equipment) to calculate annualised costs consistently across case studies. Basic laboratory equipment (e.g. refrigerators or pipettes, but also standard office computers) as well as low-cost equipment of less than EUR 450 were not considered. The assessment included maintenance costs and considered the use rate of equipment (e.g. if a sequencer is used for other purposes as well, this was considered in the calculation). For consumables, the reported purchase costs were adjusted for batch size and for failure rate of analytical processes. Staff costs included wages and social contributions and considered hands-on staff time per sample. Staff time was monetised using country-specific labour costs for professional and technician staff categories (European Union countries) or data on average staff cost provided by the case study institutions (all other countries), plus 25% for overhead costs. For more details concerning the costs assessment methodology, see the Supplement.

### Evaluation of benefits

Based on the results of our desk research and exploratory interviews with experts from reference laboratories and networks already using whole genome sequencing, we identified five areas in which benefits of using WGS for pathogen identification and surveillance are expected to accrue. These are (i) sampling and sampling strategies, (ii) analytical results and processes, (iii) research and methods applied, (iv) outbreak identification and response and (v) effects on wider society. We asked each case study institution to assess in a written questionnaire for each area whether they had concretely experienced a set of specified positive effects of using WGS using a Likert scale. Our contact points in the eight reference laboratories (often the director and/or key staff members) completed the questionnaire and discussed it in depth during the field visits that we conducted to all case study institutions. The benefit assessments of the case study institutions were supplemented by scientific publications and other reports provided by the case study institutions (e.g. related to specific outbreaks they had analysed ex post or in real time using WGS).

### Break-even analysis

The break-even analysis calculated the cost of illness in terms of healthcare utilisation costs, productivity loss and premature death. To estimate the latter, we applied the value of statistical life (VSL) method, which encompasses both material and immaterial losses (i.e. it includes both lost earnings and loss of enjoyment of life). We compared the resulting estimates to the additional cost of using WGS. As the analysis focused only on offsetting the cost of illness and did not take into account additional benefits of using WGS for pathogen identification and surveillance in terms of e.g. effects on research, trade or industry, its results should be understood to be a conservative estimate. The analysis focused on *Salmonella*, as all five case study institutions dealing with food-borne pathogens use WGS to sequence *Salmonella* samples (the other three case study institutions were therefore not considered for this analysis). There is an existing body of work on the costs of salmonellosis infection, making this pathogen the most suitable candidate for the break-even analysis. Our approach closely followed (with some adaptations) the methodology used in the cost-benefit analyses of reducing *Salmonella* in breeding pigs and slaughter pigs, which were conducted for the European Commission in 2010 and 2011 in close coordination with the European Food Safety Authority [7,8]. It also drew on the latest cost-of-illness model developed by the US Department of Agriculture [9]. The detailed approach for the break-even analysis, including a sensitivity analysis in which key assumptions were varied, is presented in the Supplement.

## Results

### Costs

**Figure 1:**
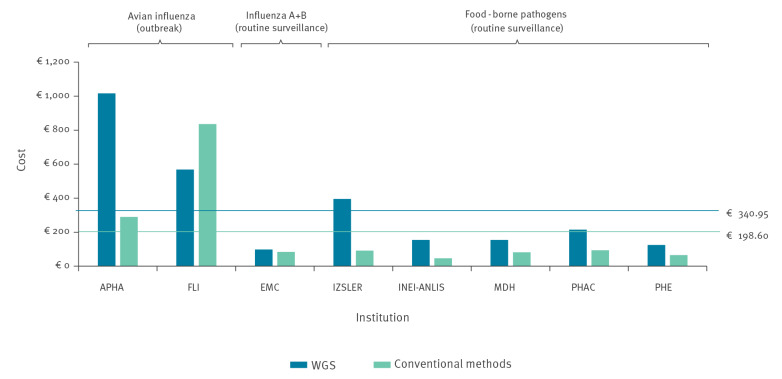
Overall per-sample costs of whole genome sequencing vs conventional methods, case studies covering a specified reference period between 2016 and 2019 (n = 8 institutes)

APHA: Animal and Plant Health Agency; EMC: Erasmus Medical Centre; FLI: Friedrich-Loeffler-Institut; INEI-ANLIS: Instituto Nacional de Enfermedades Infecciosas - Administración Nacional de Laboratorios e Institutos de Salud; IZSLER: Istituto Zooprofilattico Sperimentale della Lombardia e dell'Emilia-Romagna; MDH: Maryland Department of Health; PHAC: Public Health Agency Canada; PHE: Public Health England; WGS: whole genome sequencing.

Overall per-sample costs of WGS exceeded the costs of conventional methods in all reference laboratories analysed except in one (FLI) that had chosen a non-routine method – sequencing of a whole virus genome using Sanger sequencing – as comparator. Excluding this case, the use of WGS was between 1.2 and 4.3 times more expensive than the use of conventional methods, with a cost differential between EUR 15 and EUR 727 per sample (Figure 1).

Blue line: average costs of WGS; green line: average costs of conventional methods. FLI used a non-routine method as comparator (sequencing of a whole genome of a virus using Sanger sequencing). In contrast, APHA used the more limited and less resource-intensive haemagglutinin/neuraminidase analysis, a more typical conventional method for routine analysis.

Table 1 presents the costs of WGS and conventional methods according to cost type. It indicates the additional costs of WGS for each case study, and provides contextual information.

**Table 1:** Overview of per-sample costs of whole genome sequencing vs conventional methods, by cost type, case studies covering a specified reference period between 2016 and 2019 (n = 8 institutes)

Case study area		Avian influenza (HPAI)		Influenza A+B	Food-borne pathogens^a^				
Institution		APHA (UK)	FLI (DE)	EMC (NL)	IZSLER (IT)	INEI-ANLIS (ARG)	MDH (US)	PHAC (CAN)	PHE (UK)
Outbreak or routine surveillance		Outbreak	Outbreak	Routine surveillance	Routine surveillance	Routine surveillance	Routine surveillance	Routine surveillance	Routine surveillance
Number of samples in reference period		26	30	630	175	320	1,767	8,630	15,791
		in 8 months	3 months	5 months	12 months	12 months	12 months	12 months	12 months
WGS									
Sequencer used		Illumina MiSeq	IonTorrent PGM	Nanopore GridION	Illumina MiSeq	Illumina MiSeq	Illumina MiSeq	Illumina MiSeq	Illumina HiSeq
Batch size for sample processing/sequencing		1–2	6	30	24	12	24	32	Processing: 40
									Sequencing: 96
Equipment		€ 58.53	€ 210.71	€ 2.50	€ 163.49	€ 43.02	€ 29.53	€ 75.90	€ 35.23
Consumables		€ 830.97	€ 254.88	€ 33.52	€ 165.37	€ 104.62	€ 104.40	€ 69.75	€ 53.92
Staff costs	*Professionals*	€ 39.63	€ 42.60	€ 15.95	€ 52.35	€ 6.85	€ 20.58	€ 61.82	€ 28.30
	*Technicians*	€ 87.50	€ 60.19	€ 42.83	€ 13.93	€ 0.00	€ 0.00	€ 7.89	€ 7.15
Other costs		€ 0.00	€ 0.00	€ 3.68^b^	€ 0.00	€ 0.00	€ 0.00	€ 0.00	€ 0.00
Total per-sample cost WGS		€ 1,016.63	€ 568.37	€ 98.48	€ 395.14	€ 154.49	€ 154.51	€ 215.36	€ 124.59
Conventional methods^c^									
Method(s) used		Sanger sequencing (HA/NA analysis)	Sanger sequencing (whole genome^d^)	PCR; Sanger sequencing (HA/NA); virus isolation; HI; virus neutralisation; NA STAR	Serotyping; PFGE; PCR; MLVA	Biochemical analysis; serotyping; PCR typing; MaldiTOF; PFGE	PFGE; PCR; MaldiTOF	PFGE; biochemical testing; serotyping	PCR; MLVA; MLST; fAFLP; serotyping; phage typing; PFGE; D-tartrate; glucose gas; AMR; biochemistry
Equipment		€ 78.55	(€ 137.35)^d^	€ 2.66	€ 26.04	n.a.	€ 5.84	€ 12.30	€ 7.11
Consumables		€ 21.91	(€ 360.88)^d^	€ 34.39	€ 20.17	n.a.	€ 32.89	€ 34.95	€ 29.91
Staff costs	*Professionals*	€ 39.63	(€ 230.75)^d^	€ 0.38	€ 3.52	n.a.	€ 42.43	€ 6.72	€ 2.92
	*Technicians*	€ 150.00	(€ 107.00)^d^	€ 45.93	€ 25.88	n.a.	€ 0.00	€ 40.32	€ 23.85
Other costs		€ 0.00	(€ 0.00)^d^	€ 0.00	€ 16.27	n.a.	n.a.	€ 0.00	€ 1.67
Total per-sample cost conventional methods		€ 290.08	(€ 835.98)^d^	€ 83.36	€ 91.87	€ 46.61	€ 81.16	€ 94.29	€ 65.46
Cost difference between WGS and conventional methods									
Additional cost WGS		€ 726.54	(− € 267.61)^d^	€ 15.12	€ 303.27	€ 107.88	€ 73.35	€ 121.07	€ 59.13
Quotient of WGS over conventional methods		3.5	0.7^d^	1.2	4.3	3.3	1.9	2.3	1.9

AMR: antimicrobial resistance; APHA: Animal and Plant Health Agency; ARG: Argentina; CAN: Canada; DE: Germany; EMC: Erasmus Medical Centre; fAFLP: fluorescent amplified fragment length polymorphism; FLI: Friedrich-Loeffler-Institut; HA: haemagglutinin; HI: haemagglutination inhibition; HPAI: highly pathogenic avian *influenza;* INEI-ANLIS: Instituto Nacional de Enfermedades Infecciosas - Administración Nacional de Laboratorios e Institutos de Salud; IT: Italy; IZSLER: Istituto Zooprofilattico Sperimentale della Lombardia e dell'Emilia-Romagna; MDH: Maryland Department of Health; MLST: multilocus sequence typing; MLVA: multilocus variable-number tandem repeat analysis; NA: neuraminidase; n.a.: not available; PFGE: pulsed-field gel electrophoresis; PHAC: Public Health Agency Canada; PHE: Public Health England; UK: United Kingdom; US: United States; WGS: whole genome sequencing.

^a^ *Salmonella* (all), *Listeria* (IZSLER, PHE, PHAC, MDH), *Escherichia coli* and *Shigella* (PHE, INEI-ANLIS, MDH), *Campylobacter* (PHE, MDH), *Vibrio* (MDH).

^b^ Costs for supplementary conventional tests that continue to be part of the WGS workflow (virus isolation, HI, virus neutralisation, NA STAR - on a limited number of samples only).

^c^ Note that the cost of conventional methods is a weighted figure which accounts for the use rate of the various methods across the different pathogens.

^d^ Sequencing of a whole genome of a virus using Sanger sequencing – as indicated by FLI as comparator method – is a resource-intensive process that has generally been replaced by next-generation sequencing; Sanger sequencing would typically be used for the (more limited and less resource-intensive) HA/NA analysis (the comparator method used by APHA). The figures from the FLI case study are therefore placed in brackets and are provided for comparison purposes only.

As Table 1 shows, there was an inverse relationship between sample volume/batch size and total per-sample costs for WGS. The total per-sample costs tend to decrease as the total sample volume increases. The exceptions to this trend are EMC (where efficient equipment use and other factors led to reduced costs), INEI-ANLIS (partly due to lower labour costs in Argentina) and PHAC (where a more extensive bioinformatics infrastructure contributed to higher costs). The two reference laboratories with low sample volume (30 or less) and batch size (6 or less) during (avian influenza) outbreak situations (APHA, FLI) had the highest per-sample costs for WGS, ranging between EUR 568 and EUR 1,017; the reference laboratories that conducted routine surveillance with higher sample volumes/batch sizes had lower per-sample costs for WGS, ranging between EUR 98 to EUR 395. Increasing returns to scale were visible to at least some extent in all major cost types (equipment, consumables and staff time). Other costs were only relevant in a few cases (Table 1) and accrued because of factors such as complementary tests or outsourcing of specific tests.

**Figure 2:**
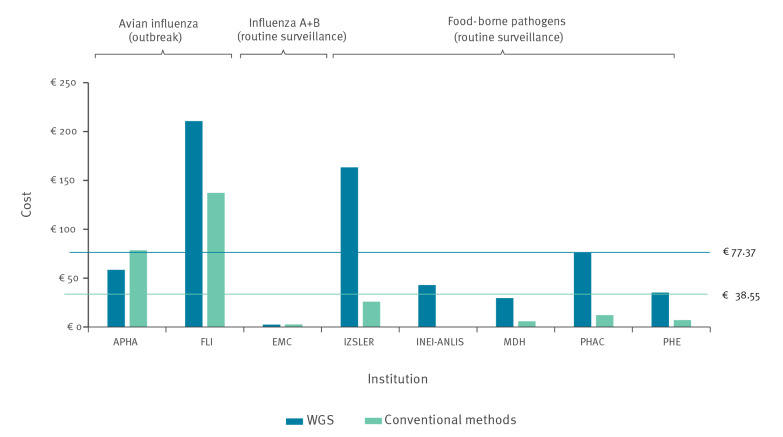
Per-sample equipment costs of whole genome sequencing vs conventional methods, case studies covering a specified reference period between 2016 and 2019 (n = 8 institutes)

APHA: Animal and Plant Health Agency; EMC: Erasmus Medical Centre; FLI: Friedrich-Loeffler-Institut; INEI-ANLIS: Instituto Nacional de Enfermedades Infecciosas - Administración Nacional de Laboratorios e Institutos de Salud; IZSLER: Istituto Zooprofilattico Sperimentale della Lombardia e dell'Emilia-Romagna; MDH: Maryland Department of Health; PHAC: Public Health Agency Canada; PHE: Public Health England; WGS: whole genome sequencing.

The case study institutions varied considerably with respect to the type and amount of equipment used for WGS. This was true not only for the choice of sequencer, but also for the degree of automation in sample processing and library preparation as well as the degree of sophistication in the bioinformatics infrastructure (Supplementary Table S1 describes the type of equipment used by each of the case study institutions). The total purchase cost of equipment for the WGS workflow (not considering basic laboratory equipment) in the year of purchase ranged from a low of ca EUR 75,000 to a high of EUR 3.2 million for several sequencers and a top-of-the-line custom bioinformatics infrastructure. Overall, higher purchase costs tended to reflect higher sample volumes (i.e. multiple sequencers or higher-capacity sequencers) as well as greater investment in automation and/or bioinformatics capacity. As shown in Figure 2, per-sample equipment costs were higher for WGS by a substantial margin in all but two of the case study institutions (APHA and EMC) when compared with the costs of conventional methods.

Blue line: average costs of WGS; green line: average costs of conventional methods. For INEI-ANLIS, a breakdown by cost type was not possible for conventional methods. FLI used a non-routine method as comparator (sequencing of a whole genome of a virus using Sanger sequencing). In contrast, APHA used the more limited and less resource-intensive haemagglutinin/neuraminidase analysis, a more typical conventional method for routine analysis.

This was particularly true for the reference laboratories responsible for food-borne pathogen surveillance which often relied on less costly equipment for conventional methods than the other laboratories and therefore had a greater difference between the equipment costs for WGS and for conventional methods. For the reference laboratories dealing with avian influenza, where the alternative method (Sanger sequencing) requires the use of a sequencer comparable in original purchase price to next-generation sequencers, the difference in costs was relatively smaller (FLI) or even in favour of WGS (APHA). The difference between equipment costs for WGS and conventional methods was negligible for the EMC case study. Note that per-sample equipment costs are greatly influenced by usage rates of the respective equipment, which were considered in this exercise to ensure uniform cost accounting across case studies. For example, the very low per-sample equipment cost at EMC for sequencing (EUR 2.5) was due not only to the comparatively low costs of the sequencer, but also to the fact that is was used efficiently: During the 5 months reference period, only 25% of the sequencer time was allocated to analysing 630 influenza samples (the remaining 75% of sequencer time was used for analyses unrelated to the surveillance task, and therefore not included in the cost estimation).

**Figure 3:**
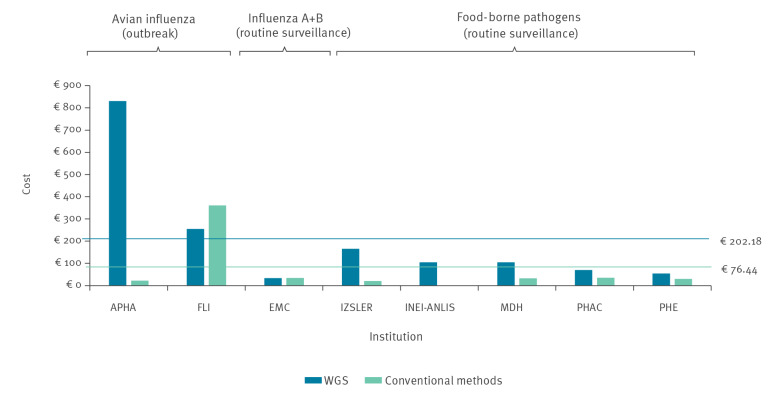
Per-sample consumables costs of whole genome sequencing vs conventional methods, case studies covering a specified reference period between 2016 and 2019 (n = 8 institutes)

APHA: Animal and Plant Health Agency; EMC: Erasmus Medical Centre; FLI: Friedrich-Loeffler-Institut; INEI-ANLIS: Instituto Nacional de Enfermedades Infecciosas - Administración Nacional de Laboratorios e Institutos de Salud; IZSLER: Istituto Zooprofilattico Sperimentale della Lombardia e dell'Emilia-Romagna; MDH: Maryland Department of Health; PHAC: Public Health Agency Canada; PHE: Public Health England; WGS: whole genome sequencing.

Per-sample consumables costs were higher for WGS than for conventional methods in all but two reference laboratories (FLI, which used a non-routine method as comparator, and EMC, where other factors led to lower costs, see discussion below), and sometimes considerably so. For example, in the case of APHA, the consumables cost for WGS (EUR 831) was nearly 38 times the consumables cost for conventional methods (EUR 22), owing to the time sensitivity of obtaining data for the index cases of an avian influenza outbreak. This led to low batch sizes for sequencing, increasing consumables cost per sample. In addition, the consumables used for Sanger sequencing are cheaper. This is illustrated in Figure 3.

Blue line: average costs of WGS; green line: average costs of conventional methods. For INEI-ANLIS, a breakdown by cost type was not possible for conventional methods. FLI used a non-routine method as comparator (sequencing of a whole genome of a virus using Sanger sequencing). In contrast, APHA used the more limited and less resource-intensive haemagglutinin/neuraminidase analysis, a more typical conventional method for routine analysis.

The determining factor was the higher cost of kits and reagents required for WGS, a specific cost driver in this context being the overall throughput in terms of number of samples and the batch size for sequencing. The total per-sample consumables cost decreased as the average throughput and batch size increased. However, the EMC case study with a sample volume of 630 over a 5-month period showed per-sample consumables costs (EUR 34) lower than those of reference laboratories with a much higher throughput of samples for sequencing during the reference period (such as PHE with more than 15,000 samples over a 12-month period, see Discussion).

**Figure 4:**
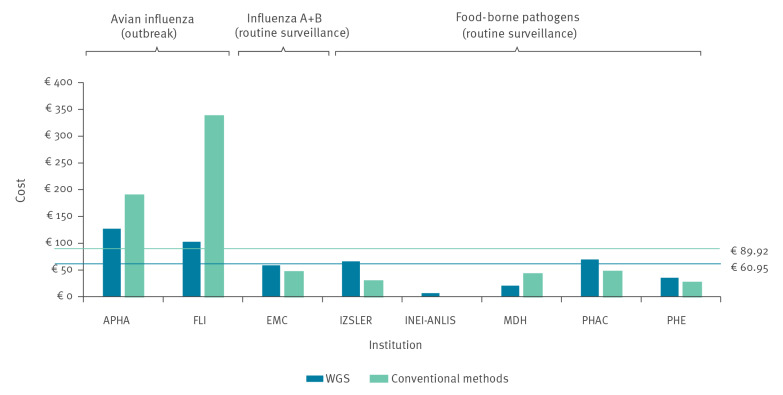
Per-sample staff costs of whole genome sequencing vs conventional methods, case studies covering a specified reference period between 2016 and 2019 (n = 8 institutes)

APHA: Animal and Plant Health Agency; EMC: Erasmus Medical Centre; FLI: Friedrich-Loeffler-Institut; INEI-ANLIS: Instituto Nacional de Enfermedades Infecciosas - Administración Nacional de Laboratorios e Institutos de Salud; IZSLER: Istituto Zooprofilattico Sperimentale della Lombardia e dell'Emilia-Romagna; MDH: Maryland Department of Health; PHAC: Public Health Agency Canada; PHE: Public Health England; WGS: whole genome sequencing.

With respect to staff costs, hands-on staff time estimates per sample differed considerably between case study institutions. Estimates of professional staff time per sample for WGS ranged from 18 to 90 min (with the costs ranging from EUR 7 to EUR 62), while estimates of technician staff time per sample for WGS ranged from 0 to 210 min (with associated costs between EUR 0 and EUR 88). Figure 4 provides an overview of per-sample staff costs.

Blue line: average costs of WGS; green line: average costs of conventional methods. For INEI-ANLIS, breakdown by cost type was not possible for conventional methods. FLI used a non-routine method as comparator (sequencing of a whole genome of a virus using Sanger sequencing). In contrast, APHA used the more limited and less resource-intensive haemagglutinin/neuraminidase analysis, a more typical conventional method for routine analysis.

For four of the case study institutions, staff costs were lower for conventional methods than for WGS. They were, however, much higher for conventional methods used in the two reference laboratories dealing with avian influenza. This was most probably a consequence of the lower batch sizes in an outbreak context, the more complex and time-intensive steps involved in Sanger sequencing (the conventional method used in these case studies) as well as the greater involvement of professional staff. In contrast, the conventional methods used by reference laboratories for food-borne pathogen surveillance tend to be more straightforward (e.g. PCR or MLVA analysis, each requiring less than 10 min of staff time per sample) and rely often entirely on technician staff rather than professional staff.

### Benefits

**Figure 5:**
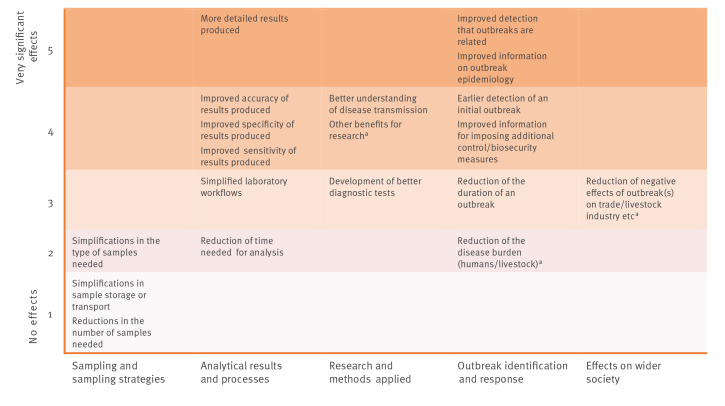
Key positive effects of using whole genome sequencing as experienced by case study institutions (based on average assessments), case studies covering a specified reference period between 2016 and 2019 (n = 8 institutes)

^a^ Only four or fewer case study institutions provided an assessment.

Case study institutions were asked to assess specific positive effects or impacts of using WGS observed during the reference period on a scale from 1 (no effect at all) to 5 (very significant positive effect). All eight reference laboratories reported major benefits of using WGS for pathogen identification and surveillance. Benefits were experienced in different areas, most notably with respect to analytical results/processes and outbreak identification/response, but also related to sampling and sampling strategies, research and methods applied, and effects on wider society (see detailed discussion below). Figure 5 presents these effects and ranks them based on average assessments. Effects shown towards the top of the figure were indicated by the reference laboratories to be more significant.

#### Effects on sampling and sampling strategies

As indicated in Figure 5, most of the reference laboratories did not observe effects on sampling and sampling strategies. One of the reasons was that sampling is mostly not within their purview, as samples are independently collected by external institutional partners and sent to the case study institutions for further analysis. However, one of the reference laboratories active in food-borne pathogen surveillance (IZLER) reported that the introduction of WGS had resulted in changes to how food safety officials conducted sampling, by moving from sampling of products to environmental sampling (e.g. by swabbing surfaces in production sites). While production environment testing was already used to monitor food production sites compare to testing of finished products before WGS, the high precision of WGS allowed to establish an unambiguous link between environmental samples from food producers and clinical samples.

#### Effects on analytical results and processes

Most of the reference laboratories experienced considerable positive effects of using WGS on the quality of the results produced in terms of detail, accuracy, specificity and sensitivity. For example for food-borne pathogens, WGS analysis provided insights into how bacterial strains diversify over time, allowing strains to be identified as linked when under previous methods, they would have been considered unrelated (indicated by PHE). It also allowed for the investigation of resistance gene profiles. In the context of avian influenza identification and surveillance, the use of WGS provided many sequence reads, resulting in higher accuracy and greater statistical confidence in the outputs and rapidly delivering viral genome-spanning information on genotype, pathotype and mutations (reported by APHA).

Positive effects on laboratory processes and resources were reported to be mostly negligible by the reference laboratories dealing with avian and human influenza. In contrast, the reference laboratories that used WGS in the context of routine surveillance of food-borne pathogens emphasised the simplification of laboratory workflows, especially with respect to the reduction of the number of hands-on steps for analysis. PHE elaborated that multiple validated processes for different bacteria had been replaced with WGS, pathogens were being processed in fewer rooms and samples containing live organisms required fewer hands-on interactions. PHE also indicated that replacing conventional methods with WGS made it easier to monitor its own laboratory processes, predict costs and identify ways to reduce costs in the future [10].

For all institutions, the use of WGS also affected the turnaround time, defined as the usual number of days of work from receipt and opening of an incoming sample to the reporting of the results: For the reference laboratories dealing with avian and human influenza, the turnaround time ranged between 2 and 5 days of work for WGS analysis (and as low as 10 h in the EMC case study) compared with 1–2 days for haemagglutinin/neuraminidase (HA/NA) analysis or 8 days for analysis of a whole genome using Sanger sequencing. For the food-borne pathogen case studies, the usual turnaround time was 5–10 days for WGS analysis. The turnaround time for the full analysis of a food-borne pathogen using conventional methods was typically 4–15 days, depending on the pathogen and analysis required.

In general, turnaround time for WGS analysis remained relatively constant. The differential effect of WGS on turnaround time therefore depended on the complexity of the conventional analysis required. The turnaround time for conventional methods increased based on the amount of information required and the corresponding number of different tests (especially consecutive tests) that are needed. Consequently, the turnaround time for WGS tended to be higher than the turnaround time for conventional methods when only basic information about the pathogen was needed, and lower when a more detailed characterisation was required.

#### Effects on research and epidemiological investigations

Multiple case study institutions, including PHE, INEI-ANLIS, MHD and IZSLER, have made use of WGS in order to study past outbreaks and draw new conclusions regarding outbreak epidemiology. For example, PHE research indicated that cluster analysis of *Shigella sonnei* infections using WGS had uncovered novel transmission routes [11]. With respect to the analysis of viruses, the benefits of using WGS included obtaining more detailed genetic information regarding virus strains and how these evolve. As viruses mutate particularly quickly, WGS can be used to identify novel viruses, reassortants, and mixed infections that would be missed by other methods. An example of WGS research applications in a non-outbreak context was a study by one of the case study institutions (IZLER) that focused on environmental sampling for *Listeria* along the production chain for ham using WGS [12]. The analysis indicated at which stages in the production chain and on which types of environmental surfaces contamination was most likely to occur. Clonal contamination patterns were also examined to draw insights on transmission within and between plants, as well as assess the efficacy of hygiene measures through repeated sampling more than six months later.

A minority of the reference laboratories also observed positive effects of WGS on the development of better diagnostic tests, e.g. by evaluating the robustness of real-time PCR assays [13], or using WGS in the development of new PCR assays (as reported by PHE). Other reported benefits for research mostly related to the large amount of sequence data available through WGS, which can be explored for further research. WGS makes it easier for reference laboratories to collaborate internationally, as genome sequences can be sent more quickly and easily than physical samples, and stored genomic data can be mined again as new genes or other genetic elements become relevant.

#### Effects on outbreak detection and response

All laboratories experienced clear positive effects of using WGS in terms of improved detection that outbreaks are related and improved information on outbreak epidemiology. Using WGS data affected the number and size of clusters detected. Clusters already identified with conventional methods were confirmed or split with the help of sequence data, and a larger number of smaller outbreaks was identified. In a 2018 publication aiming to quantify the operational burden associated with the use of WGS for cluster analysis of two *Salmonella* serovars, PHE determined that during a 1-year period between 2014 and 2015, WGS had identified a notably larger number of clusters caused by *Salmonella* Enteritidis or *Salmonella* Typhimurium than conventional methods [14]. PHAC reported that the number of outbreaks caused by *Salmonella* Enteritidis detected increased substantially from less than 20 each year between 2012 and 2016 to more than 100 in 2017, the first year with routine use of WGS. PHAC also reported, however, that the number of *Listeria* outbreaks detected had actually decreased in the first year of WGS implementation, as PFGE had previously been detecting outbreaks that did not exist and had led to an inefficient use of resources investigating non-existent outbreaks.

The practical benefits of using WGS in an outbreak context were also documented with respect to specific outbreaks. For example, the retrospective analysis of a 2013 outbreak of salmonellosis in Italy by IZLER provided evidence that the use of WGS would have allowed for human cases to be linked to the source of the outbreak 2 months before the source had been identified using PFGE and MLVA [15]. A specific benefit of WGS was reported with respect to so-called ‘slow-burn’ outbreaks with low case numbers but continuous transmission over a long period of time which are often not identified with traditional methods. The use of WGS allowed PHAC to identify 17 separate outbreaks of *Salmonella* Enteritidis infections associated with the same food (raw frozen breaded chicken products), which had not been picked up with conventional methods. This allowed for stricter, Canada-wide regulations to be adopted for this product category, which was estimated by PHAC to be responsible for up to 40% of the disease burden attributable to *Salmonella* Enteritidis [16]. Similarly, an outbreak of *Salmonella* Enteritidis infections in reptile feeder mice in the UK might not have been detected at all without WGS because of the low case numbers [17]. The outbreak was detected in 2015 following the implementation of SNP typing at PHE and had been occurring undetected by traditional surveillance procedures since at least January 2012. The results of the epidemiological investigation initiated on the basis of the WGS data identified the outbreak source (handling of the feeder mice or snakes infected via the mice) and a series of recommendations could be issued to control infections at the farm level and point of sale. Both examples provide clear evidence that the use of WGS has led in practice to a reduction in the disease burden in humans because measures were taken to end the (previously undetected) outbreaks. Whether or not WGS leads to earlier detection of outbreaks depends, however, on the point in time when WGS is used in the analytical process. In a 2016/17 outbreak of highly pathogenic avian *influenza* in Germany (reported by FLI) and a 2016 *Shigella sonnei* outbreak in Buenos Aires (reported by INEI-ANLIS), the use of WGS did not lead to an earlier detection as sequencing was only conducted after the outbreak had been detected through conventional methods. But even in these and similar examples, the use of WGS allowed for better linkage to the sources of the outbreak.

Finally, the outbreak cases analysed (in the context of the avian influenza and food-borne pathogen case studies) confirmed that WGS provided better information for imposing control measures and for assessing their effectiveness. For example, APHA reported that the WGS data enabled them to better assess the public health risk of an avian influenza outbreak by revealing whether particular virus strains included mutations that could pose a risk of transmission to humans (compared with the previous situation when HA/NA analysis with Sanger sequencing was used). The use of WGS also allowed for confirmation of whether cases of avian influenza in domestic poultry occurred through introduction by wild birds or also through secondary infections between farms, indicating gaps in farm biosecurity measures [18,19].

### Break-even analysis

For each of the five reference laboratories that conduct *Salmonella* surveillance, we calculated both the absolute number and the proportion of reported cases of salmonellosis within the geographical jurisdiction of each institution that would need to be avoided to make the use of WGS cost-neutral. The results are presented in Table 2.

**Table 2:** Results of break-even analysis, whole genome sequencing vs conventional methods, case studies covering a specified reference period between 2016 and 2018 (n = 5 institutes)

Case study institution	IZSLER (IT)	INEI-ANLIS (ARG)	MDH (US)	PHAC (CAN)	PHE (UK)	Average
Cost per sample (WGS)	€ 395.14	€ 154.49	€ 154.51	€ 215.36	€ 124.59	€ 208.82
Cost per sample (conventional methods)	€ 91.87	€ 46.61	€ 81.16	€ 94.29	€ 65.46	€ 75.88
Differential cost of WGS compared with conventional methods	€ 303.27	€ 107.88	€ 73.35	€ 121.07	€ 59.13	€ 132.94
Number of samples per year (*Salmonella*)	110	128	1,010	8,273	10,147	3,934
Total additional costs per year due to the use of WGS	€ 33,360	€ 13,809	€ 74,084	€ 1,001,623	€ 599,992	€ 344,573
Average cost per reported case of salmonellosis	€ 12,124	€ 11,821	€ 13,225	€ 12,174	€ 12,401	€ 12,349
Number of reported cases of salmonellosis that need to be avoided to break even	2.8	1.2	5.6	82.3	48.3	28.0
Number of cases of salmonellosis reported annually^a^	276^b^	758	906	7,665	8,770	4,404
Percentage of total number of reported cases of salmonellosis that need to be avoided to break even	1.0%	0.2%	0.6%	1.1%	0.6%	0.7%

ARG: Argentina; CAN: Canada; INEI-ANLIS: Instituto Nacional de Enfermedades Infecciosas - Administración Nacional de Laboratorios e Institutos de Salud; IT: Italy; IZSLER: Istituto Zooprofilattico Sperimentale della Lombardia e dell'Emilia-Romagna; MDH: Maryland Department of Health; PHAC: Public Health Agency Canada; PHE: Public Health England; UK: United Kingdom; US: United States; WGS: whole genome sequencing.

^a^ Data on cases of salmonellosis refer to the geographical jurisdictions of the institution (see case study reports in the Supplement). Where a case study institution processed samples originating from the whole country (Canada, Argentina), data on salmonellosis refer to the country as a whole. Where a case study institution only processed samples from a specific geographical region within a country, data on salmonellosis refer to this particular region (England, Wales and Northern Ireland in the UK, Emilia-Romagna in Italy, and Maryland in the US).

^b^ Regional data approximated as a population-based proportion of national data, as no regional data was available.

'Average' column presents averages of the case study figures in each respective row.

Own calculation, see Supplement.

As indicated in Table 2, the number of cases of salmonellosis that would need to be avoided annually to break even on costs ranged from one case within INEI-ANLIS’ area of jurisdiction (Argentina) to a maximum of 82 cases within PHAC’s area of jurisdiction (Canada). While the absolute numbers differed considerably between the jurisdictions, the proportion of reported cases that need to be avoided to break even was comparable, ranging from 0.2% to 1.1% (with an average of 0.7%). This figure refers to the proportion of reported cases and not to the proportion of total cases in the community. Salmonellosis cases not recorded in national surveillance statistics were excluded from this analysis. It is also notable that because of the high costs associated with premature death, the number of deaths that would need to be avoided to break even on WGS lay well below 1 for all five reference laboratories that were involved in *Salmonella* surveillance, indicating that if even a single death from salmonellosis were avoided each year through WGS, it would more than break even on costs. For comparison, about 50 deaths from salmonellosis are reported per year in PHE’s jurisdiction of England, Wales and Northern Ireland [20], meaning that one death avoided annually would comprise 2% of all registered salmonellosis deaths. In fact, given the high cost attached to premature death, avoiding one salmonellosis death every 7 years in PHE’s jurisdiction would be sufficient to more than break even on costs; for the other four case studies for which the break-even analysis was conducted, the number of years was even higher .

We conducted a sensitivity analysis in which key assumptions of the analysis were tested. The largest single cost component in the average cost per case of salmonellosis was premature death, which even at a low rate of occurrence overshadowed costs of healthcare or productivity loss (comprising ca 95% of the total average cost of an infection). In order to test this assumption, we recalculated the break-even analysis using lower and higher bound estimates for the costs of premature death. We also tested variations in the likelihood of premature death. Under all sensitivity scenarios, the proportions of reported cases of salmonellosis that would need to be avoided in order to break even on the costs of WGS were still lower than 2.5% in all case study jurisdictions. For more details, see Supplement.

## Discussion

This analysis of the practical experiences of eight reference laboratories in Europe and the Americas between 2016 and 2019 confirmed that WGS had higher per-sample costs on average than conventional laboratory methods: WGS was between 1.2 and 4.3 times more expensive than routine conventional methods. Several factors affected the costs of WGS. There was a general tendency of increasing returns to scale with WGS analysis, with average per-sample costs of WGS tending to decrease as sample volume and batch size increase. Reference laboratories which deal with a high volume of samples are therefore likely to achieve a lower per-sample cost than smaller institutions processing fewer samples. However, centralised analysis may come with a trade-off in terms of turnaround time because of increased time for shipping of samples. In countries with a decentralised system, regional laboratories often have an important role, which limits the volume of samples per laboratory. Time pressure in an outbreak context often does not allow for batching of samples. Therefore cost of using WGS for avian influenza outbreak surveillance is high – the average cost of the two reference laboratories in our study was EUR 793 per sample. In contrast, the average cost for the five reference laboratories that used WGS for routine surveillance of food-borne pathogens was much lower at EUR 209 per sample. Interestingly, in the case of human influenza surveillance using a WGS-based workflow (EMC), the cost was the lowest of all institutions analysed at EUR 98 per sample (only slightly higher than the cost for the conventional workflow). 

The costs of the sequencing platform used (producer, model and related consumables) may differ considerably and are further influenced by the extent to which sequencers and other equipment are used at full capacity or not. The EMC case study provides evidence that a lower cost sequencer and an efficient set up may be capable of limiting costs with smaller batch sizes and sample volumes. The sequencer was used both for sequencing of surveillance samples and for other purposes, thereby reducing the per-sample equipment costs of surveillance. In contrast, other reference laboratories did not or could not use equipment at full capacity for a variety of reasons, including time constraints (outbreak context), overall sample volume and the degree to which the purchased equipment considered future (potentially higher) sample volumes. In addition, EMC was able to obtain discounts of up to 60% compared with the list price for WGS equipment and key consumables by joining together with other university hospitals and collectively negotiating with the suppliers.

The reference laboratories reported that lack of competition among suppliers of sequencing equipment and dependency on specific consumables for sequencing was a key factor driving costs and made WGS currently less affordable. Other cost factors were the level of automation (affecting staff time demands) and the costs of the bioinformatics infrastructure, which differed greatly between reference laboratories. Especially for smaller or regional laboratories, cloud-based applications for the bioinformatics analysis may lead to considerable cost savings. This was the case for MDH, which used online tools for sequencing analysis (www.genomicepidemiology.org). It is expected that future cost reductions in automation and sequencing (as well as in computing and data storage) will drive down the costs of pathogen surveillance using WGS substantially (and also further reduce turnaround time).

Even at cost levels documented here, WGS provides a level of additional information that more than balances out the additional costs if used effectively. All case study institutions reported major benefits of using WGS for pathogen identification and surveillance, streamlining their work flows, making analytical processes more amenable to automation and improving the ways in which outbreaks are detected and controlled. As the example of food-borne illnesses showed most clearly, WGS may identify more outbreak clusters and can reduce overall cases of illness, if public health systems are equipped and funded adequately enough to take effective measures. Our break-even analysis indicates that in the case of *Salmonella* surveillance, only a modest percentage (0.2–1.1%) of reported salmonellosis cases would need to be avoided each year through the use of WGS in order to make the adoption of the technology cost-neutral from a public health perspective. The consistency of results across the five reference laboratories and the sensitivity analysis support the robustness of the main conclusion of the break-even analysis: That avoiding just one premature death due to salmonellosis over a period of several years through the use of WGS was sufficient to break even on costs from a public health perspective for all of the *Salmonella* surveillance systems analysed. In this context it is notable that the number of deaths due to food-borne infections is likely to be underestimated. Salmonellosis reporting is mostly done at the time of diagnosis and therefore before the final outcome is known. There are indications for substantial excess mortality in patients after salmonellosis up to a year after the infection that cannot be explained by other factors [21].

While the results of the break-even analysis cannot be generalised, they illustrate the potential public health benefits of using WGS. The benefits of using WGS for pathogen identification and surveillance depended largely on the setup and functioning of the surveillance system: the later in the analytical process WGS is used, the more limited the potential benefits may be in an outbreak context (e.g. if samples are only sequenced weeks after detection of an outbreak through other methods). The case studies highlighted the benefits of WGS as part of a One Health approach, especially in the surveillance of food-borne pathogens. Identifying linkages between human cases and sources in the food system through WGS in real time critically depends on a continuous exchange of sequencing data from routine laboratory surveillance of samples from human, animal and food sources.

When interpreting this economic evaluation, it is crucial to note its limitations. The case studies with eight reference laboratories are characterised by different variables: different pathogens, different conventional methods used as comparators, different infrastructures and different types of surveillance systems. The per-sample costs calculated in this study were the actual costs incurred by these eight reference laboratories, reflecting their specific situations. This implies that the cost estimates cannot be used to extrapolate costs to other institutions. The approach of focusing on differential costs with a consistent and uniformly applied methodology simplified the complex analysis, as costs that are clearly unaffected (e.g. for depreciation of laboratory buildings) did not need to be assessed, allowing us to focus in detail on those costs and benefits where changes were caused by the use of WGS. Limitations are also notable for the break-even analysis, which focused on one specific pathogen (*Salmonella*). The results are therefore not applicable to the surveillance of other pathogens.

## Supplementary Data

7926000 Supplement
