# Supplementary material for: Economic evaluation of whole genome sequencing for pathogen identification and surveillance – results of case studies in Europe and the Americas 2016 to 2019
Source: Euro Surveill. 2021 Mar 4;26(9):1900606. doi: 10.2807/1560-7917.ES.2021.26.9.1900606 (PMC7934224; doi:10.2807/1560-7917.ES.2021.26.9.1900606)
Supplement: Supplement [file 19-00606_ALLEWELDT_Supplement.pdf]

Economic evaluation of Whole Genome Sequencing for pathogen identification and surveillance – results of case studies in Europe and the Americas (2016 to 2019)

Supplementary materials

This supplementary material is hosted by Eurosurveillance as supporting information alongside the article *Economic evaluation of Whole Genome Sequencing for pathogen identification and surveillance – results of case studies in Europe and the Americas (2016 to 2019)*, on behalf of the authors, who remain responsible for the accuracy and appropriateness of the content. The same standards for ethics, copyright, attributions and permissions as for the article apply. Supplements are not edited by Eurosurveillance and the journal is not responsible for the maintenance of any links or email addresses provided therein.

Table of contents

1. COSTS 3
2. BENEFITS 6
3. BREAKEVEN ANALYSIS 12
   - 3.1. Cost of illness calculation 15
   - 3.2. Average cost per case of salmonellosis 22
   - 3.3. Results of the breakeven analysis 23
   - 3.4. Sensitivity analysis 26
4. CASE STUDY REPORTS 30
   - 4.1. Animal and Plant Health Agency (APHA) 30
   - 4.2. Friedrich-Loeffler-Institut (FLI) 39
   - 4.3. Erasmus Medical Centre (EMC) 47
   - 4.4. Istituto Zooprofilattico Sperimentale della Lombardia e dell'Emilia-Romagna (IZSLER) 55
   - 4.5. Administración Nacional de Laboratorios e Institutos de Salud (ANLIS) 64
   - 4.6. Maryland Department of Health (MDH) 70
   - 4.7. Public Health Agency Canada (PHAC) 77
   - 4.8. Public Health England (PHE) 85

APPENDIX. COST DATA COLLECTED FROM CASE STUDY INSTITUTIONS 96

1. Costs

Assessment of costs – methodology and additional information

Based on a combination of the relevant WHO guidance as well as previous studies concerning the evaluation of genomic sequencing technologies [1,2], the costs assessed for each case study are broken down by both analytical step and type of cost. The analytical steps that were considered within the scope of the economic evaluation for WGS are *sample preparation and sequencing* and *bioinformatics and other analyses.* Costs related to *outbreak response* were not considered, as data on costs and benefits of response activities are often difficult to obtain ex-post, and measurement problems are substantial. In addition, there were differences in the response mandate across case study institutions (e.g. while some are involved in determining response measures, others are not). Therefore, while we have recorded the benefits of WGS for outbreak response as concretely experienced by the case study institutions, the evaluation of costs focused *on the analytical process from receipt and opening of an incoming sample until interpretation and reporting of results by the reference laboratory*, both when using WGS and when using conventional methods, with the key result of the assessment being *the differential cost between both methods on a per-sample basis.* Four cost categories were selected for the assessment based on the relevant WHO guidance and past studies by the authors [3,4]: equipment costs, consumables costs, staff costs and other costs (e.g. for sub-contracting). The assessment of *equipment costs* is based on the original purchase costs for sequencers and other major laboratory equipment as reported by each institution. It uses estimated lifespans for equipment (5 years for computers and 10 years for major laboratory equipment) to calculate annualised costs consistently across case studies. Basic laboratory equipment (e.g. refrigerators or pipettes, but also standard office computers and software such as Word and Excel) as well as low-cost equipment of less than EUR 450 were not included. The assessment includes maintenance costs and considers the use rate of equipment (e.g. if a sequencer in a case study institution was used only 70% of the time for the analysis of the samples considered in the case study, and 30% for other purposes, the annualised costs of the sequencer were reduced accordingly). For *consumables*, the reported purchase costs were adjusted for batch size and for the failure rate of analytical processes. *Staff costs* include wages and social contributions and consider hands-on staff time per sample, i.e. the amount of staff time used for an activity, and not the duration of the activity: unsupervised processes (such as incubation periods or sequencing runs) are not included in the estimates. Hands-on staff time was monetised using country-specific labour costs for professional and technician staff categories (using EUROSTAT data, for EU countries), or average staff cost data provided by the case study institutions (Argentina, Canada, US), plus 25% for overhead costs. Cost data was collected from each case study institution in the local currency, with the exception of INEI-ANLIS (Argentina), which reported costs in US dollars, due to exchange rate fluctuations in the national currency, and also because part of consumables and equipment (including the sequencer) for that period of time were purchased in the USA in the framework of an international pilot project. Where the local currency was not the Euro, costs were converted into Euro based on the reference exchange rate of the European Central Bank for the relevant year. As the reference periods had a maximum length of one year, no discount rate has been applied.

The following tables provide details on WGS equipment used in the case study institutions, and the conventional methods used as comparator, by institution and pathogen.

Supplementary table S1: Type and total purchase costs of WGS equipment used by case study institutions, by analytical step

		APHA (UK)	FLI (DE)	EMC (NL)	IZSLER (IT)	INEI-ANLIS (ARG)	MDH (USA)	PHAC (CAN)	PHE (UK)
Pathogens		Av. influenza	Avian influenza	Influenza	Foodborne*	Foodborne*	Foodborne*	Foodborne*	Foodborne*
Batch size for sample processing/sequencing		1-2	6	30	24	12	24	32	Processing: 40<br>Sequencing: 96
No. of samples analysed in reference period		26<br>*(in 8 months)*	30<br>*(in 3 months)*	630<br>*(in 5 months)*	175<br>*(in 12 months)*	320<br>*(in 12 months)*	1 767<br>*(in 12 months)*	8 630<br>*(in 12 months)*	15 791<br>*(in 12 months)*
Analytical steps	Sample processing	Basic lab equipment only (€ 0)**	*- Covaris sonicator*<br>*- Agilent bioanalyser*<br>(€ 49 300)	*- Gel electro-phoresis system*<br>(€ 4 000)	Basic lab equipment only**	*- Qiacube DNA*<br>(€ 13 724)	*- MagNA Pure 24*<br>(€ 44 260)	Basic lab equipment only**	*- 2 Qiasymphony*<br>*- Roche Magna Pure 96*<br>(€ 218 582)
	Library preparation	Basic lab equipment only (€ 0)**	Basic lab equipment only (€ 0)**	*- PCR machine*<br>*- Qubit*<br>*- Magnate 96 wells*<br>(€ 8 800)	*- Biorad-T100 thermal cycler*<br>*- Biorad-CFX96 RT-system*<br>*- Microplate Genie-Shake*<br>(€ 29 100)	*- Qubit 3.0*<br>*- Bioshake iQ Thermomixer*<br>(€ 2 943)	*- Multichannel & Single Channel Pipette*<br>(€ 3 203)	*- Tapestation*<br>*- Blue Pippin*<br>*- QUBIT*<br>(€ 51 641)	*- 2 cBot Cluster Generation System*<br>*- 2 LabChip GX*<br>*- 3 Assy-Sciclone G3*<br>*- LabChip-DS Spectrophotometer 96*<br>*- 2 Glomax: 96 well plate Fluorometer*<br>*- Biomek NXP Span-8 with integrated sealer and chilled storage*<br>*- Biomek NXP Span-8*<br>*- 3 Biomek NXP Multichannel*<br>(€ 1 033 590)
	Sequencing	*- Illumina MiSeq*<br>(€ 104 826)	*- IonTorrent PGM*<br>(€ 93 000)	*- GridION*<br>(€ 45 000)	*- Illumina MiSeq*<br>(€ 100 000*)*	*- Illumina MiSeq*<br>(€ 75 273)	*- 2 Illumina MiSeq*<br>(€ 155 624)	- 3 Illumina MiSeq<br>(€ 264 345)	*- 2 Illumina HiSeq*<br>(€ 1 212 821)
	Bioinformatics	*- Workstation*(€ 2 355)	*- Server*<br>(€ 34 700)	*- Server*<br>*- Storage*<br>*- CLC*<br>(€ 16 560)	*- 3 Workstations*<br>*- Storage*<br>*- BioNumerics*<br>(€ 44 220)	*- Server*<br>*- 2 Computers*<br>(€ 26 702)	*- CLC*<br>*- BaseSpace subscription*<br>*- PC*<br>(€ 5 665)	*- Storage*<br>*- Networking*<br>*- Servers*<br>*- BioNumerics*<br>(€ 2 892 662)	*- Computing system*<br>*- Network*<br>*- Storage*<br>(No purchase cost provided)
**Total purchase costs**		**€ 107 181**	**€ 177 000**	**€ 74 360**	**€ 173 320**	**€ 118 641**	**€ 208 751**	**€ 3 208 648**	**€ 2 464 922†**

Source: Own compilation based on case study results. * Foodborne pathogens: *Salmonella* (all), Listeria (IZSLER, PHE, PHAC, MDH), E.coli/shigella (PHE, INEI-ANLIS, MDH), Campylobacter (PHE, MDH), Vibrio (MDH). **Costs for basic laboratory equipment are not included in the assessment. Purchase costs of € 0 therefore imply that no other equipment than basic laboratory equipment was used by the institution. † Not including bioinformatics costs.

Supplementary table S2: Overview of conventional methods used as comparator, by institution and pathogen (with percentage of samples typically analysed using each method)

	APHA (UK)	FLI (DE)	EMC (NL)	IZSLER (IT)	INEI-ANLIS (ARG)	MDH (USA)	PHAC (CAN)	PHE (UK)
**Avian influenza**	Sanger sequencing – HA/NA analysis (100%)	Sanger sequencing – whole genome (100%)	-	-	-	-	-	-
**Influenza A and B**	-	-	Real Time PCR (100%), virus isolation (17%), phenotyping of virus isolates - Hemagglutination inhibition (5%) and/or Virus neutralization (3%) and/or NA-Star (4%) - and Sanger Sequencing of a representative subset (4%)	-	-	-	-	-
Salmonella	-	-	-	Serotyping (100%)<br>PFGE (100%)<br>PCR (50%)<br>MLVA (60%)	Biochemical testing (100%)<br>Serotyping (100%)<br>MaldiTOF (5%)<br>PFGE (100%)	PFGE (100%)	Biochemical analysis (100%)<br>Serotyping (100%)<br>PFGE (65%)	PCR x2 (73%, 10%)<br>MLVA (48%)<br>Serotyping (98%)<br>Phage typing (99%)<br>PFGE (3%)<br>D-Tartrate (3%)<br>Glucose gas (8%)<br>AMR (68%)
**Listeria**	-	-	-	PFGE (100%)	-	PFGE (100%)	Biochemical analysis (100%)<br>PFGE (100%)	PCR x2 (100%)<br>fAFLP (100%)
**E. Coli & Shigella**	-	-	-	-	Biochemical testing (100%)<br>PCR typing (100%)<br>MaldiTOF (5%)<br>PFGE (100%)	PFGE (100%)<br>PCR (100%)	-	PCR (100%)<br>MLVA (100%)<br>Serotyping (100%)<br>Phage typing (100%)<br>Biochemistry (100%)
**Campylo-bacter**	-	-	-	-	-	PFGE (100%)<br>MaldiTOF (100%)	-	PCR (100%)<br>MLST (52%)<br>Serotyping (12%)<br>Phage typing (38%)
**Vibrio**	-	-	-	-	-	PFGE (100%)<br>PCR (100%)	-	-

Source: Own compilation based on case study results. Figures in parentheses are the share of samples typically processed using the method.

2. Benefits

Supplementary table S3: Positive effects of using WGS as experienced by the reference laboratories, assessed on a scale on a scale from 1 (no effect at all) to 5 (very significant positive effect)

		APHA (UK)	FLI (DE)	EMC (NL)	IZSLER (IT)	INEI-ANLIS (ARG)	MDH (US)	PHAC (CAN)	PHE (UK)
Effects of WGS on sampling and sampling strategies experienced	**Simplifications in the type of samples needed**	5	1	1	3	1		1	1
	**Simplifications in sample storage or transport**	–	1	1	–	2	–	1	1
	**Reductions in the number of samples needed**	1	2	1	–	1	–	1	1
	**Reductions in the overall costs of sampling**	–	1	1	–	1	–	1	1

		APHA (UK)	FLI (DE)	EMC (NL)	IZSLER (IT)	INEI-ANLIS (ARG)	MDH (US)	PHAC (CAN)	PHE (UK)
Effects of WGS on analytical results and processes experienced	**More detailed results produced**	5	5	5	5	4	5	5	5
	**Improved accuracy of results produced**	5	–	1	5	4	4	5	5
	**Improved specificity of results produced**	5	–	1	5	4	5	5	5
	**Improved sensitivity of results produced**	5	3	1	5	4	5	5	5
	**Simplified laboratory work flows**	2	1	1	5	4	4	4	5
	**Reduction of staff time**	2	1	3	5	4	2	1	5
	**Reduction of overall costs for analysis**	2	3	–	–	–	1	4	5
	**Reduction of consumables needed for analysis**	2	1	1	5	4	1	3	5
	**Reduction of time needed for analysis**	3	1	1	4	1	1	*	4

		APHA (UK)	FLI (DE)	EMC (NL)	IZSLER (IT)	INEI-ANLIS (ARG)	MDH (US)	PHAC (CAN)	EUR
Effects on research and methods applied as experienced	**Better understanding of disease transmission**	4	5	1	5	4	4	5	5
	**Other benefits for research**	–	4	–	–	–	1	5	5
	**Improvement in epidemiological methods***	4	5	1	5	1	1	–	3

	APHA (UK)	FLI (DE)	EMC (NL)	IZSLER (IT)	INEI-ANLIS (ARG)	MDH (US)	PHAC (CAN)	PHE (UK)
Development of better diagnostic tests	4	2	1	–	4	–	3	2

Effects of WGS on outbreak identification and response experienced

	APHA (UK)	FLI (DE)	EMC (NL)	IZSLER (IT)	INEI-ANLIS (ARG)	MDH (US)	PHAC (CAN)	PHE (UK)
Improved detection that outbreaks are related	4	5	5	5	4	5	5	5
Improved information on outbreak epidemiology	4	5	5	5	4	4	5	5
Earlier detection of an initial outbreak	5	2	5	5	–	–	*	5
Improved info for additional control/ biosecurity measures	4	4	–	5	1	–	5	5
Reduced number of secondary outbreaks	3	5	–	–	1	–	–	3
Reduction of overall costs outbreak identification/response	2	2	–	5	–	1	–	–
Reduction of the duration of an outbreak	2	3	–	–	1	4	–	5
Reduction of the disease burden (humans)	2	–	–	–	1	2	–	5
Reduction of the disease burden (livestock)	2	2	–	–	1	–	–	–

Wider effects of WGS on society as experienced

	APHA (UK)	FLI (DE)	EMC (NL)	IZSLER (IT)	INEI-ANLIS (ARG)	MDH (US)	PHAC (CAN)	PHE (UK)
Reduction of costs of outbreak(s) for the wider society	4	5	–	–	1	–	–	–
Reduction of negative effects of outbreak(s) on trade	4	3	–	5	1	–	–	–
Reduction of negative effects of outbreak(s) on livestock industry	4	2	–	5	1	–	–	–
Reduction of negative effects of outbreak(s) on consumer trust in food	3	2	–	–	1	–	3	4
Reduction of negative effects of outbreak(s) on tourism	4	–	–	–	1	–	–	–

Note: All institutions were asked to assess specific positive effects or impacts of using WGS on a scale from 1 (no effect at all) to 5 (very significant positive effect). *This item was removed from Figure 5 in the article to avoid misinterpretations, as the reasoning provided by case study institutions for indicating it overlapped with other items provided, such as 'Better understanding of disease transmission', ' More detailed results produced', which in turn had a positive effect on epidemiological investigations.

Supplementary table S4: Results of retrospective analyses of past outbreaks with WGS, as reported by case study institutions

Insti-tution	Patho-gen	Region / year	Description of outbreak	Type of study	WGS effect on case definition/detection	Effect on disease control/burden	Other conclusions
**IZSLER (IT)** [a]	Salmonella 4,[5],12:i:-	Emilia-Romagna (2013)	The outbreak was detected by routine surveillance. Epidemiological investigation rapidly identified the food involved (fermented dry-cured salami made from pork) and the facility implicated, but was not able to attribute several individuals infected with the implicated strain to the outbreak and could not confirm or exclude the role of suspect sources at abattoir and farm level. Overall, 137 human isolates with with the outbreak pulsotype were recovered from the outbreak territory	98 Salmonella isolates from human, animal and food sources were re-examined using WGS over a 3-year period (2012–15)	With WGS, first isolates unambiguously linking human cases to the salami facility which had been the source of the outbreak were available more than a month in advance of the outbreak onset (as identified based on incidence) and more than 2 months before the source had been identified using PFGE and MLVA	Had WGS been in routine use at the time of the 2013 outbreak, the source of the outbreak could have potentially been identified up to two months earlier, possibly preventing dozens of infections if the correct mitigation measures had been taken in time	MLVA and PFGE were not only unable to reliably link isolates to the outbreak source, but had in fact produced misleading results by incorrectly classifying some cases as being part of the outbreak when they were not
**INEI-ANLIS (ARG)** [b]	Shigella sonnei	La Pampa (2010-2011)	Two suspected outbreaks in 2010 and 2011 in La Pampa province. In 2010, 26 reported cases were spread throughout the city of General Pico; 9 isolates were recovered. The second outbreak occurred in 2011 in the city of Castex. No supporting epidemiological data is available for the second outbreak, but 7 isolates were recovered. At the time, it was uncertain whether the two outbreaks were related.	17 Shigella isolates from the two outbreaks were re-examined using PFGE and WGS to test for relatedness.	Both PFGE and WGS confirmed that the two outbreaks were independent. However, PFGE over-predicted variability within the genomic structures. WGS confirmed conventional results and provided a more detailed view of the relationships between and within outbreaks.	WGS detected the presence of the ESBL gene OXA-1, which is suspected to be associated with resistence to 3rd generation cephalosporin, a standard treatment for Shigellosis in Argentina.	The retrospective study shows that even with a lack of supporting routine data, WGS becomes an indispensible method for the tracking and surveillance of bacterial pathogens during outbreaks.
**INEI-ANLIS (ARG)** [c]	Shigella sonnei	Country-wide (2010, 2011, 2016)	Three outbreaks of Shigellosis occurred in various locations around the country in 2010 (5 isolates recovered), 2011 (3 isolates recovered) and 2016 (8 isolates recovered).	16 isolates from 3 outbreaks in Argentina were re-examined with WGS to supplement a pan-Latin American study on Shigella sonnei	WGS confirmed the results of conventional tests which suggested that the outbreaks were phylogenetically distinct. WGS also uncovered that the 2011 and 2016 isolates fell into multiple sublineages, indicating that the outbreaks may have multiple epidemiologic origins.	WGS was used to characterise the anti-microbial resistance (AMR) profiles of Shigella sonnei recovered from the three outbreaks at a more granular level, showing increasing levels of AMR in Shigella sonnei across Latin America.	WGS detected the presence of closely related isolates from different countries within an individual sublineage, indicating that international transmission of S. sonnei occurs across Latin America.
**MDH (USA)** [d]	Vibrio parahaem-olyticus	MD (2010)	Two individuals became ill after eating raw oysters in two different restaurants in Baltimore, MD. Isolates were collected from the two individuals as well as nine outbreak-implicated oysters.	Retrospective WGS analysis to determine the identity, genetic	Strains isolated from stool and oyster samples were indistinguishable with PFGE. WGS analysis was able to clarify the phylogenetic relationships of	Not discussed	The wgMLST method employed using WGS was found to be easy, robust, and scalable to multiple strains to be used in

				makeup, relatedness, and potential pathogenicity of the 2010 MD samples	the clinical and food samples and identify the clinical strains as belonging to a clonal complex described only in Asia, confirming their local vector and their likely path from Asia to MD		future *Vibrio parahaemolyticus* outbreak investigations
**MDH (USA)** [e)]	Vibrio parahaem-olyticus	MD (2012-2013)	In the summers of 2012 and 2013, spikes in cases of *Vibrio parahaemolyticus* were reported in the US state of MD. Overall, 46 cases of *Vibrio parahaemolyticus* gastroenteritis associated illnesses were reported over this period, out of which 34 strains could be isolated.	Retrospective analysis of the 34 MD samples as well as other national and international samples for comparison	WGS analysis provided far more precise case definitions than those that had been achieved with PFGE and MLST. Five distinct clusters of sequence types (STs) were detected through WGS. WGS was also able to provide detailed information on sublineages within each cluster, e.g. by differentiating between West Coast and East Coast strains of ST36, which had not been possible with MLST.	Not discussed	In addition to the phylogenomic analysis, WGS was also used to determine the pathogenicity of particular strains
**PHE (UK)** [f)]	Shigella sonnei	UK (2011)	Outbreak in the Orthodox Jewish (OJ) community in the UK. Gastrointestinal illness was reported in 86 people, of whom 82 met the case definition for possible, probable or confirmed outbreak case of *S. sonnei*, across 18 family clusters and six further individuals. Of these, 27 cases were laboratory confirmed at the local laboratory	Retrospectiveassessment of the value of WGS compared to conventional typing methods. Twenty-four isolates were selected for WGS	WGS and SNP analysis facilitated a more precise case definition. WGS analysis showed that the strains implicated in the outbreak formed three phylogenetically distinct clusters. One cluster represented cases associated with recent exposure to a single strain, whereas the other two clusters represented related but distinct strains of *S. sonnei* circulating in the OJ community across the UK	The lack of clarity in conclusions drawn from MLVA prevented (at the time of the outbreak) broadcasting of specific risks associated with the outbreak. Greater confidence that an outbreak was occurring would have facilitated a more pro-active approach to spread public health messages on infection control more effectively	WGS data challenged the conclusions drawn during the initial outbreak investigation and allowed cases of dysentery to be implicated or ruled out of the outbreak that were previously misclassified

Sources: Reported by the case study institutions, and the following publications: a) Morganti, M., et al. (2018). Rise and fall of outbreak-specific clone inside endemic pulsotype of salmonella 4,[5],12:i:-; insights from high resolution molecular surveillance in Emilia-Romagna, Italy, 2012 to 2015. Eurosurveillance, 23(13), 1–11; b) Chinen, I. et al. (2016) 'Whole genome sequencing identifies independent outbreaks of Shigellosis in 2010 and 2011 in La Pampa Province, Argentina', bioRxiv, (April). doi: 10.1101/049940; c) Baker, K. S. et al. (2017) 'Whole genome sequencing of Shigella sonnei through PulseNet Latin America and Caribbean: advancing global surveillance of foodborne illnesses', Clinical Microbiology and Infection, 23(11), pp. 845–853. doi: 10.1016/j.cmi.2017.03.021; d) Haendiges, J. et al. (2016) 'A Nonautochthonous U.S. Strain of Vibrio parahaemolyticus Isolated from Chesapeake Bay Oysters Caused the Outbreak in Maryland in 2010', Applied and Environmental Microbiology, 82(11), pp. 3208–3216. doi: 10.1128/aem.00096-16; e) Haendiges, J. et al. (2015) 'Characterization of Vibrio parahaemolyticus clinical strains from Maryland (2012-2013) and comparisons to a locally and globally diverse V. parahaemolyticus strains by whole-genome sequence analysis', Frontiers in Microbiology, 6(FEB), pp. 1–11. doi: 10.3389/fmicb.2015.00125; f) McDonnell, J., et al. (2013). Retrospective analysis of whole genome sequencing compared to prospective typing data in further informing the epidemiological investigation of an outbreak of Shigella sonnei in the UK. Epidemiology and Infection, 141(12), 2568–75.

Supplementary table S5: Effects of applying WGS for outbreaks in real-time (i.e. not retrospective), as reported by case study institutions

Insti-tution	Patho-gen	Region/ year	Description of outbreak	WGS effect on case definition/detection	Effect on disease control/burden	Other conclusions
**APHA (UK)**	HPAIV (H5N8)	UK (2016-2017)	The outbreak occurred in both wild birds and poultry, with 13 independent introductions to premises across England and Wales.	There had been a clear positive impact of using WGS on the acquisition of metadata for an initial outbreak, especially for the index case.	Information provided by WGS allowed for a better assessment of the public health risk. WGS also allowed for useful supporting information to be disseminated during outbreaks.	Using WGS led to a reduction in the negative effects of outbreaks for the livestock industry, for tourism, for trade, and for the wider society.
**FLI (DE)**	HPAIV (principally H5N8)	Germany (2016-2017)	The outbreak occurred in Lower Saxony in domestic poultry farms. About 30 farms were affected, including several turkey fattening farms.	Benefits of WGS with respect to earlier detection of the initial outbreak were not experienced, as samples had already been positively identified through conventional methods before reaching the case study institution.	Analysis using WGS was able to indicate that transmission occurred not only through wild birds but also through secondary infection between farms, exposing gaps in biosecurity measures.	WGS revealed gaps in biosecurity of operators and led to a reduction in the costs of outbreak(s), including through the reduction of compensation payments, according to the case study institution.
**INEI-ANLIS (ARG)** [a)]	Shigella sonnei	Buenos Aires (2016)	An outbreak of dysentry was detected in Buenos Aires in April 2016 with more than 900 associated cases, including two fatalities.	WGS had no effect on the detection of the outbreak, as the outbreak was already apparent before the samples were collected for analysis. The analysis indicated that this outbreak was mostly distinct from past outbreaks of *S. sonnei* in 2010/11.	None reported	The investigation concluded that maximising the benefit of genomic outbreak data in specific settings requires long term contextual isolate data from organisms isolated locally and internationally
**MDH (US)** [b)]	*Salmonella* (multiple strains)	23 US states (2017)	A multistate outbreak of *Salmonella* was ultimately linked to imported papayas from a farm in Mexico. A total of 220 people were infected, of which 68 were hospitalised and one death was reported in New York.	WGS was used to show that the infections across more than 20 states were genetically linked. WGS was also able to distinguish between four independent outbreaks of various *Salmonella* strains linked to Mexican papayas that were ongoing during the same period.	WGS was used to link isolates of *Salmonella* collected from grocery store papayas to clinical isolates from affected persons. On the basis of the WGS results, papayas imported from Mexico were recalled by several producers.	WGS was used to test for anti-microbial resistance. WGS did not identify anti-microbial resistance genes among isolates from 139 ill people. However, one ill person's isolate, a *Salmonella* Senftenberg, contained a gene known to decrease susceptibility to ciprofloxacin.
**PHAC (CAN)**[e)]	*Salmonella* Enteritidis	Canada (2017 to 2019)	Outbreaks linked to frozen breaded raw chicken products. As of May 2019, a total of 573 laboratory-confirmed cases have been investigated. 96 individuals were hospitalised and 3 deaths were reported, although it was unclear to what extent *Salmonella* had contributed to those three deaths.	WGS led to the detection of the 17 *Salmonella* outbreaks linked to raw chicken. 14 of these outbreaks were detected in the first 6 months of using WGS for routine surveillance in 2017.	Based on WGS data, 14 food products were linked to the outbreaks. 13 products were recalled by food inspection authorities and one product was voluntarily removed by the retailer. In 2018, the Government of Canada adopted new, stricter requirements for this type of products	The use of WGS allowed food safety authorities to identify an entire product category (raw frozen breaded chicken) that was responsible for a disproportionate amount of the *Salmonella* enteritidis disease burden.
**PHE (UK)** [c)]	*Salmonella* Enteritidis	UK and Spain (2015)	The outbreak was detected [in the UK] using WGS data. 136 cases were identified in the UK and 18 in Spain. One isolate from a food containing chicken eggs [omlette] was within the outbreak cluster.	The investigation concluded that that UK and Spanish cases were exposed to a common source of *Salmonella*-contaminated chicken eggs. Using WGS provided additional sensitivity over phage-typing. Of the UK cases, 68% corresponded to PT59. Had WGS not been used, it is likely that the outbreak would still have been recognized. [However,] the loss	Routine WGS changed the way the outbreak was managed; it was previously accepted practice in infectious intestinal disease outbreaks to exclude cases with travel history to focus on possible in-country exposures. With the greater specificity of WGS information, travel histories and other geographical metadata can now provide	One of the limitations of the investigation was that it did not isolate *Salmonella* from any of the eggs sampled during the outbreak investigation. Food chain information was difficult to obtain. This situation arose both from the challenges in contacting cases and poor recall, but also the very resource-intensive nature of trace

				of 32% of cases would have likely slowed the recognition of the outbreak.	information on which other countries may have cases from the same source.	back investigations.
**PHE (UK)** d)	*Salmonella* Enteritidis	UK (detected 2015)	Analysis of WGS data uncovered the previously undetected outbreak that had been on-going for four years. Between April 2014 and September 2015, 714 isolates of S. Enteritidis PT8 were reported and 147 fell within the five SNP outbreak cluster.	Following the implementation of SNP typing at PHE, analysis of WGS data revealed a large sub-cluster of isolates of S. Enteritidis PT8 associated with an outbreak of salmonellosis. A coordinated investigation showed that the outbreak was linked to handling of feeder mice or snakes infected by the mice. The outbreak had been occurring undetected by traditional surveillance procedures since at least January 2012.	A series of recommendations were made to control infections at the farm level and point of sale. The Reptile Trade Association produced their own advice which was sent to all major suppliers of reptile feed.	The investigation highlighted the potential of WGS to have a - clear impact on decreasing the incidence of *Salmonella* by identifying low level, continuous transmission ("slow burn") outbreaks. SNP typing of the core genome provided evolutionary context making it possible to confidently link cases from four years earlier to the contemporary cluster.

Sources: Case study reports, and the following publications: a) World Health Organization (WHO) (2018) Annex 2. Contribution of Whole Genome Sequencing to the National Surveillance of Shigella sonnei in Argentina.; b) Centers for Disease Control and Prevention (2017) Multistate Outbreak of *Salmonella* Infections Linked to Imported Maradol Papayas (Final Update). https://www.cdc.gov/*Salmonella*/kiambu-07-17/index.html; c) Inns, T., et al. (2017). Prospective use of whole genome sequencing (WGS) detected a multi-country outbreak of *Salmonella* Enteritidis. Epidemiology & Infection, 145(2), 289-298; d) Kanagarajah, S., et al. (2018). Whole genome sequencing reveals an outbreak of *Salmonella* Enteritidis associated with reptile feeder mice in the United Kingdom, 2012-2015. Food Microbiology, 71, 32-38. g) Public Health Agency of Canada (2019), Public Health Notice - Outbreaks of *Salmonella* infections linked to raw chicken, including frozen raw breaded chicken products. https://www.canada.ca/en/public-health/services/public-health-notices/2018/outbreaks-*Salmonella*-infections-linked-raw-chicken-including-frozen-raw-breaded-chicken-products.html.

3. Breakeven analysis

This section presents the results of the breakeven analysis, which applies a cost of illness approach to determine the number of cases of illness that would need to be avoided through the use of WGS in order for the introduction of WGS to be cost-neutral from a public health perspective. It first provides contextual information on the burden of illness for the chosen pathogen (Salmonella) in the case study countries. The cost of illness is calculated. The results of the breakeven analysis are then presented and discussed in relation to the burden of illness.

As discussed in the previous section, the use of WGS for pathogen identification and surveillance is considered by the case study institutions to have positive effects on analytical results and processes (e.g. through providing higher-definition results) and on outbreak investigation and response (e.g. through improved epidemiological analysis). In the long run, the use of WGS is expected to lead to a reduction in the number of cases of illness, and thus in the disease burden, due to a better-targeted response. The breakeven analysis presented in this section aims to estimate the number of cases of illness that would need to be avoided each year through the use of WGS in order to 'break even' on costs, i.e. in order to make the use of WGS cost-neutral.

The breakeven analysis calculates the cost of illness in terms of health care utilisation costs, productivity loss, and premature death, and compares this to the additional cost of using WGS. As the analysis focuses only on offsetting the cost of illness and does not take into account additional benefits of using WGS for pathogen identification and surveillance in terms of e.g. effects on research, trade, or industry, its results should be understood to be a conservative estimate.

The costs of illness are pathogen-specific, and therefore the breakeven analysis is carried out at the pathogen level. The assessment focuses on *Salmonella*, as all case study institutions in this study dealing with foodborne pathogens use WGS to sequence *Salmonella* samples. There is also an existing European and international body of work dealing with the costs of salmonellosis infection in depth,[1] making this pathogen the most suitable candidate for the breakeven analysis.

Data on confirmed cases of salmonellosis is essential for the breakeven analysis by indicating the scale of the burden of illness. Data on confirmed salmonellosis cases is presented in the following table for the geographical jurisdictions covered by each case study institution, including data on the approximate number of annual hospitalisations and deaths where this information is available.

---

[1] Burden of illness and cost of illness estimations for *Salmonella* have been made by public health authorities such as DG SANCO in the EU, the Centers for Disease Control and Prevention (CDC) and US Department of Agriculture (USDA) in the United States, PHAC in Canada, and the Food Standards Agency in the UK. These estimates are cited in the following subsections.

Supplementary table S6: Burden of illness (salmonellosis), by case study jurisdiction

Case study jurisdiction	Number of cases reported annually (3-yr average)	Approx number of hospitalisat-ions reported annually	Approx number of deaths reported annually
**Italy – Emilia-Romagna** (IZSLER)	276 *	n.a.	n.a.
**Argentina** (INEI-ANLIS)	758	n.a.	n.a.
**UK – England, Wales and Northern Ireland** (PHE)	8 770	968 *	52 *
**Canada** (PHAC)	7 665	925	17
**US - Maryland** (MDH)	906	273	3

Sources: IT - ECDC (2018), *The European Surveillance System (TESSy)*; UK - PHE (2018), *Salmonella data 2007 to 2016* and ECDC, *Surveillance Atlas for Infectious Diseases, http://atlas.ecdc.europa.eu/public/index.aspx (2018)*; CAN - PHAC (2018), *Reported cases from 1924 to 2016 in Canada - Notifiable diseases on-line* and PHAC (2016), *Yearly food-borne illness estimates for Canada*; US - CDC (2018), *FoodNet Fast*; Argentina - *Laboratory Surveillance System (SIVILA) of the National Health Surveillance System*. Notes: Data provided on cases of salmonellosis refer to the geographical jurisdictions of the institution as indicated in the case study report. Where a case study institution processes samples originating from the whole country (Canada, Argentina), data on salmonellosis refer to the country as a whole. Where a case study institution only processes samples from a specific geographical region within a country, data on salmonellosis refer to this particular region (England, Wales and Northern Ireland in the UK, Emilia-Romagna in Italy, and Maryland in the US). * Regional data approximated as a population-based proportion of national data, as no regional data was available.

The table above shows how the burden of illness varies across case study jurisdictions. In the largest jurisdiction by population, England, Wales and Northern Ireland (PHE), an average of 8 770 confirmed salmonellosis cases were reported annually between 2015 and 2017, of which approximately 968 were serious enough to require hospitalisation and 52 resulted in the death of the patient. In contrast, in the smallest jurisdiction by population, the Italian region of Emilia-Romagna (IZSLER), an average of 276 cases were reported annually between 2015 and 2017, and no data is available on the number of associated hospitalisations or deaths.

However, it is important to note that confirmed cases of salmonellosis as presented in the table above are not equivalent to the total number of cases in the community, and understate the actual prevalence of salmonellosis (and thus the actual burden of disease) in any given jurisdiction. Although salmonellosis is a notifiable disease, meaning that confirmed cases of infection are required by law to be reported to public health authorities, a number of steps must be achieved in order for the case to be recorded in national surveillance statistics. The relationship between the (observed) number of reported cases and the (unobserved) total number of infections or exposures in the community can be illustrated through the use of surveillance pyramid, such as the one depicted below.

Supplementary figure S1: Surveillance pyramid for nofitiable diseases (foodborne pathogens)

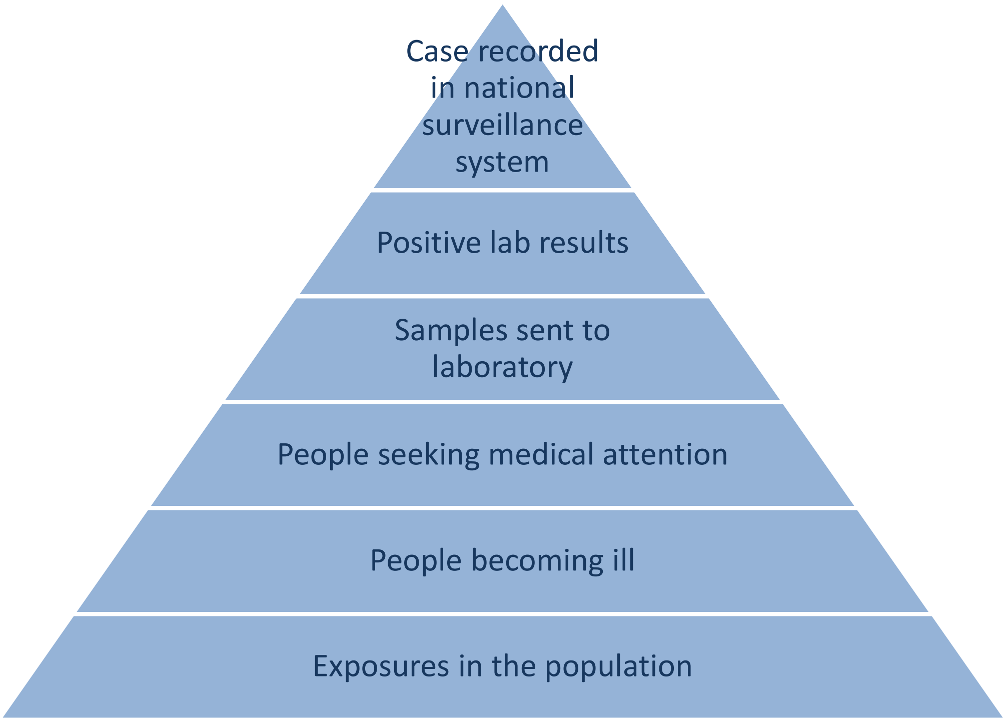

Source: Adapted from EFSA, *Scientific Opinion of the Panel on Biological Hazards on a request from the European Commission on a quantitative microbiological risk assessment on Salmonella in meat* (2008), and the CDC's *Foodborne Diseases Active Surveillance Network (FoodNet)* (2015).

As shown in the surveillance pyramid above, cases are only recorded in national statistics where the patient has chosen to seek medical attention, and where a sample has been taken by the health care provider and produced a positive result. This means that 'mild' cases of salmonellosis, in which patients simply recover at home and do not seek out medical care, are excluded from national statistics by definition. Even where patients do seek out medical care, cases will only be included in national statistics for salmonella when the health care provider takes a clinical sample, and where the laboratory results are positive for salmonella (instead of e.g. inconclusive). As a result, the number of confirmed cases of salmonellosis reported in national statistics are only a subset of total cases in the community.

Previous studies have generated multipliers at key levels of the surveillance pyramid in order to estimate the unobserved number of cases in the community. In the EU, for example, these community multipliers have been estimated to range between 3.2 and 16.5, with an average value of 7.3.[2] This estimate suggests that for every 1 case recorded in national statistics, there are approximately 7.3 cases occurring in the community, most of which remain unreported. Given the assumptions and uncertainties involved in calculating the total burden of illness based on community multipliers, we have chosen to focus only on *confirmed* cases of salmonellosis in the breakeven analysis. The fact that a larger number of cases are estimated to go unreported, however, means that the results derived in the following subsection reflect highly conservative estimates of the burden of illness.

---

[2] DG SANCO, *Analysis of the costs and benefits of setting a target for the reduction of Salmonella in breeding pigs* (2011), p. 23-6.

3.1. Cost of illness calculation

Calculating the average cost of a foodborne illness is a highly complex task which requires non-trivial choices to be made regarding which cost elements to include and which elements to leave out. Our approach closely follows (with some adaptations) the methodology used in the cost-benefit analyses of reducing *Salmonella* in breeding pigs and slaughter pigs, which were conducted for DG SANCO in 2010 and 2011 in close coordination with the European Food Safety Authority (EFSA).[3] It also draws on the latest cost of illness model developed by the US Department of Agriculture (USDA).[4]

Salmonellosis infections can result in a number of different outcomes for patients, ranging from mild cases (in which the patient does not seek medical care and recovers at home) to severe cases (in which the patient is hospitalised). In rare cases, salmonellosis infections can also result in death. In order to calculate the cost of illness for an 'average' salmonellosis infection, our approach divides these potential outcomes into different 'severity levels'. Each severity level is associated with different costs, which result from different levels of health care utilisation, time missed from work, and the cost of premature death. After calculating the costs for each severity level, the costs per severity level are then weighted by the relative likelihood of each outcome in order to come up with one 'average' cost of a salmonellosis infection.

Following the approach used in the EC study and the USDA's cost estimates, the cost of illness model for *Salmonella* distinguishes between four different severity levels for the outcome of a salmonellosis infection:[5]

- Level 1: The patient does not visit a physician and recovers from the infection.
- Level 2: The patient visits a physician and subsequently recovers from the infection.
- Level 3: The patient is hospitalised and subsequently recovers from the infection.
- Level 4: The patient is hospitalised and dies.

The four possible outcomes can be illustrated in the form of an infection tree, along with estimates of the relative outcome distribution (i.e. what proportion of cases will result in each outcome). We use the same outcome distributions as the 2011 DG SANCO study,[6] which was adapted for the European context from the USDA model[7] based on consultations with the public health authorities of EU Member States. The *Salmonella* infection tree with the outcome distribution among the severity levels is presented in the figure below.

---

[3] DG SANCO/FCC Consortium (2010), *Analysis of the costs and benefits of setting a target for the reduction of Salmonella in slaughter pigs – Final report*; DG SANCO/FCC Consortium (2011), *Analysis of the costs and benefits of setting a target for the reduction of Salmonella in breeding pigs – Final report*.

[4] USDA Economic Research Service (2014), *Cost estimates of foodborne illnesses*. https://www.ers.usda.gov/data-products/cost-estimates-of-foodborne-illnesses.aspx

[5] In order to simplify the analysis, it is assumed that the patients with outcomes of severity levels 1-3 make a full recovery and do not suffer from longer-term effects (chronic sequelae) such as reactive arthritis.

[6] DG SANCO/FCC Consortium (2011), *Analysis of the costs and benefits of setting a target for the reduction of Salmonella in breeding pigs – Final report*, p. 23-8.

[7] The USDA cost of illness model assumes different outcome distributions than the ones used in the 2011 DG SANCO study, which would have a - notable effect on the results if applied. See the discussion regarding sensitivity analysis.

Supplementary figure S2: Infection tree for Salmonella with outcome distribution

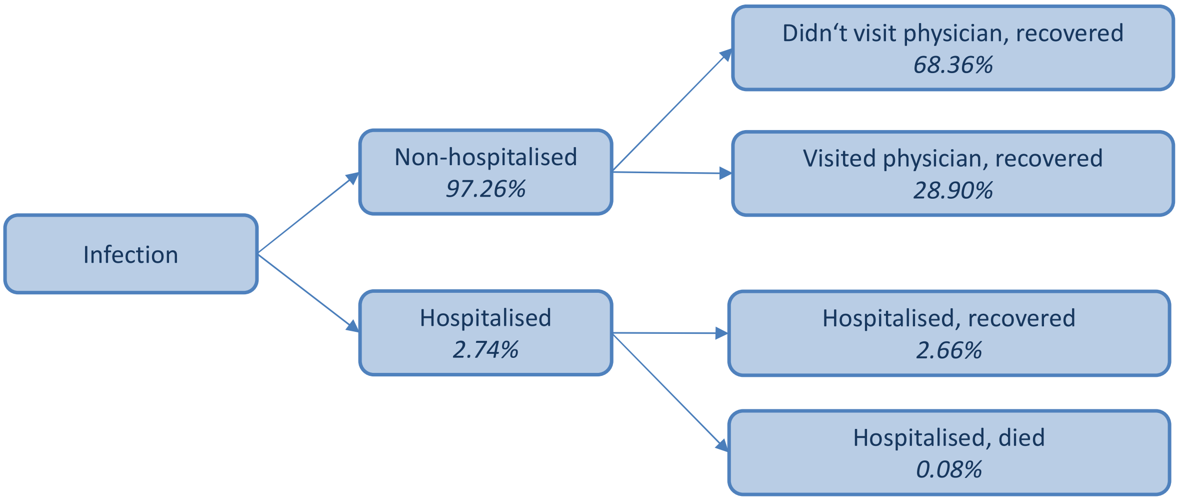

Source: Adapted from DG SANCO/FCC Consortium (2011), *Analysis of the costs and benefits of setting a target for the reduction of Salmonella in breeding pigs – Final report*.

As shown in the figure above, the most common outcome for a salmonellosis infection is that the patient does not visit a physician and makes a full recovery (68.4% of cases), followed by the outcome in which the patient visits a physician and then recovers (28.9% of cases). In total, therefore, the vast majority of salmonellosis cases (97.3%) do not result in hospitalisation. An estimated 0.08% of total infections result in the death of the patient.

As indicated previously, each of the four severity levels are associated with different levels of costs. The following three types of costs are considered in the cost of illness model:

- Health care utilisation;
- Productivity loss; and
- Premature death.

The following subsections address each of these cost types in turn.

Health care utilisation

Health care utilisation costs include the costs of physicians' visits, emergency room visits, outpatient clinic visits, and hospitalisation. In order to calculate the health care utilisation costs per severity level, we first estimate the type and amount of health care services accessed by patients at each severity level, and then multiply this by the unit cost for each type of health care service. As health care costs vary across jurisdictions, the unit cost of various health care services is estimated separately for each case study country.

For estimating the amount of health care services used at each severity level, we use the service utilisation assumptions by Frenzen et al (1999),[8] which have been used without adaptation in both the DG SANCO studies and the USDA estimates. These assumptions are presented for each severity level in the following table.

---

[8] Frenzen et al (1999), '*Salmonella* Cost Estimate Updated Using FoodNet Data.' *FoodReview* (22)2: 10-15.

Supplementary table S7: Health care service utilisation assumptions, by severity level

Severity level	Physicians' visits	Emergency room visits	Outpatient clinic visits	Hospitalisation
**Didn't visit physician, recovered**	0	0	0	0
**Visited physician, recovered**	1.4	0.1	0.3	0
**Hospitalised, recovered**	0.7	0.3	0.2	1.0
**Hospitalised, died**	1.0	0.3	0.2	0.9

Source: Frenzen et al (1999), '*Salmonella* Cost Estimate Updated Using FoodNet Data.' *FoodReview* (22)2: 10-15.

As shown in the table above, the lowest severity level (didn't visit physician, recovered) is assumed to make no use of health care services, while the three higher severity levels are assumed to make at least some use of various health care services depending on the severity of the case.

The unit costs for each form of health care service accessed have been adapted from the 2010 DG SANCO study and inflated to 2017 (Euro) values using Eurostat's labour cost index.[9] Average costs at the EU28 level are considered to form the 'base costs'. In order to adjust these base costs for each of the case study countries, the base costs are multiplied by a country index for each of the foodborne surveillance case studies, which is based on the ratio of average gross earnings in each country to the EU28 average.[10] An exception to this approach is the USA, as the USDA has provided its own cost estimates for the exact same service types in the context of a nearly-identical model; in this case, the USDA estimates are converted into EUR and taken as given. The table below shows the unit cost assumptions for health care services used for each case study country, with the EU28 base costs presented for reference.

[9] Eurostat, *Labour cost index by NACE Rev. 2 activity - nominal value, annual data* [lc_lci_r2_a]. Extracted 14 January 2019. The Eurostat labour cost index was used in the 2010 and 2011 DG SANCO studies to inflate service utilisation costs and is used here for the same purpose, as the consumer price index (HICP) focuses on consumer goods and is not considered appropriate for health care costs.

[10] Source of data for average gross earnings is Eurostat, *Annual net earnings* [earn_nt_net] (using 'Gross earnings' variable) extracted 14 January 2019, for the UK, Italy, and the USA; and ILOSTAT (Average monthly earnings of employees) for Canada and Argentina. The index is constructed around a base value of EU28 = 1.00.

Supplementary table S8: Unit cost assumptions for health care services, by case study country (in EUR 2017)

Case study country	Country index	Physicians visit	Emergency room visit	Outpatient clinic visit	Hospitalisation
**EU28 (Base)**	1.00	€ 28.41	€ 113.65	€ 170.47	€ 2 841.15
**UK**	1.42	€ 40.45	€ 161.81	€ 242.71	€ 4 045.13
**Italy**	0.88	€ 24.99	€ 99.96	€ 149.94	€ 2 498.95
**Canada**	0.93	€ 26.53	€ 106.11	€ 159.16	€ 2 652.68
**US**	N/A	€ 110.47	€ 465.53	€ 535.32	€ 11 325.15
**Argentina**	0.21	€ 6.01	€ 24.03	€ 36.04	€ 600.64

Source: Base values adapted from DG SANCO/FCC Consortium (2010), *Analysis of the costs and benefits of setting a target for the reduction of Salmonella in slaughter pigs – Final report,* p. 90. Country index for Canada and Argentina compiled based on wage costs from ILOSTAT, Annual monthly earnings of employees. USA cost figures are taken from USDA Economic Research Service (2014), *Cost estimates of foodborne illnesses*, converted to EUR and inflated to 2017 using Eurostat's labour cost index [lc_lci_r2_a].

As the table above shows, hospitalisation has the highest unit cost among the various health care services, with a base cost of EUR 2 841 for the EU28. Physicians' visits are the least costly, with a base cost of EUR 28.41. Based on the data provided by the USDA, unit costs for health services in the USA are markedly higher than in the other case study countries, with unit costs ranging from EUR 110 for a physicians' visit to EUR 11 325 for hospital admittance.

The country-adjusted service utilisation costs in the table above are multiplied by the service utilisation rates in the previous table to generate the total health care utilisation costs at each severity level per case study. These costs are indicated in the table below. The right-most column shows the weighted average health care utilisation costs per case study, taking into account the relative outcome distribution.

Supplementary table S9: Total health care utilisation costs per severity level, by case study country (in EUR 2017)

Case study country	Severity level				Weighted average health care utilisation cost
	Didn't visit physician, recovered *(68.36% of cases)*	Visited physician, recovered *(28.90% of cases)*	Hospitalised, recovered *(2.66% of cases)*	Hospitalised, died *(0.08% of cases)*	
**UK**	€ 0.00	€ 145.62	€ 4 170.53	€ 3 778.15	**€ 156.04**
**Italy**	€ 0.00	€ 89.96	€ 2 576.42	€ 2 334.02	**€ 96.40**
**Canada**	€ 0.00	€ 95.50	€ 2 734.91	€ 2 477.60	**€ 102.33**
**US**	€ 0.00	€ 361.81	€ 11 649.20	€ 10 549.83	**€ 422.87**
**Argentina**	€ 0.00	€ 21.62	€ 619.26	€ 561.00	**€ 23.17**

Source: Own calculation.

As indicated in the table above, the four severity levels show considerable differences in health care utilisation costs, with the two hospitalisation outcomes incurring markedly higher costs than either of the non-hospitalisation outcomes. As the hospitalisation outcomes collectively represent fewer than 3% of total cases, however, and more than half of all cases incur no health care expenses at all, the weighted average health care utilisation costs are bought down to a range between EUR 96.40 and EUR 422.87, with an average base cost of EUR 109.60 for the EU28. As noted

previously, the highest costs at all severity levels are reported in the US, due to the higher unit costs for health care services.

Productivity loss

The costs of productivity loss are equal to the value of missed time from work. This is calculated first by estimating the number of days missed from work due to a salmonellosis infection, which is assumed to vary by severity level, and then multiplying the number of missed days by the average gross daily earnings in each case study country. Finally, in order to account for the fact that not all patients are employed, we multiply these costs by the proportion of the population in each country that is economically active to get country-specific estimates of the cost of lost productivity.

Assumptions regarding the number of days missed from work due to a salmonellosis infection for each severity level are based on the estimates used in the DG SANCO studies. These are presented in the table below.

Supplementary table S10: Days missed from work per severity level

	Didn't visit physician, recovered	Visited physician, recovered	Hospitalised, recovered	Hospitalised, died
**Days missed from work**	0.5	1.6	4.5	4.5

Source: DG SANCO/FCC Consortium (2010), *Analysis of the costs and benefits of setting a target for the reduction of Salmonella in slaughter pigs – Final report*.

As the table above shows, the number of days missed from work starts with 0.5 for the lowest severity level and increases to 4.5 for both of the hospitalisation levels.

The monetary value of time missed from work depends on the average gross wage, which varies across the case study countries. The table below shows the average gross daily earnings per case study country.

Supplementary table S11: Average gross daily earnings, by case study country (in EUR 2017)

Case study countries	Average gross daily earnings
**UK**	€ 199.40
**Italy**	€ 123.18
**Canada**	€ 130.76
**US**	€ 184.58
**Argentina**	€ 29.61

Source: Eurostat, *Annual net earnings* [earn_nt_net] (variable: 'Gross earnings'), extracted 14 January 2019, for the UK, Italy and the USA; ILOSTAT for Canada and Argentina. All figures inflated to 2017 using Eurostat's labour cost index [lc_lci_r2_a] and converted to EUR where necessary.

Finally, the costs of productivity loss accrue only to economically active persons. The proportion of economically active persons can be calculated by multiplying the labour market participation rate by the proportion of the total population that is of working age (15-64). As these factors vary across countries, this calculation has been performed separately for each case study country. The table below shows the proportion of the population which is economically active by case study country.

Supplementary table S12: Economically active population per case study country

Case study countries	Working age as proportion of total population	Labour market participation rate	Proportion of cases economically active
**UK**	0.641	0.776	0.497
**Italy**	0.641	0.654	0.419
**Canada**	0.670	0.785	0.526
**US**	0.657	0.733	0.481
**Argentina**	0.639	0.674	0.431

Source: Eurostat, *Population structure and aging* [demo_pjanind] and *Employment and activity by sex and age - annual data* [lfsi_emp_a], both extracted 14 January 2019, for the UK and Italy; ILOSTAT, *Population by sex and age* and *Labour force participation rate by sex and age* for the US, Canada and Argentina.

In order to calculate the total costs of productivity loss for each case study country, the number of days missed from work at each severity level is multiplied by the gross daily earnings and by the proportion of cases that are assumed to be economically active. The weighted average costs of productivity loss are then calculated by weighting the costs at each severity level by the outcome distribution value. These costs are shown in the table below per severity level and in total.

Supplementary table S13: Total costs of productivity loss per severity level, by case study country (in EUR 2017)

Case study countries	Severity level				Weighted average cost of productivity loss
	Didn't visit physician, recovered *(68.36% of cases)*	Visited physician, recovered *(28.90% of cases)*	Hospitalised, recovered *(2.66% of cases)*	Hospitalised, died *(0.08% of cases)*	
**UK**	€ 49.59	€ 158.69	€ 446.32	€ 446.32	**€ 91.99**
**Italy**	€ 25.82	€ 82.62	€ 232.38	€ 232.38	**€ 47.90**
**Canada**	€ 34.38	€ 110.02	€ 309.44	€ 309.44	**€ 63.78**
**US**	€ 44.43	€ 142.17	€ 399.85	€ 399.85	**€ 82.41**
**Argentina**	€ 6.38	€ 20.41	€ 57.40	€ 57.40	**€ 11.83**

Source: Own calculation.

As indicated in the table above, productivity loss is higher for the two hospitalisation outcomes than for the two non-hospitalisation outcomes. As with the health care utilisation costs, the weighted average cost is brought down considerably by the fact that the hospitalisation cases are a small proportion of total cases (less than 3%). The total weighted average cost of productivity loss ranges from a low of EUR 47.90 to a high of EUR 91.99.

Premature death

The costs of premature death accrue only to the 0.08% of cases that fall into the highest severity level. The question of how to calculate a monetary value for a statistical human life is highly controversial. Common methods employed to quantify this figure include the 'value of a statistical life' (VOSL) method, which is derived from individuals' willingness-to-pay (WTP) for a lower risk of death, the 'value of a statistical life year' (VOLY) method, which measures the WTP for an additional year of

life expectancy, and the human capital method (HC), which measures the loss of projected future earnings.

Estimates of the value of a human life based on the methods listed above have been calculated for use in cost-effectiveness analyses by numerous European, national, and international authorities. Selected estimates are presented in the table below.

Supplementary table S14: Various cost-effectiveness estimates for the value of a human life

Source	Estimate	Method
**DG SANCO study (2011)**	€ 60 000 to 1 million (in EUR 2011)	HC
**USDA**	$ 1.6 million to 15.7 million, mean value $ 8.7 million (in USD 2013)	VOLY
**UK Green Book/Dept of Transport**	£ 1.9 million (in GBP 2018)	VOLY/HC
**OECD**	$ 1.8 million to 5.4 million (in USD 2005)	VOSL
**European Chemicals Agency (ECHA)**	€ 3.5 million (lower estimate; EUR 2012)<br>€ 5.0 million (higher estimate)	VOSL

Source: DG SANCO/FCC Consortium (2011), *Analysis of the costs and benefits of setting a target for the reduction of Salmonella in breeding pigs – Final report*; USDA Economic Research Service (2014), *Cost estimates of foodborne illnesses;* UK Department of Transport (2018), *WebTAG Databook* [data supplement to the Green Book]; OECD (2012), *Mortality risk valuation in environment, health and transport policies*; ECHA (2016), *Willingness-to-pay values for various health endpoints associated with chemicals exposure*. See also footnotes 11 and 12.

As illustrated in the table above, the estimated value of a human life for the purpose of cost-effectiveness analysis varies between sources and across methods, ranging from a low of EUR 60 000 in the 2011 DG SANCO study to a high of 15.7 million USD (approximately EUR 13.8 million) used as a higher-bound estimate by the USDA. While some sources, such as the UK government, prefer to use a standard value for all valuations of human life, other sources allow values to vary based on different levels of WTP (which is largely driven by income or wealth) or based on the value of lost productivity (which is determined by local wage rates).

In the 2011 DG SANCO study, the cost of a premature death was based on a HC approach examining the value of lost productivity, which generated values of a human life ranging from EUR 60 000 to 1 million, depending on the local wage rate in each country. In contrast, for the current study we apply a standard value of a human life across all case studies. The reasons for this are as follows: firstly, WTP-based approaches are more common than human capital approaches for this type of assessment, as illustrated in the table above; secondly, applying very different values of life in different countries based on income levels raises issues regarding equity with respect to human life; thirdly, because premature death is so costly compared to other factors that it is the single most influential cost component in the cost of illness analysis (see the next section), the results of the analysis are very sensitive to any country differences in the value of human life, which would reduce comparability between the case study results. We have therefore chosen to use the reference values calculated by the European Chemicals Agency (ECHA), which are presented in the table above.[11] We use and average value of EUR 4.6 million (in 2017 EUR) as a

[11] The ECHA values are also provided as reference in the European Commission's Better Regulation Toolbox, the standard guidance for assessing interventions at EU level. See *European Commission (2017), Better Regulation Toolbox – Tool #31: Health Impacts*.

standard assumption for the cost of a premature death across all case studies, while retaining the low and high estimates for later sensitivity analysis.[12]

3.2. Average cost per case of salmonellosis

The table below shows the estimated cost per case of salmonellosis at each level of severity for each case study.

Supplementary table S15: Cost of a salmonellosis infection per severity level, by case study country (in EUR 2017)

Case study countries	Severity levels			
	Didn't visit physician, recovered (68.36% of cases)	Visited physician, recovered (28.90% of cases)	Hospitalised, recovered (2.66% of cases)	Hospitalised, died (0.08% of cases)
**UK**	€ 49.59	€ 304.32	€ 4 616.85	€ 4 640 974.47
**Italy**	€ 25.82	€ 172.58	€ 2 808.79	€ 4 639 316.39
**Canada**	€ 34.38	€ 205.52	€ 3 044.35	€ 4 639 537.04
**US**	€ 44.43	€ 503.98	€ 12 049.05	€ 4 647 699.68
**Argentina**	€ 6.38	€ 42.03	€ 676.66	€ 4 637 368.40

Source: Own calculation.

As can be seen in the table above, the estimated cost per case of salmonellosis varies considerably by severity level. Costs at the lowest severity level comprise only the costs of productivity loss, and range between approximately EUR 26 and EUR 50. The costs of an infection then rise with the severity level and peak with the outcome of patient death at the highest severity level, with a total cost of approximately EUR 4.6 million per case for all case studies.

The average cost per generic case of salmonellosis, weighted by the outcome distribution values for each severity level, ranges from EUR 3 854 to EUR 3 957, depending on the country. However, the more relevant value for the breakeven analysis is in fact the average cost of a *reported* case of salmonellosis, since this is the base against which the number of cases to be avoided will be compared. By definition, reported cases of salmonellosis exclude all patients in the lowest severity category, since these patients do not enter the health care system and are therefore not registered in surveillance statistics (see the discussion above in section 7.1). The table below shows the average cost of a reported case of salmonellosis, which is calculated by dropping severity level 1 and rebasing the outcome distribution.[13]

---

[12] Note that for this calculation, we have inflated the ECHA estimates for premature death of € 3.5 million (lower estimate; EUR 2012) and € 5.0 million (higher estimate; EUR 2012) to EUR 2017 by using the Euroastat labour cost index. In this way, we derived at a lower estimate of € 3.9 million (in EUR 2017) and the higher estimate for premature death of € 5.5 million (in EUR 2017), with an average value of € 4.6 million (in EUR 2017), which is used in the main part of the breakeven analysis.

[13] Some patients in severity levels 2-4, but especially at severity level 2, will also not be recorded in national statistics; see the surveillance pyramid and related discussion in section 7.1. However, in order to make the calculation more straightforward and avoid unnecessary guesswork, we have decided simply to drop severity level 1 and rebase the outcome distribution on that basis.

Supplementary table S16: Cost of a reported salmonellosis infection per severity level, by case study country (in EUR 2017)

Case study countries	Severity levels			Average cost of a reported case (weighted)
	Visited physician, recovered	Hospitalised, recovered	Hospitalised, died	
	(91.34% of reported caes)	*(8.41% of reported cases)*	*(0.25% of reported cases)*	
**UK**	€ 304.32	€ 4 616.85	€ 4 640 974.47	**€ 12 400.55**
**Italy**	€ 172.58	€ 2 808.79	€ 4 639 316.39	**€ 12 124.03**
**Canada**	€ 205.52	€ 3 044.35	€ 4 639 537.04	**€ 12 174.48**
**US**	€ 503.98	€ 12 049.05	€ 4 647 699.68	**€ 13 224.76**
**Argentina**	€ 42.03	€ 676.66	€ 4 637 368.40	**€ 11 820.61**

Source: Own calculation.

As the table above shows, the average cost of a reported case of salmonellosis is considerably higher than the average cost of a generic case, ranging from EUR 11 821 to EUR 13 225. The table below further deconstructs this cost according to the individual cost components for each case study country.

Supplementary table S17: Average cost per reported case of salmonellosis, by case study country, deconstructed by cost component (in EUR 2017)

Case study countries	Health care utilisation	Productivity loss	Premature death	Average cost (weighted)
**UK**	€ 493.19	€ 183.60	€ 11 723.77	**€ 12 400.55**
**Italy**	€ 304.67	€ 95.59	€ 11 723.77	**€ 12 124.03**
**Canada**	€ 323.42	€ 127.29	€ 11 723.77	**€ 12 174.48**
**US**	€ 1 336.51	€ 164.48	€ 11 723.77	**€ 13 224.76**
**Argentina**	€ 73.23	€ 23.61	€ 11 723.77	**€ 11 820.61**

Source: Own calculation.

As indicated in the table above, the largest single cost component in the average cost per reported case of salmonellosis is premature death. Despite a rate of occurrence of just 0.25% among reported cases, death comprises at least 95% of the total average cost in every case study country except the US, where it only comprises 89% of the average cost of a reported case due to the higher costs of health care utilisation. The dominant role played by death is due to the large value placed on a human life (EUR 4.6 million), which, even at a low rate of occurrence, overshadows most country-specific differences in the costs of health care or productivity loss.

3.3. Results of the breakeven analysis

As discussed in the previous section, the aim of the breakeven analysis is to estimate the number of cases of salmonellosis that would need to be avoided each year through the use of WGS to make its use cost-neutral compared to the costs of using conventional methods. In the previous sections, we have calculated the cost per case of salmonellosis in terms of health care utilisation costs, productivity loss, and premature death, and on this basis, we have estimated the average cost per case of salmonellosis for each of the case study countries. In this section, we compare these estimates to the total cost difference due to the use of WGS versus the use of conventional methods.

The process and results of the breakeven analysis are presented in the following table. For each case study institution, the following aspects are presented:

- The differential cost per sample of using WGS for pathogen identification and surveillance, calculated as difference of the cost per sample using WGS and the costs per sample using conventional methods (presented in detail above);

- The number of *Salmonella* samples analysed per year;

- The total cost difference per year due to the use of WGS, calculated by multiplying the differential costs per sample by the number of samples per year;

- The average cost per *reported* case of salmonellosis in the respective country, as calculated in the previous sub-section;

- The results of the breakeven analysis both in terms of the absolute number of reported cases of salmonellosis and in terms of the percentage of reported cases of *Salmonella* in the geographical jurisdiction of the case study institution that would need to be avoided to make the use of WGS cost-neutral.

Supplementary table S18: Breakeven analysis – Number and percentage of reported cases of salmonellosis that need to be avoided to make the use of WGS cost-neutral

Case study institution (country)	Cost per sample (WGS)	Cost per sample (conven-tional methods)	Differential cost of WGS compared to conventional methods	Number of samples analysed per year	Total cost difference per year due to the use of WGS	Average cost per reported case of salmonellosis in case study country	Number of reported cases of salmonellosis that need to be avoided to break even	Number of cases of salmo-nellosis reported annually*	Percentage of total number of reported cases of salmonellosis that need to be avoided to break even
**PHE (UK)**	€ 124.59	€ 65.46	€ 59.13	10 147	€ 599 992.11	€ 12 400.55	48	8 770	0.6 %
**IZSLER (Italy)**	€ 395.14	€ 91.87	€ 303.27	110	€ 33 360.21	€ 12 124.03	3	** 276	1.0 %
**PHAC (Canada)**	€ 215.36	€ 94.29	€ 121.07	8 273	€ 1 001 622.97	€ 12 174.48	82	7 665	1.1 %
**MDH (US)**	€ 154.51	€ 81.16	€ 73.35	1 010	€ 74 083.50	€ 13 224.76	6	906	0.6 %
**INEI-ANLIS (Argentina)**	€ 154.49	€ 46.61	€ 107.88	128	€ 13 808.64	€ 11 820.61	1	758	0.2 %
**Average**	**€ 208.82**	**€ 75.88**	**€ 132.94**	**3 934**	**€ 344 573.48**	**€ 12 348.89**	**28**	**4 404**	**0.7 %**

Own compilation based on case study results. Sources for the number of infections reported annually in each jurisdiction: ECDC (2018), *The European Surveillance System (TESSy)* (IT); PHE (2018), *Salmonella data 2007 to 2016* (UK); PHAC (2018), *Reported cases from 1924 to 2016 in Canada - Notifiable diseases on-line*; CDC (2015-17), *National Notifiable Infectious Diseases and Conditions in the United States 2015, 2016, 2017* (US); *Laboratory Surveillance System (SIVILA) of the National Health Surveillance System* (Argentina). Note that the averaging rows present averages of the case study figures in each respective column. Notes: * Data provided on cases of salmonellosis refer to the geographical jurisdictions of the institution as indicated in the case study report. Where a case study institution processes samples originating from the whole country (Canada, Argentina), data on salmonellosis refer to the country as a whole. Where a case study institution only processes samples from a specific geographical region within a country, data on salmonellosis refer to this particular region (England, Wales and Northern Ireland in the UK, Emilia-Romagna in Italy, and Maryland in the US). ** Regional data approximated as a population-based proportion of national data, as no regional data was available.

The average cost per reported case of salmonellosis is generally comparable between case studies, as indicated in the table above and discussed in the previous subsection; the key factor determining the absolute number of cases that need to be avoided to break even on costs is therefore the total cost difference per year due to the use of WGS. This figure in turn depends on both the differential cost per sample of using WGS as well as the total number of samples processed, with higher differential costs per sample and higher numbers of samples processed resulting in higher estimates of the number of avoided cases of salmonellosis needed to break even on costs.

The number of cases of salmonellosis that need to be avoided annually to break even on costs ranges from 1 case within INEI-ANLIS' area of jurisdiction (Argentina) to a maximum of 82 cases within PHAC's area of jurisdiction (Canada). While the absolute numbers differ considerably, the number of cases that need to be avoided to break even as a *proportion* of reported cases of infection within each jurisdiction is comparable, lying at 1.1% or less of reported cases for all case studies.

It is important to note that the estimates of 0.2% to 1.1% of cases to be avoided refer to the proportion of *reported* cases and not to the proportion of *total* cases in the community. As discussed above in the introduction to section 5.5, the majority of salmonellosis cases in each country are not recorded in national surveillance statistics. The estimates of 0.2% to 1.1% of cases that would need to be avoided in order to break even on the costs of WGS are therefore conservative figures, with the real proportions of cases that need to be avoided being considerably lower. The reason for this is that reported cases of salmonellosis by definition do not include the estimated 68% of cases at the lowest severity level, as these patients do not present to the health care system. However, it is also relevant to note in this respect that unreported cases are likely to be mostly comprised of 'low cost' cases.

It is also notable that due to the high costs associated with premature death (see the discussion above), the number of deaths that would need to be avoided to break even on WGS lies well below 1 for all case studies, indicating that if even a single death from salmonellosis were avoided each year through the case of WGS in any case study jurisdiction, it would more than break even on costs. For comparison, about 50 deaths from salmonellosis are reported per year in PHE's jurisdiction of England, Wales and Northern Ireland, meaning that one death avoided annually would comprise 2% of all salmonellosis deaths. In fact, given the high cost attached to premature death, avoiding one death every 7.7 years in PHE's jurisdiction would be sufficient to break even on costs; the corresponding values for the other case studies are one avoided death every 5 years in Canada; every 63 years in Maryland (US); every 139 years in Emilia-Romagna (Italy); and every 336 years in Argentina.[14]

3.4. Sensitivity analysis

As previously noted in section 5.5.1.4, the largest single cost component in the average cost per case of salmonellosis is premature death, which comprises approximately 95% of the total average cost. This is due to the large value placed on a human life (EUR 4.6 million), which, even at a low rate of occurrence, overshadows costs of health care or productivity loss. Because the cost of premature death is so dominant in the valuation, it is important to test the robustness of the results against different assumptions regarding the cost or likelihood of premature death.

[14] The number of years here is calculated by dividing the cost of a case at severity level 4 (hospitalised, died) over the total cost difference per year due to the use of WGS.

The first assumption to be tested is the estimated *value of a premature death*. In order to test this assumption, we recalculate the breakeven analysis using the lower estimate for premature death according to ECHA (EUR 3.9 million in EUR 2017) and alternatively using the higher estimate for premature death according to ECHA (EUR 5.5 million in EUR 2017).

The second assumption relates to the *likelihood* of a case of salmonellosis resulting in an outcome of premature death. To test this assumption, we recalculate the breakeven analysis using the outcome distribution used in the USDA's cost of illness model, which assign a lower likelihood to the outcome of premature death. The following table compares the USDA outcome distribution to be used in the sensitivity analysis to the outcome distributions used in the 2011 DG SANCO study (and therefore in our original model).

Supplementary table S19: Outcome distributions – USDA compared to DG SANCO (2011)

	Didn't visit physician, recovered	Visited physician, recovered	Hospitalised, recovered	Hospitalised, died
**DG SANCO (Base)**	68.36 %	28.90 %	2.66 %	0.08 %
**USDA**	90.92 %	7.20 %	1.84 %	0.04 %

Source: DG SANCO/FCC Consortium (2011), *Analysis of the costs and benefits of setting a target for the reduction of Salmonella in breeding pigs – Final report*; USDA Economic Research Service (2014), *Cost estimates of foodborne illnesses*.

As can be seen in the table above, the USDA outcome distribution roughly halves the proportion of cases resulting in premature death relative to the 2011 DG SANCO study, with 0.04% of cases assumed to result in death instead of 0.08%. The USDA also assumes that lower proportions of cases result in hospitalisation or visits to a physician, while a much higher proportion (90.92%) of cases are assumed to recover at home without accessing health care services.

The table below shows the results of the breakeven analysis when recalculated under the three sensitivity scenarios of a lower value of premature death, higher value of premature death, and lower likelihood of premature death.

Supplementary table S20: Sensitivity analysis – base estimate and three sensitivity scenarios

Case study institution (country)	Base estimate using average cost of premature death and likelihood of death according to 2011 SANCO study<br>Cost of premature death: €4.6m<br>Likelihood of death: 0.25%		Revised using lower estimate for value of premature death<br>Cost of premature death: €3.8m<br>Likelihood of death: 0.25%		Revised using higher estimate for value of premature death<br>Cost of premature death: €5.5m<br>Likelihood of death: 0.25%		Revised using a likelihood of death in line with USDA outcome distribution<br>Cost of premature death: €4.6m<br>Likelihood of death: 0.12%	
	**Weighted average cost of reported illness**	**Number of reported cases avoided to break even**	**Weighted average cost of reported illness**	**Number of reported cases avoided to break even**	**Weighted average cost of reported illness**	**Number of reported cases avoided to break even**	**Weighted average cost of reported illness**	**Number of reported cases avoided to break even**
**PHE (UK)**	€ 12 400.55	48	€ 10 320.97	58	€ 14 458.77	41	€ 5 906.29	102
**IZSLER (Italy)**	€ 12 124.03	3	€ 10 048.64	3	€ 14 186.44	2	€ 5 694.49	5
**PHAC (Canada)**	€ 12 174.48	82	€ 10 098.53	99	€ 14 236.33	70	€ 5 734.59	175
**MDH (US)**	€ 13 224.76	6	€ 11 128.17	7	€ 15 265.97	5	€ 6 505.77	11
**INEI-ANLIS (Argentina)**	€ 11 820.61	1	€ 9 750.15	1	€ 13 887.95	1	€ 5 464.40	3

Own compilation based on case study results. Sources: ECHA (2016), *Willingness-to-pay values for various health endpoints associated with chemicals exposure*; USDA Economic Research Service (2014), *Cost estimates of foodborne illnesses*.

As shown in the table above, the sensitivity scenario with the largest impact on the cost of illness and therefore on the number of reported cases of salmonellosis that need to be avoided to break even is the third scenario using the USDA outcome distribution, which reduces the estimated cost of a reported case of salmonellosis by about half, in line with the reduction in the likelihood of death. In contrast, the use of lower and higher estimates for the value of a premature death have a relatively smaller impact on the results.

In terms of the range of results produced by the sensitivity analysis, varying the assumptions related to death has the following results for each case study:

- For PHE, the cost of reported illness ranges from EUR 5 906 to EUR 14 459. The number of reported cases needed to be avoided in order to break even now ranges from 41 (or 0.5% of reported cases) to 102 (equivalent to 1.2% of reported cases);
- For IZSLER, the cost of a reported illness under the sensitivity scenarios ranges from EUR 5 694 to EUR 14 186. The number of reported cases that need to be avoided to break even ranges from 2 (representing 0.9% of reported cases) to 6 (2.1% of reported cases);
- For PHAC, the cost of a reported illness ranges from EUR 5 735 to EUR 14 236. The number of reported cases that would need to be avoided to break even ranges from 70 (representing 0.9% of reported cases) to 175 (representing 2.3% of reported cases);
- For MDH, the cost of a reported illness ranges from EUR 6 506 to EUR 15 266. The number of reported cases to be avoided ranges between 5 (0.5% of reported cases) and 11 (1.3% of reported cases); and
- For INEI-ANLIS, the cost of reported illness ranges from EUR 5 464 to EUR 13 888. This corresponds to a range of between 1 and 3 reported cases that would need to be avoided each year (0.1% to 0.3% of reported cases).

The effect on the overall results, while non-trivial, is relatively modest. Even under the highest impact scenario, i.e. the use of the USDA outcome distribution, the proportion of reported cases that would need to be avoided in each case study jurisdiction in order to break even on the costs of WGS still lies lower than 2.5%.

More importantly, however, the sensitivity analysis does not change the core conclusions of the breakeven analysis relating to the high value of avoiding premature deaths in particular. The estimated value of a premature death, even under the lower estimate, is still very high relative to the additional annual cost of using WGS, so that avoiding just one premature death due to salmonellosis over a period of several years would suffice in all case studies to break even on costs from a public health perspective.

4. Case study reports

4.1. Animal and Plant Health Agency (APHA)

Avian Influenza outbreaks – APHA, UK	
**I. Institution**	
**Name of institution**	The Animal and Plant Health Agency (APHA)
**Type of institution**	Public veterinary institution
**Description**	The Animal and Plant Health Agency (APHA) is an executive agency of the Department for Environment, Food & Rural Affairs (Defra). It also provides services to the Scottish and Welsh Governments, other government departments, and other clients. APHA is responsible for identifying and controlling endemic and exotic diseases and pests in animals, plants and bees, and for surveillance of new and emerging pests and diseases. APHA maintains essential disease investigation and response capability, as well as supporting trade in plants, animals and associated products though certification, audit and inspection, e.g. through import controls of animals, plants, seeds and products of animal origin.<br>APHA conducts scientific research and acts as a national and international reference laboratory for the World Health Organisation (WHO), World Organisation for Animal Health (OIE), and United Nations Food and Agriculture Organisation (FAO), covering many farm animal diseases, including avian influenza. APHA was the EU reference laboratory (EU-RL) for avian influenza until the summer of 2018.
**Location**	Surrey, UK
**II. Activities covered by case study**	
**Activity**	Outbreak investigation[15]
**Reference period**	1 December 2016 – 31 July 2017
**Pathogen(s) covered**	Highly Pathogenic Avian Influenza (HPAI) H5N8
**Outbreak summary**	The outbreak of highly pathogenic avian influenza H5N8 in 2016-2017 occurred in both wild birds and poultry, infecting 13 premises across England and Wales. These included turkey and chicken producers as well as premises involved in gamebird production. The H5N8 infections in poultry are thought to have arisen independently as a result of contact with wild birds, except in the case of a cluster of three infected premises of the same commercial enterprise in Lancashire, where genomic analysis confirmed that secondary infections were likely to have occurred.[c),d)] Note that related H5N8 outbreaks also occurred in continental Europe during this period, but only samples taken in the UK are included in this case study.
**Type of sample**	Primarily isolates where the virus has been cultivated prior to sequencing. However, in some time-sensitive cases the clinical sample is sequenced as-is without growing the virus first, after selecting the 'best' samples in terms of viral content based on the pre-screen PCR.

[15] APHA provided data on two outbreaks: a 2016-2017 outbreak of HPAI H5N8 in wild birds and poultry and a 2017-2018 outbreak of HPAI H5N6 in wild birds only. Data on the outbreak of HPAI N5N6 is presented in Annex II for comparison purposes.

<table>
<tr><td>Region covered by sampling</td><td colspan="3">UK</td></tr>
<tr><td rowspan="2">Number of samples analysed in reference period</td><td>Pathogen</td><td>Samples analysed by conventional methods</td><td>Samples sequenced using WGS</td></tr>
<tr><td>HPAI H5N8</td><td>104 (32 HA, 72 NA)</td><td>26</td></tr>
<tr><td>Conventional methods used as reference for costing</td><td colspan="3">▸ Sanger sequencing (HA and NA analyses, used on 100% of samples)
▸ Manual extraction of RNA using guanidine lysis buffer and silica column purification, generation of target double stranded DNA amplicon (150nt) by RT-PCR using specific primers according to target, BigDye method of labelling and ABI Capillary sequencing</td></tr>
<tr><td>Sample preparation WGS</td><td colspan="3">▸ Manual extraction of RNA using guanidine lysis buffer and silica column purification, ‘shotgun’ generation of double stranded cDNA by RT-PCR of RNA in sample with random hexamers, library generation with Nexterra kit and Illumina WGS</td></tr>
<tr><td>Sequencer used for WGS</td><td colspan="3">▸ Illumina MiSeq</td></tr>
<tr><td>Batch size for WGS analysis</td><td colspan="3">▸ During the outbreak, APHA typically sequenced batch sizes of only 1 or 2 samples due to the time-sensitivity of the results, and this number is the basis for the following cost analysis (outside outbreaks, the typical batch size of amplified isolates would be up to 10 using the MiSeq sequencer).[16]</td></tr>
<tr><td>Reference dataset used for WGS</td><td colspan="3">▸ Reference sequences are chosen from the GISAID database for initial mapping based on assumptions as to the strain identity, then the mapped reads are used in a Blast search of all GenBank sequences to determine an optimal reference sequence for each viral segment. The new reference is then used in the subsequent mapping iterations.</td></tr>
<tr><td>Additional information</td><td colspan="3">▸ WGS is not done on all incoming avian influenza samples at APHA. Sanger sequencing (HA and NA analyses) is still the standard workflow and is required as a confirmatory test.
▸ WGS is currently employed on a routine basis as an additional ‘research’ test, particularly in the initial stages of an outbreak, in cases that show unusual clinical characteristics (e.g. infection of an unexpected species), or in cases where an assessment of the risk to humans is needed. Once the sequence of the index case is known, decisions to sequence additional samples are also made based on epidemiological data.
▸ All incoming avian influenza samples are subject to a pre-screening using real time PCR. From the PCR results, the best samples with the highest virus content are selected for sequencing. The virus would typically be grown further before Sanger sequencing and WGS; however, depending on the time sensitivity of results, it may be sequenced directly from the clinical sample submitted.</td></tr>
</table>

III. Detailed overview of costs of WGS and conventional methods

In the following, all costs are provided on a per-sample basis. Equipment costs are annualised and incorporate the annual maintenance costs as reported by the institution. They are adjusted for the percentage use of the equipment for the listed pathogens samples during the reference period (i.e. if a sequencer was also used for other purposes, this is taken into account). Consumables costs are adjusted for the failure rate (i.e. the percentage of consumables wasted, e.g. due to failed runs). Staff time is

[16] APHA also has an Illumina NextSeq sequencer which can process batch sizes of up to 40 and which has been used by APHA in their capacity as the EURL for avian influenza. However, this sequencer was not used for the UK outbreaks subject to this case study.

provided in terms of the minutes of hands-on staff time per sample, for both professionals and technicians. For the calculation of total costs, staff time is then monetised based on Eurostat data on country-specific labour costs for 2017 (by staff category), plus a 25% surcharge for overheads. For comparison purposes only, we have also provided staff costs monetised based on EU average labour costs. More detailed cost data is provided in Annex I.

a) Costs of using WGS[17]

Sample preparation and sequencing	*Cost type*	*Cost per sample*
	Equipment costs	€ 57.33
	Consumables	€ 830.97
	Other costs	€ 0
	Staff time professionals	*0 minutes*
	Staff time technicians	*210 minutes*
	Staff costs, monetised based on labour cost data for the UK (in brackets: based on labour cost data for the EU as a whole)	€ 87.50 (85.75)
	**Total**	**€ 975.80**

Bioinformatics and other analyses	*Cost type*	*Cost per sample*
	Equipment costs	€ 1.20
	Other costs	€ 0.00
	Staff time professionals	*60 minutes*
	Staff time technicians	*0 minutes*
	Staff costs, based on labour cost data for the UK (for EU)	€ 39.63 (45.13)
	**Total**	**€ 40.83**

b) Costs of conventional methods

Sanger Sequencing (assuming use for 100% of avian influenza samples)	*Cost type*	*Cost per sample*
	Equipment costs	€ 78.55
	Consumables	€ 21.91
	Other costs	€ 0
	Staff time professionals	*60 minutes*
	Staff time technicians	*360 minutes*
	Staff costs, based on labour cost data for the UK (for EU)	€ 189.63 (192.13)
	**Total**	**€ 290.08**

[17] APHA originally provided cost data in pounds sterling. These have been converted to Euro for comparison with the other case studies using the European Central Bank’s yearly average reference exchange rate for the relevant year (i.e. the year of purchase for equipment, or 2017 otherwise).

IV. Costs of using WGS compared to the costs of conventional methods

The following provides a comparison of costs per sample using WGS compared to the costs of conventional methods. See Annex I for more details.

Comparison of equipment costs

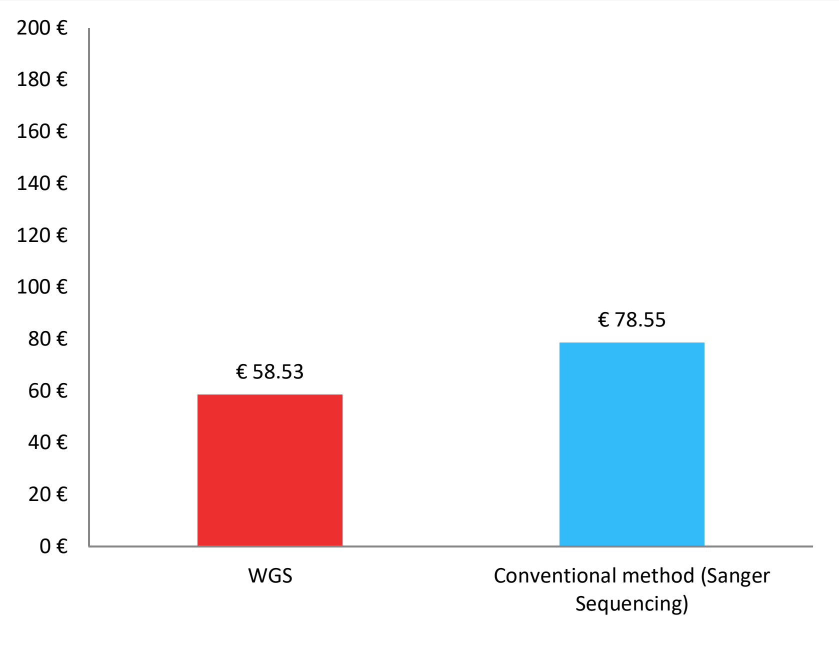

Equipment costs are lower for WGS than for Sanger sequencing (€ 58.53 vs. € 78.55 per sample). One of the factors driving the differential cost is the cost of the sequencer itself: the Illumina MiSeq used for WGS, purchased in 2012 for approximately € 105 000, is lower in price than the ABI Capillary sequencer used for Sanger sequencing, which was purchased in 2009 for approximately € 200 000. Sanger sequencing also makes use of a thermocycler and requires specialised commercial software with a licence that must be renewed annually.

Comparison of costs of consumables

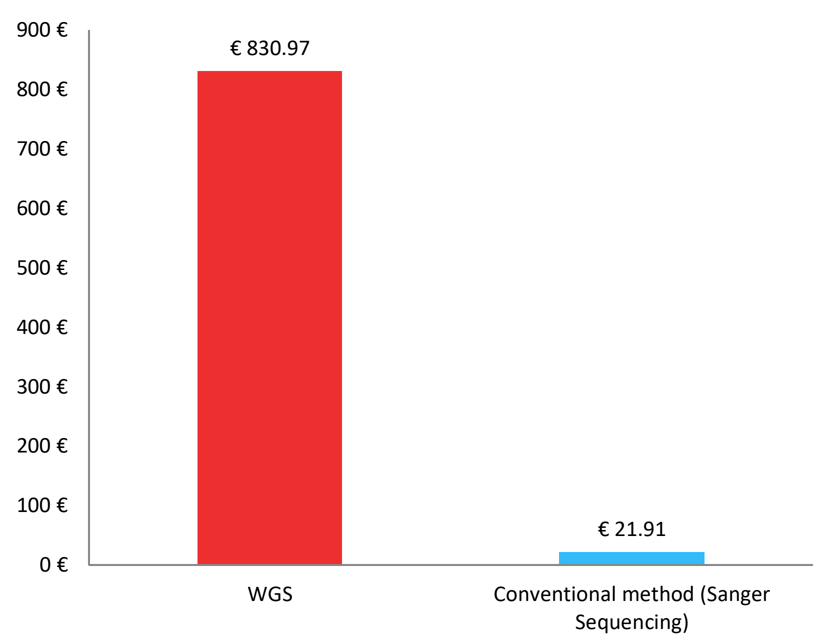

The cost of consumables for WGS is considerably higher than for Sanger sequencing. The large difference in costs is attributable to the cost of the Nextera XT library preparation kit used for WGS and the reagent for the

Illumina run, which costs approximately € 1200 and is used to process only one or two samples at a time in an outbreak situation.[18] In contrast, the consumables used for Sanger sequencing are both cheaper and utilisable for larger batch sizes ranging from 50 to 250.

Comparison of staff time used (in minutes)

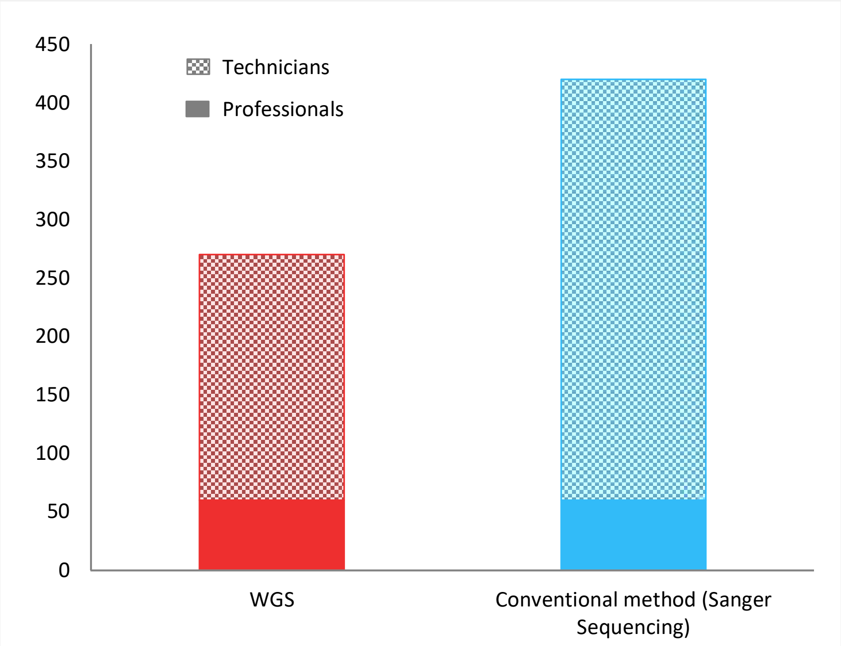

The amount of staff time required for WGS is lower than for Sanger sequencing. Although both methods require the same amount of professional time (60 minutes), Sanger sequencing requires considerably more technician time per sample (360 minutes vs 210 minutes for WGS). All professional staff time required for WGS comes in at the bioinformatics stage; all earlier steps (sample processing, library preparation, sequencing) are handled by technician staff.

Comparison of overall costs

Cost type	*Cost per sample (WGS)*	*Cost per sample (Sanger sequencing)*
Equipment costs	€ 58.53	€ 78.55
Consumables	€ 830.97	€ 21.91
Other costs	€ 0.00	€ 0
Staff time professionals	*60 minutes*	*60 minutes*
Staff time technicians	*210 minutes*	*360 minutes*
Staff costs, based on labour cost data for the UK (for EU)	€ 127.13 (130.88)	€ 189.63 (192.13)
**Total**	**€ 1 016.62**	**€ 290.08**

Summary of differential

A sample analysed with WGS costs considerably more than a sample

[18] APHA indicated that they were able to batch process samples in groups of two more than half of the time during the relevant outbreak. We have therefore assumed an average batch size of 1.6 for the Nextera XT library preparation kit.

costs	**analysed with Sanger sequencing, with a cost difference of € 726.54 per sample** (€ 1 016.62 vs € 290.08). The difference in total per-sample cost is entirely attributable to the large difference in the cost of consumables, which results from a combination of the cost of the Nexterra kit and the small batch size of 1-2 samples.<br>Note that the cost data provided by APHA regarding a second (H5N6) outbreak led to very similar results, with a cost difference of € 720.10 per sample (€ 1 028.86 vs € 308.76). For details, see Annex II.

V. Effects of using WGS results

a) Turnaround time. Turnaround time is defined as the usual number of days of work from receipt and opening of an incoming sample until the reporting of results. Turnaround time does not include weekends and holidays, except in case that work has been conducted on these days, e.g. for a sequencing run or other analyses.

Turnaround time	The turnaround time for the analysis of an avian influenza sample is:<br>• Using WGS, a minimum of 3-5 days of work to sequence in a case where no virus amplification is needed.<br>• Using Sanger sequencing, a minimum of 1-2 days of work in a case where no virus amplification is needed.<br>APHA indicated that the difference in turnaround time between Sanger sequencing and WGS arises due to machine processing time and especially the time required for analysis, as WGS results are vastly more complex and require special software to interpret. However, it indicated that the turnaround time for Sanger sequencing depends on making an accurate estimate as to the correct primers to use, and reported that the turnaround time for Sanger sequencing could be longer if the initially-selected primers are incorrect and new primers need to be designed or ordered.<br>In cases where virus amplification (i.e. prior growth of the virus) is needed, turnaround time is higher, depending on how quickly the virus grows. The process of growing the virus adds an additional 4-6 days (on average: 4).

b) Positive effects of using WGS for pathogen identification and surveillance during the reference period

Note that or this case study, APHA provided data on two outbreaks: for the above described H5N8 outbreak (outbreak 1) and for a subsequent H5N6 outbreak (Annex II). The positive effects of using WGS described below were experienced for both outbreaks, except where indicated otherwise.

Sampling and sampling strategies	• APHA indicated that it saw very significant positive effects with respect to the simplification of the type of samples needed, noting that WGS was able to reduce the pre-processing required for the sample in cases where no viral amplification was necessary. This results in time savings of approximately 2 work days for generating run-ready samples. However, APHA noted that viral amplification is needed more often for WGS.<br>• APHA noted that each outbreak of HPAI was different, and that the consideration of positive effects of WGS therefore also different between cases. During the H5N8 outbreak, for example, no further effects on sampling and sampling strategies were noted, as APHA indicated that the sampling is determined by clinical findings and epidemiology, independent from whether WGS or Sanger sequencing is used. For the H5N6 outbreak, however, which was smaller and limited to isolated outbreaks in wild birds, APHA indicated that there had also been a reduction in the number of samples needed, simplification in sample storage/transport, and a reduction in the overall costs of sampling. It indicated that this was because WGS analysis allowed for confirmation that the separate UK isolates were all highly similar to viruses present in Continental Europe and were not direct introductions from South-East Asia.

Analytical results and processes	▸ APHA considered that using WGS had led to very significant positive effects on the accuracy, sensitivity, and specificity of results. In particular, it commented that WGS produced many reads of a sequence, resulting in higher accuracy and greater statistical confidence in the outputs, and also allowed viral genome-spanning information to be rapidly obtained regarding the genotype, pathotype, mutations, etc.<br>▸ APHA also noted that WGS is adaptable to high-throughput and automated pipelines. For example, APHA noted that a robot can be used for the library preparation stages (although this is not currently done at APHA).<br>▸ The institution indicated that another positive effect of WGS is that no prior knowledge of the target sequence is required, so no assumptions need to be made regarding the primers needed for WGS sample preparation. In contrast, if the primers available for Sanger sequencing fail to produce an amplicon, considerable time can be needed to design, order and receive new primers.<br>▸ During the H5N6 outbreak, APHA considered that using WGS had a significant positive effect on the simplification of laboratory work flows. This is in contrast to the situation reported in the H5N8 outbreak, where APHA considered that WGS had only a minor effect in this area.<br>▸ APHA considered that during the H5N6 outbreak there had been slightly more significant effects of using WGS concerning a reduction in the consumables and staff time required for the analysis than during the H5N8 outbreak, although this was not reflected in the cost data. While similar numbers of samples underwent WGS, in the case of the H5N6 outbreak, this reduced the need for additional sample analysis.
Outbreak identification and response	▸ Positive effects of using WGS were reported with respect to improved information on outbreak epidemiology, improved information for imposing additional control or biosecurity measures, and improved detection that outbreaks are related. APHA indicated that the information provided by WGS was already changing outbreak response in terms of being able to better assess the public health risk, for example by revealing the presence of mutations for mammalian host adaptation and the possible emergence of reassortant strains. It added that WGS also allowed for useful supporting information to be disseminated during outbreaks.<br>▸ In the H5N8 outbreak, APHA indicated that there had been a very significant positive impact of using WGS on the earlier detection of an initial outbreak, especially for the index case. APHA indicated that the information gained from WGS allows them to better assess whether the virus sampled poses a risk of transmission to humans. This effect was less pronounced for the H5N6 outbreak, once it was determined that the H5N6 outbreak strain was distinct from the H5N6 lineage associated with human infection in South-East Asia. APHA commented that WGS sometimes allows for the earlier confirmation of an outbreak and noted that WGS is still not an accredited method in the UK, but that results are given unofficially and inform the interpretation of all results.<br>▸ In the H5N8 outbreak, fewer positive effects were observed with respect to a reduction in the duration of the outbreak, reduction in the overall costs for outbreak identification and response, and reduction in the disease burden for livestock and humans. These effects were considered to have been comparatively larger in the H5N6 outbreak.
Research and methods applied	▸ With respect to the effects on research and methods applied, APHA reported that there had been positive effects regarding the understanding of disease transmission, an improvement in epidemiological methods, and the development of better diagnostic tests, although it assessed these benefits to have been higher in the case of the H5N6 outbreak than in the case of the H5N8 outbreak. Regarding the use of diagnostic tests, APHA indicated that the information gained from WGS helped determine which

	conventional tests to use later on in the outbreak.<br>- APHA indicated that WGS provides a lot of added value in dealing with the influenza virus, given the amount of variation observed. WGS can be used to identify novel viruses, reassortants, and mixed infections (e.g. mixed avian influenza subtypes or other pathogens) which would otherwise be missed using conventional methods. WGS also provides information on the host of origin.<br>- With respect to the H5N6 outbreak, APHA indicated that the use of WGS had allowed them to infer zoonotic risk according to mammalian adaptation signatures and to determine the likelihood or not of pre-existing immunity.
Effects on wider society	- APHA indicated that in the H5N8 outbreak, positive effects of using WGS could be observed with respect to a reduction in the negative effects of outbreaks for the livestock industry, for tourism, for trade, and for the wider society. Trade in particular was emphasised as an area where APHA observed positive effects from using WGS, given that HPAI had been discovered in domestic poultry. In the H5N6 outbreak, in contrast, APHA observed less significant impacts on all these domains, as the outbreak had remained confined to wild birds and did not infect poultry.
c) Negative effects of using WGS	
Negative effects of using WGS	None identified/reported other than the higher cost, although APHA indicated that from their perspective, the cost-benefit ratio of using WGS in terms of the information obtained was more favourable.
**VI. Outlook**	
Balance of costs and benefits achieved	- In general, APHA expected the balance of costs and benefits to improve. It commented that as WGS becomes more mainstream, there will be an economies of scale effect with more samples sequenced and individual run costs decreasing. Technological advances (e.g. related to the MinION) are also expected to result in further cost reductions (see below) as well as the ability to sequence clinical samples directly and to potentially sequence RNA directly.
Potential for cost reductions of using WGS for pathogen identification and surveillance in the future (through e.g. techno-logical advances)	- APHA expected that there will be further cost reductions in using WGS for pathogen identification and surveillance as the technology becomes more mainstream. APHA also indicated that they are currently looking at ways of optimising costs by batching samples for analysis or sequencing directly from clinical samples, thereby avoiding the virus amplification step and saving time and money. In this respect, they consider that advances in direct RNA sequencing methods and/or other technologies such as the MinION will result in considerable time and cost savings.
Future opportunities and challenges	- APHA considered that the cross-pathogen potential of WGS will become a reality, including across different networks and contexts. Nevertheless, APHA considered that there were unlikely to be cost reductions resulting from the cross-pathogen potential of WGS in the influenza field. However, it did see considerable future potential in the influenza field for coordination between the veterinary and public health sectors under a One Health approach.<br>- APHA commented that the bioinformatics and analysis aspect of WGS formed a sort of 'bottleneck', given that it currently relies on 'freeware' and the coding ability of individuals who have a rare combination of IT skills and an understanding of virology. In this respect, it considered that the COMPARE project was filling a significant gap.<br>- APHA commented that although the knowledge gained from WGS was

	often applied in decision-making and outbreak management, it does not easily fit into the strict quality confines of statutory testing and considered that this posed a large hurdle to making the technology 'mainstream'.

VII. Key sources/references

Questionnaire	Questionnaire completed by APHA
Preparatory phone interview	a) Background information and description of activities
Case study visit and follow up	b) Additional data and clarifications provided
Scientific literature	c) Animal and Plant Health Agency (APHA). (2017). *National epidemiology report - Highly Pathogenic Avian Influenza H5N8 - Annex 1: Three additional infected small-holder premises - April to May 2017*.<br>d) Animal and Plant Health Agency (APHA). (2017). *National epidemiology report - Highly Pathogenic Avian Influenza H5N8: December 2016 to March 2017*.<br>e) Poen, M. J., Verhagen, J. H., Manvell, R. J., Brown, I., Bestebroer, T., van der Vliet, S., … Fouchier, R. A. M. (3016). Lack of virological and serological evidence for continued circulation of highly pathogenic avian influenza H5N8 virus in wild birds in the Netherlands, 14 November 2014 to 31 January 2016. *Eurosurveillance*, 21(38).<br>h) Department for Environment Food and Rural Affairs (Defra). (2018). *Rapid Risk Assessment on the finding of H5N6 HPAI in wild birds in England and Wales.*<br>i) Department for Environment Food and Rural Affairs (Defra). (2018). *Rapid Risk Assessment on the finding of H5N6 HPAI in wild birds in Dorset.*<br>j) Department for Environment Food and Rural Affairs (Defra), Animal and Plant Health Agency (APHA), and Veterinary & Science Policy Advice Team - International Disease Monitoring. (2018). *Situation Assessment #4: Update on H5N6 HPAI in UK/Europe and H5N8 HPAI in Europe/Western Russia - 9 July 2018.*<br>k) Department for Environment Food and Rural Affairs (Defra), Animal and Plant Health Agency (APHA), and Veterinary & Science Policy Advice Team - International Disease Monitoring. (2018). *Situation Assessment #3: Update on H5N6 HPAI in UK/Europe and H5N8 HPAI in Europe - 4 April 2018.*<br>l) Department for Environment Food and Rural Affairs (Defra), Animal and Plant Health Agency (APHA), and Veterinary & Science Policy Advice Team - International Disease Monitoring. (2018). *Situation Assessment #2: Findings of H5N6 HPAI in wild birds in UK / Ireland and LPAI in poultry in France - 14 February 2018.*<br>m) Department for Environment Food and Rural Affairs (Defra), Animal and Plant Health Agency (APHA), and Veterinary & Science Policy Advice Team - International Disease Monitoring. (2018). *Situation Assessment: Findings of H5N6 HPAI in wild birds - 30 January 2018.*
Other sources	f) APHA, Annual Report and Accounts 2016/17<br>g) APHA website https://www.gov.uk/government/organisations/animal-and-plant-health-agency

4.2. Friedrich-Loeffler-Institut (FLI)

Avian Influenza outbreak – FLI, Germany	
**I. Institution**	
**Name of institution**	Friedrich-Loeffler-Institut (FLI)
**Type of institution**	Public veterinary institution
**Description**	The Friedrich-Loeffer-Institut (FLI) is the National Institute for Animal Health in Germany. It is a federal research institute and independent higher federal authority under the Federal Ministry for Food and Agriculture. Its work aims at the prevention of diseases, the improvement of animal welfare and the production of high quality animal-based foodstuffs. The institute performs epidemiological investigations during outbreaks of animal diseases. It also prepares risk assessments on various infectious diseases of farm animals.<br>FLI hosts the National Reference Laboratory for Avian Influenza, which conducts application-oriented research in the field of avian influenza virus diagnostics, epidemiology and pathogenesis. It is also active within the EU-RL network for Avian Influenza. As a reference laboratory of the World Organisation for Animal Health (OIE) and of the Food and Agriculture Organization (FAO) of the United Nations, the laboratory provides advice and diagnostic assistance to countries outside Europe.<br>FLI has a laboratory for WGS and Microarray Diagnostics. The main task of the laboratory for WGS and microarray diagnostics is full-length DNA or RNA virus genome sequencing. Beyond the sequencing activities, establishing new technical equipment, molecular biological methods, and implementing new ways for data analyses are among FLI's focus areas.[j)]
**Location**	Greifswald, Mecklenburg-Vorpommern, Germany
**II. Activities covered by case study**	
**Activity**	Outbreak investigation
**Reference period**	24/12/2016 – 28/03/2017
**Pathogen(s) covered**	Avian Influenza (AI)
**Outbreak summary**	In 2016/2017 a regional outbreak of notifiable H5 Highly Pathogenic Avian Influenza (HPAI) occurred in Lower Saxony in domestic poultry farms, principally of avian influenza subtype H5N8 with some infections of subtype H5N5. Several turkey fattening farms were affected. This was the largest outbreak in one area ever recorded in Germany, with about 30 farms affected. Culling and cleaning procedures, commercial restrictions and compensation led to high costs (estimated at EUR 500 000 per farm, depending on the number of hold poultry).<br>Epidemiological connections were initially unknown to authorities, which therefore sought the help of FLI. Analysis using whole-genome sequencing was able to indicate that transmission occurred not only through wild birds but also through secondary infection between farms, exposing gaps in biosecurity measures in addition to other potential risk factors.[c)]<br>The regional outbreak in Lower Saxony was part of a larger outbreak of HPAI across Germany, with more than 1 150 cases of H5Nx infection reported in wild birds and 107 outbreaks among birds kept in captivity (including both poultry and zoos) between November 8, 2016 and September 30, 2017, resulting in the death or slaughtering of approximately 1.2 million birds. Estimated direct economic losses of the total outbreak across Germany were about EUR 17 million.[f)]

**Type of sample**	Isolates		
**Region covered by sampling**	Lower Saxony, Germany		
**Number of samples analysed in reference period**	*Pathogen*	*Samples analysed by conventional methods*	*Samples sequenced using WGS*
	H5 Highly Pathogenic Avian Influenza Virus	The cost calculation is based on previous experiences with the listed conventional method, assuming the same number of samples as with WGS	30
**Conventional method used as reference for costing**	▸ Sanger sequencing of complete genomes<br>▸ Manual sample preparation<br>▸ 13 PCR products per sample, 2-fold coverage		
**Sample preparation WGS**	▸ Manual sample preparation		
**Sequencer used for WGS**	▸ Ion Torrent PGM bundle		
**Batch size for WGS analysis**	▸ The data provided is based on batches of 6 samples per sequencing run.		
**Reference dataset used for WGS**	▸ FLI maintains its own reference dataset for avian influenza, which is manually created and curated. The dataset is updated via public databases on a regular basis. Data are also shared between reference laboratories prior to publication.		
**Additional information**	▸ Activities covered by this case study include analyses of known avian influenza samples within the context of the relevant outbreak.<br>▸ Note that FLI is a research institution handling a large number of different pathogens of varying virulence. To avoid cross-contaminations, very strict laboratory procedures are applied, as was emphasised by FLI. This may lead to increased staff time and consumable costs for specific analyses. For example, when handling samples, gloves are changed after each analytical step.		

III. Detailed overview of costs of WGS and conventional methods

In the following, all costs are provided on a per-sample basis. Equipment costs are annualised and incorporate the annual maintenance costs as reported by the institution. They are adjusted for the percentage use of the equipment for the listed pathogens samples during the reference period (i.e. if a sequencer was also used for other purposes, this is taken into account). Consumables costs are adjusted for the failure rate (i.e. the percentage of consumables wasted, e.g. due to failed runs). Staff time is provided in terms of the minutes of hands-on staff time per sample, for both professionals and technicians. For the calculation of total costs, staff time is then monetised based on Eurostat data on country-specific labour costs for 2017 (by staff category), plus a 25% surcharge for overheads. For comparison purposes only, we have also provided staff costs monetised based on EU average labour costs.

More detailed cost data is provided in Annex I.

a) Costs of using WGS

Sample preparation and sequencing	*Cost type*	*Cost per sample*
	Equipment costs	€ 198.79
	Consumables	€ 254.88
	Other costs	€ 0

	Staff time professionals	*18 minutes*
	Staff time technicians	*135 minutes*
	Staff costs, monetised based on labour cost data for Germany (in brackets: based on labour cost data for the EU as a whole)	€ 76.16 (68.66)
	**Total**	**€ 529.83**
Bioinformatics and other analyses	*Cost type*	*Cost per sample*
	Equipment costs	€ 11.92
	Other costs	€ 0
	Staff time professionals	*30 minutes*
	Staff time technicians	*0 minute*
	Staff costs, based on labour cost data for Germany (for EU)	€ 26.63 (22.56)
	**Total**	**€ 38.54**
b) Costs of conventional method (based on previous experiences with the listed method)		
Sanger Sequencing of an entire genome (assuming a use for 100% of avian influenza samples)	*Cost type*	*Cost per sample*
	Equipment costs	€ 137.35
	Consumables	€ 360.88
	Other costs	€ 0
	Staff time professionals	*260 minutes*
	Staff time technicians	*240 minutes*
	Staff costs, based on labour cost data for Germany (for EU)	€ 337.75 (293.54)
	**Total**	**€ 835.98**

**IV. Costs of using WGS compared to the costs of conventional methods**	
The following provides a comparison of costs per sample using WGS compared to the costs of conventional methods. See Annex I for more details.	
Comparison of equipment costs	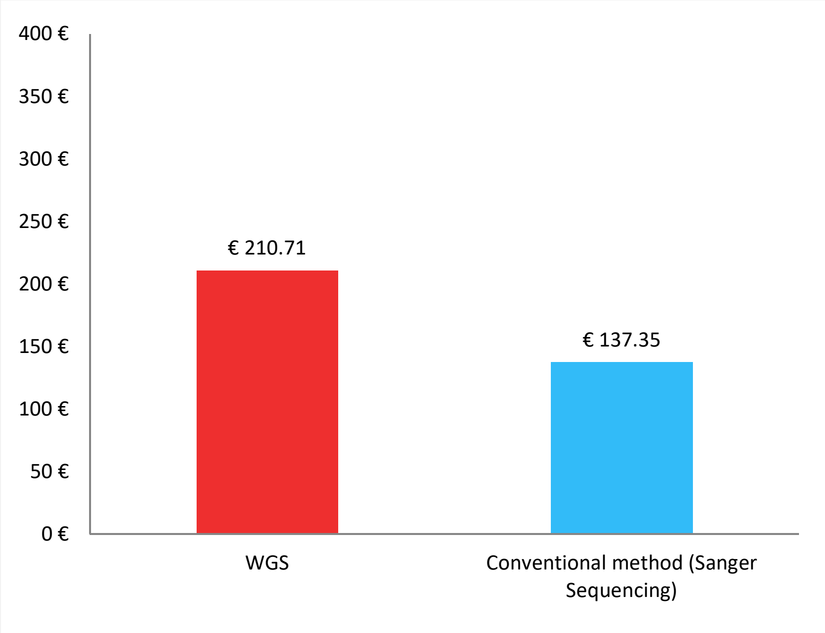<br>Equipment costs are significantly higher for WGS than for Sanger sequencing of an entire genome (€ 210.71 vs. € 137.35 per sample), mostly due to the purchase and maintenance costs of the IonTorrent sequencer itself.
Comparison of costs of consumables	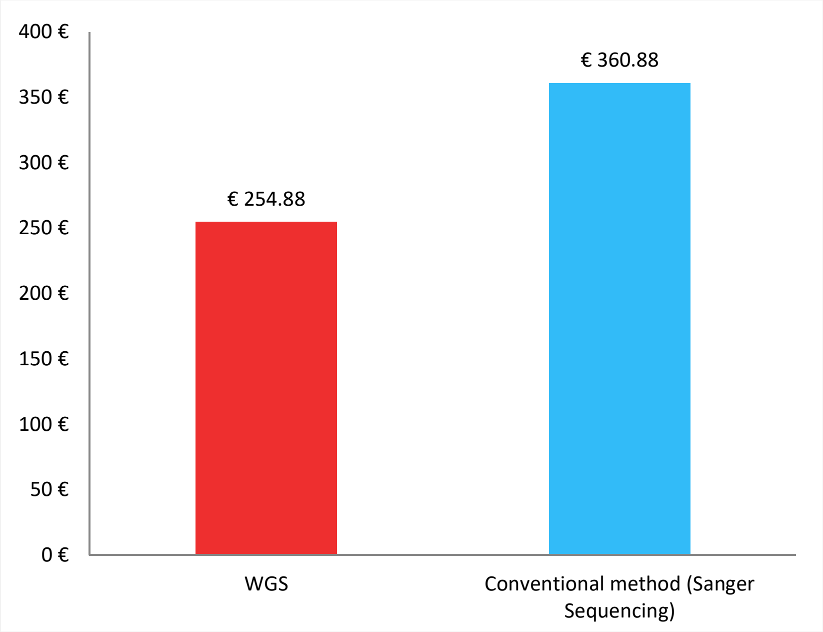<br>In contrast, costs of consumables for WGS are lower than for Sanger sequencing of an entire genome (€ 254.88 vs. € 360.88 per sample). This is mostly attributable to the cost of consumables used for library preparation and sequencing, which are higher for Sanger sequencing of an entire genome.
Comparison of staff time used	

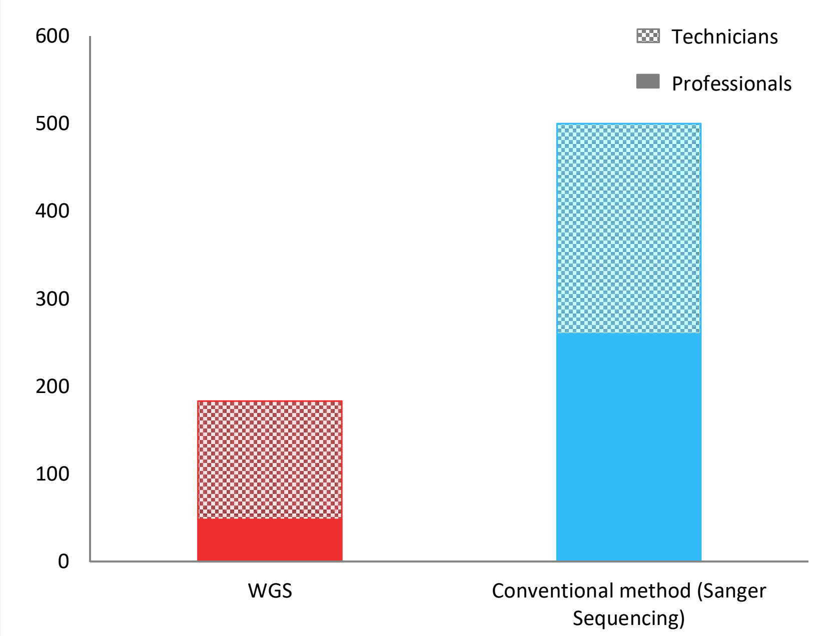

The amount of staff time needed for WGS is considerably lower than for Sanger sequencing of an entire genome; however, comparatively more professional time is required for WGS, especially at the bioinformatics stage, which is exclusively conducted by professionals. Nevertheless, after monetising staff time, staff costs per sample are still more than three times higher for Sanger sequencing of an entire genome (see table below).

Comparison of overall costs	*Cost type*	*Cost per sample (WGS)*	*Cost per sample (Sanger Sequencing)*
	Equipment costs	€ 210.71	€ 137.35
	Consumables	€ 254.88	€ 360.88
	Other costs	€ 0	€ 0
	Staff time professionals	*48 minutes*	*260 minutes*
	Staff time technicians	*135 minutes*	*240 minutes*
	Staff costs, based on labour cost data for Germany (for EU)	€ 102.79 (91.23)	€ 337.75 (293.54)
	**Total**	**€ 568.37**	**€ 835.98**

Summary of differential costs	**A sample analysed with the use of WGS costs less than the cost of analysis with the conventional method (Sanger sequencing of an entire genome), with a cost difference of € 267.61 per sample** (€ 568.37 vs € 835.98). As indicated in the figures above, major differences in costs were found to exist in all cost categories, but especially regarding staff time.

**V. Effects of using WGS results**	
a) Turnaround time. Turnaround time is defined as the usual number of days of work from receipt and opening of an incoming sample until the reporting of results. Turnaround time does not include weekends and holidays, except in case that work has been conducted on these days, e.g. for a sequencing run or other analyses.	
Turnaround time	The turnaround time for the analysis of an avian influenza sample is:<br>▸ 4 days of work using WGS (sequencing of the full genome), compared to<br>▸ 8 days of work for pathogen whole genome sequencing using Sanger Sequencing.<br>While conventional methods are therefore able to provide a fast identification of high vs. low pathogenicity of a given AI sample, WGS provides additional information on virus reassortment as well as the phylogenetic relationships. (FLI also provided the hypothetical turnaround time, if Sanger Sequencing was used to only analyse the HA segment for HPAI LPAI discrimination: This would take 2 days of work.)
b) Positive effects of using WGS for pathogen identification and surveillance during the reference period	
Sampling and sampling strategies	▸ Little or no positive effects on sampling and sampling strategies are expected from FLI's perspective despite the fact that less material is needed in terms of starting material from the extracted nucleic acids.
Analytical results and processes	▸ Overall FLI sees little evidence so far of positive effects of WGS on analytical results and processes (e.g. on the simplification of laboratory flows or consumables needed for the analysis), although it did report a clear reduction in the necessary staff time, especially when comparing WGS with Sanger sequencing of complete genomes.<br>▸ The institution nonetheless reported very significant positive effects of WGS on the level of detail of results produced, as well as moderately positive effects on the sensitivity of the results and reduction of overall costs for the analysis.
Outbreak identification and response	▸ Significant improvements were reported regarding the ability to detect that outbreaks are related, improved information on outbreak epidemiology (e.g. the ability to link cases to the source of infection), and a reduction in the number of secondary outbreaks. In particular, the use of WGS was able to confirm that transmission in the relevant outbreak occurred not just through wild birds but also through secondary infections between farms, highlighting potential gaps in biosecurity measures.[c),d)] Accordingly, FLI also identified positive effects regarding improved information for imposing additional control/biosecurity measures, as well as a reduction in the duration of outbreaks.<br>▸ FLI indicated that the genetic data provided a lot of information (on waves, clusters, and possible sources) and therefore provided hints towards certain transmission routes, allowing for some possibilities to be clearly ruled out. For example, in the present case study, FLI indicated that there were two consecutive outbreaks on one farm, raising questions regarding the effectiveness of the cleaning measures performed after the first outbreak; however, WGS analysis showed that the second outbreak on the same farm was caused by a later strain of the virus and was therefore the result of a separate introduction.<br>▸ Fewer benefits of WGS were reported with respect to earlier detection of the initial outbreak, given that FLI worked with samples that had already been positively identified through conventional methods. Fewer benefits were also noted with respect to a reduction in the disease burden and reduction in overall costs for outbreak identification and response.
Research and methods	▸ Regarding the positive effects on research and methods applied, FLI

applied	reported very significant improvement in the understanding of disease transmission and in epidemiological methods. FLI indicated that the same results could not be achieved with Sanger sequencing due to the level of sensitivity required.
Effects on wider society	▶ The institution considered that the use of WGS leads to positive effects for the wider society especially in relation to a reduction in the costs of outbreak(s), including through the reduction of compensation payments, and also a reduction in negative effects of the outbreak on trade (although only to a moderate extent).
c) Negative effects of using WGS	
Negative effects of using WGS	There are concerns from the industry perspective that WGS can uncover suboptimal practices e.g. in trade, biosecurity, diagnostics etc. In the present case study, for example, WGS was able to identify substantial gaps in farm biosecurity measures that contributed to the farm-to-farm transmission of avian influenza within Lower Saxony.[c),f)] Such findings could contribute e.g. to lower compensation payments or other questions of liability where secondary infections result in large economic losses. FLI indicated that to avoid a reduction in cooperation, the use of very detailed techniques and data analyses needs a proactive and careful communication strategy.
**VI. Outlook**	
Balance of costs and benefits achieved	▶ The efforts currently required for WGS analysis as well as the associated costs (especially equipment) are high, but it is expected that the costs of sequencing and analysis will come down, driven by the demand for sequencing. This is already the case to some extent (e.g. the cost of sequencers have already come down significantly) and the balance of costs and benefits is expected to improve in the mid- to long term.
Potential for cost reductions	▶ FLI is in the process of introducing further automation for sample preparation, which is expected to lead to a substantial reduction in hands-on staff time.<br>▶ In the study of the Influenza outbreak considered here, the only significant cost reduction could have been achieved by higher multiplexing in the sequencing run. This, however, would have resulted in extended turnaround times, and was therefore in this case avoided. With regard to cross-pathogen detection, FLI indicated that sample preparation was the most expensive stage and that therefore further cost reductions at the lab level could be possible with the use of different methods. This is however not feasible at the moment.<br>▶ Using such new methods, the costs of consumables would also be expected to decrease.
Future opportunities and challenges	▶ In the veterinary field (with a strong focus on notifiable diseases, which are well-known and for which PCR tests are available) WGS would only be used as a first step in rare cases where a diagnosis is unclear or where a novel or unknown pathogen is concerned, as WGS is much more expensive overall. Especially in case of an outbreak, under the current cost conditions, PCR would be the method of choice for initial identification of the pathogen.<br>▶ In the institution's perspective, the most relevant use of the cross-pathogen potential of WGS at this stage is human diagnostics in a clinical context, often through a national reference centre. For instance, FLI often receives requests regarding cases in the human field, where a hospital has an urgent case in which the pathogen could not be identified after running 30-60 PCRs (e.g. for cases of Encephalitis). These cases show most clearly the benefits of WGS and may be more economical to investigate with WGS

	rather than with multiple disease specific tests. The difficult nature of WGS for diagnostics nonetheless remains a challenge. It is expected to take at least 5-10 years before it is so simple that it can be used broadly (similarly to the past development regarding PCR diagnostics).<br>▶ The institution considered that metagenomics is still more of a niche topic. The analysis of an unknown pathogen for a metagenomic analysis would require more preparation, and more sequencing runs with fewer samples per run and more depth.<br>▶ Data accuracy is an area of concern with respect to the use of public databases, where there is a need for greater curation and validation by specialists. Data security will also be an emerging concern that will slow down the pace of analysis.

VII. Key sources/references

Cost questionnaire	Cost questionnaire completed by FLI
Preparatory phone interview	a) Background information and description of activities
Case study visit and follow up	b) Additional data and clarifications provided
Scientific literature	c) Conraths, F. J. (2017). Making worst case scenarios real: The introduction of highly pathogenic avian influenza of subtype H5N8 led to the largest fowl plague outbreak ever recorded in Germany. *Lohmann Information*, 51(1), 36–41.<br>d) Conraths, F. J., et al. (2017). *Epidemiologie des aktuellen Geflügelpestgeschehens in Deutschland* [Epidemiology of the current incidence of avian influenza in Germany], presentation given at the meeting of the Gesellschaft der Förderer und Freunde für Geflügel- und Kleintierforschung e.V. at the Institut für Tierschutz und Tierhaltung in Celle on 3 May 2017.<br>e) Friedrich-Loeffler-Institut. (2017). *Qualitative Risikobewertung zur Einschleppung sowie zum Auftreten von hochpathogenem aviären Influenzavirus H5 in Hausgeflügelbestände in Deutschland*.<br>f) Globig, A., et al (2018). Highly Pathogenic Avian Influenza H5N8 Clade 2.3.4.4b in Germany in 2016/2017. *Frontiers in Veterinary Science*, 4(January), 2–9. http://doi.org/10.3389/fvets.2017.00240<br>g) Grund, C., et al. (2018). A novel European H5N8 influenza A virus has increased virulence in ducks but low zoonotic potential. *Emerging Microbes and Infections*, 7(1), 1–14. http://doi.org/10.1038/s41426-018-0130-1<br>h) Pohlmann, A., et al. (2018). Swarm incursions of reassortants of highly pathogenic avian influenza virus strains H5N8 and H5N5, clade 2.3.4.4b, Germany, winter 2016/17. *Scientific Reports*, 8(1), 8–13. http://doi.org/10.1038/s41598-017-16936-8<br>i) Pohlmann, A., et al (2017). Outbreaks among Wild Birds and Domestic Poulty Caused by Reassorted Influenza A(H5N8) Clade 2.3.4.4 Viruses, Germany, 2016. *Emerging Infectious Diseases*, 23(4), 633–636. http://doi.org/http://dx.doi.org/10.3201/eid2304.161949
Other	j) FLI website, https://www.fli.de/en

4.3. Erasmus Medical Centre (EMC)

<table>
<tr><th colspan="4">Influenza surveillance – Erasmus MC, NL</th></tr>
<tr><td colspan="4">I. Institution</td></tr>
<tr><td>Name of institution</td><td colspan="3">Erasmus University Medical Centre (Erasmus MC)</td></tr>
<tr><td>Type of institution</td><td colspan="3">University hospital</td></tr>
<tr><td>Description [c)]</td><td colspan="3">Erasmus MC is the largest university hospital in the Netherlands. It conducts research in various fields, studying fundamental and clinical domains as well as public health and prevention. The Department of Viroscience at Erasmus MC has expertise ranging from basic virology to clinical virology, connecting medical and veterinary health, public health and ecology.
The Department of Viroscience at Erasmus MC is the national reference centre for influenza and emerging infections in the Netherlands, as well as a WHO Collaborating Centre on viral infections.</td></tr>
<tr><td>Location</td><td colspan="3">Rotterdam, NL</td></tr>
<tr><td colspan="4">II. Surveillance activities covered by case study</td></tr>
<tr><td>Activity</td><td colspan="3">Routine laboratory surveillance</td></tr>
<tr><td>Reference period</td><td colspan="3">12/2018 – 04/2019</td></tr>
<tr><td>Pathogen(s) covered</td><td colspan="3">Influenza virus A & B</td></tr>
<tr><td>Summary of routine surveillance activities using WGS</td><td colspan="3">Nanopore sequencing with the use of the GridION platform, a third generation sequencing approach, was introduced for routine surveillance of influenza at Erasmus MC at the beginning of the influenza virus season in November 2018. Nanopore sequencing largely replaced conventional virus culture and characterization plus Sanger sequencing for the 2018/2019 influenza virus season.</td></tr>
<tr><td>Type of sample</td><td colspan="3">Clinical samples</td></tr>
<tr><td>Region covered by laboratory surveillance</td><td colspan="3">The Netherlands</td></tr>
<tr><td rowspan="2">Number of samples analysed in reference period</td><td>Pathogen</td><td>Samples analysed by conventional methods</td><td>Samples sequenced using WGS</td></tr>
<tr><td>Influenza A (H1N1, H3N2) and B</td><td>The cost calculation is based on previous experiences with the listed conventional methods, assuming the same number of samples as with WGS</td><td>630</td></tr>
<tr><td>Conventional methods used as reference for costing</td><td colspan="3">- Average for an influenza season: Real Time PCR (N= 630; 100%), virus isolation for 108 samples with high virus load (17%), phenotyping of virus isolates - Hemagglutination inhibition (34 samples, 5%) and/or Virus neutralization (20 samples, 3%) and/or NA-Star (25 samples, 4%) - and Sanger Sequencing of a representative subset (27 samples, 4%), The numbers listed here are the averages over four recent influenza seasons (2014-2018).</td></tr>
<tr><td>Sample preparation WGS</td><td colspan="3">- Manual sample and library preparation</td></tr>
<tr><td>Sequencer used for WGS</td><td colspan="3">- Nanopore GridION</td></tr>
<tr><td>Batch size for WGS analysis</td><td colspan="3">- The typical batch size increased over the flu season from 10 to 40, with an average batch size of 30 samples</td></tr>
</table>

**Reference dataset used for WGS**	‣ Erasmus MC does not maintain its own internal reference database, but downloads data as needed from public databases (notably GISAID). It uses the new vaccine strains as reference strains each season.
**Additional information**	‣ Originally, the National Influenza Centre attempted to isolate the influenza virus from influenza cases and then characterised these viruses by hemagglutination inhibition (HI) assay or focus-reduction assay (FRA) and NA-star assay. Sanger sequencing was then used for a subset of representative viruses. In the last season, this process was reversed; samples were first subjected to WGS using the GridION and the virus was isolated and characterised for a subset of representative viruses.<br>‣ Consequently, for the 2018-2019 flu season, regular conventional testing was carried out in parallel to WGS, although at a lower intensity than in previous flu seasons. In the 2018-2019 flu season, 50 samples (8%) were subject to virus isolation, 15 (2%) to Hemagglutination inhibition, 8 (1%) to virus neutralisation, and 10 (2%) to NA-star. These methods have been costed into the WGS workflow below as 'supplementary conventional tests'.

III. Detailed overview of costs of WGS and conventional methods

In the following, all costs are provided on a per-sample basis. Equipment costs are annualised and incorporate the annual maintenance costs as reported by the institution. They are adjusted for the percentage use of the equipment for the listed pathogens samples during the reference period (i.e. if a sequencer was also used for other purposes, this is taken into account). Consumables costs are adjusted for the failure rate (i.e. the percentage of consumables wasted, e.g. due to failed runs). Staff time is provided in terms of the minutes of hands-on staff time per sample, for both professionals and technicians. For the calculation of total costs, staff time is then monetised based on Eurostat data on country-specific labour costs for 2017 (by staff category), plus a 25% surcharge for overheads. For comparison purposes only, we have also provided staff costs monetised based on EU average labour costs. More detailed cost data is provided in Annex I.

a) Costs of using WGS

Sample preparation and sequencing	*Cost type*	*Cost per sample*
	Equipment costs	€ 1.74
	Consumables	€ 33.52
	Supplementary conventional tests	€ 3.68
	Staff time professionals	*6 minutes*
	Staff time technicians	*67 minutes*
	Staff costs, monetised based on labour cost data for the Netherlands (in brackets: based on labour cost data for the EU as a whole)	€ 36.85 (€ 31.87)
	**Total**	**€ 75.78**

Bioinformatics and other analyses	*Cost type*	*Cost per sample*
	Equipment costs	€ 0.76
	Other costs	€ 0
	Staff time professionals	*12 minutes*
	Staff time technicians	*24 minutes*
	Staff costs, based on labour cost data for the Netherlands (for EU)	€ 21.93 (€ 18.83)

	**Total**	**€ 22.69**
b) Costs of conventional methods		
Method A: Real Time PCR (plus sample preparation)	*Cost type*	*Cost per sample*
	Equipment costs	€ 0.98
	Consumables	€ 31.00
	Other costs	€ 0
	Staff time professionals	*0 minutes*
	Staff time technicians	*84 minutes*
	Staff costs, based on labour cost data for the Netherlands (for EU)	€ 39.53 (€ 34.30)
	**Total**	**€ 71.51**
Method B: Sanger Sequencing	*Cost type*	*Cost per sample*
	Equipment costs	€ 14.00
	Consumables	€ 23.75
	Other costs	€ 0
	Staff time professionals	*0 minutes*
	Staff time technicians	*60 minutes*
	Staff costs, based on labour cost data for the Netherlands (for EU)	€ 28.24 (€ 24.50)
	**Total**	**€ 65.98**
Method C: Virus isolation	*Cost type*	*Cost per sample*
	Equipment costs	€ 2.78
	Consumables	€ 10.00
	Other costs	€ 0
	Staff time professionals	*0 minutes*
	Staff time technicians	*30 minutes*
	Staff costs, based on labour cost data for the Netherlands (for EU)	€ 14.12 (€ 12.25)
	**Total**	**€ 26.90**
Method D: Hemagglutination inhibition	*Cost type*	*Cost per sample*
	Equipment costs	€ 6.04
	Consumables	€ 3.00
	Other costs	€ 0
	Staff time professionals	*5 minutes*
	Staff time technicians	*18 minutes*
	Staff costs, based on labour cost data for the Netherlands (for EU)	€ 12.90 (€ 11.11)
	**Total**	**€ 21.95**

Method E: Virus neutralisation	*Cost type*	*Cost per sample*
	Equipment costs	€ 6.21
	Consumables	€ 13.00
	Other costs	€ 0
	Staff time professionals	*5 minutes*
	Staff time technicians	*102 minutes*
	Staff costs, based on labour cost data for the Netherlands (for EU)	€ 52.43 (€ 45.41)
	**Total**	**€ 71.64**

Method F: NA Star	*Cost type*	*Cost per sample*
	Equipment costs	€ 2.07
	Consumables	€ 2.00
	Other costs	€ 0.00
	Staff time professionals	*0 minutes*
	Staff time technicians	*42 minutes*
	Staff costs, based on labour cost data for the Netherlands (for EU)	€ 19.77 (€ 17.15)
	**Total**	**€ 23.83**

IV. Costs of using WGS compared to the costs of conventional methods

The following comparison of costs per sample using WGS compared to the costs of conventional methods considers that the number of samples processed differed for the different conventional methods. The weighted cost of the conventional methods provided here is therefore a weighted figure which accounts for the use rate of the various methods across the different pathogens. See Annex I for more details.

Comparison of equipment costs

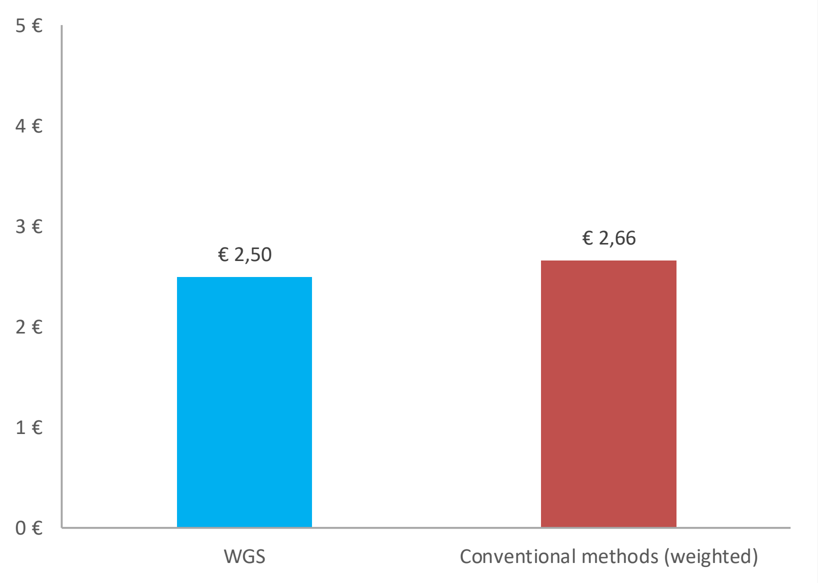

Equipment costs per sample at Erasmus MC are slightly lower for WGS than for the weighted conventional methods (€ 2.50 vs € 2.66), although the absolute per-sample cost difference between the two methods is quite low (€ 0.16). The lower cost of the GridION platform (about half the cost of second-generation sequencers like the MiSeq or IonTorrent) is one of the

	main factors keeping down WGS costs.
Comparison of costs of consumables	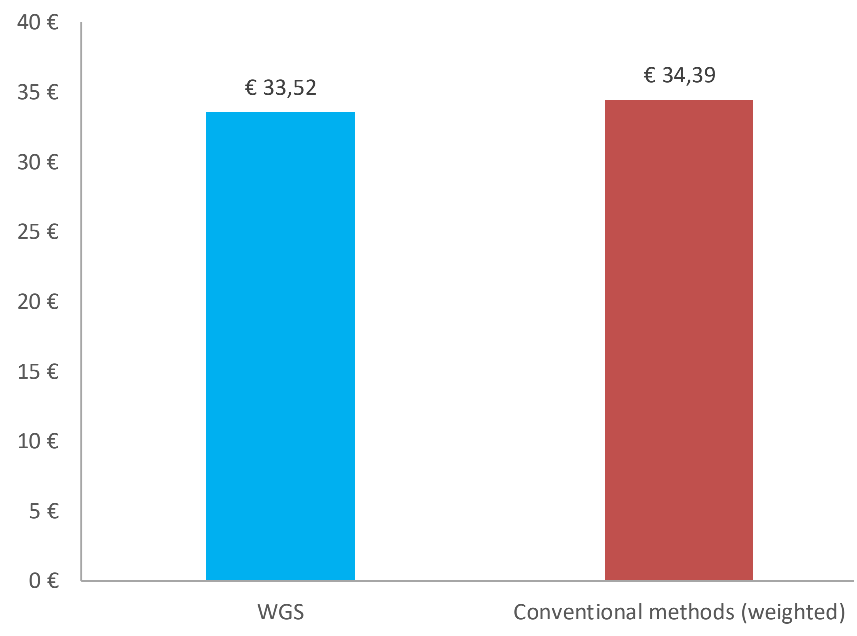<br><br>Consumables costs for WGS lie slightly below those for conventional methods (€ 33.52 vs € 34.39). The largest cost elements for WGS are the flowcells used for Nanopore sequencing (€ 11.22 per sample, for an average batch size of 30).
Comparison of staff time used (in minutes)	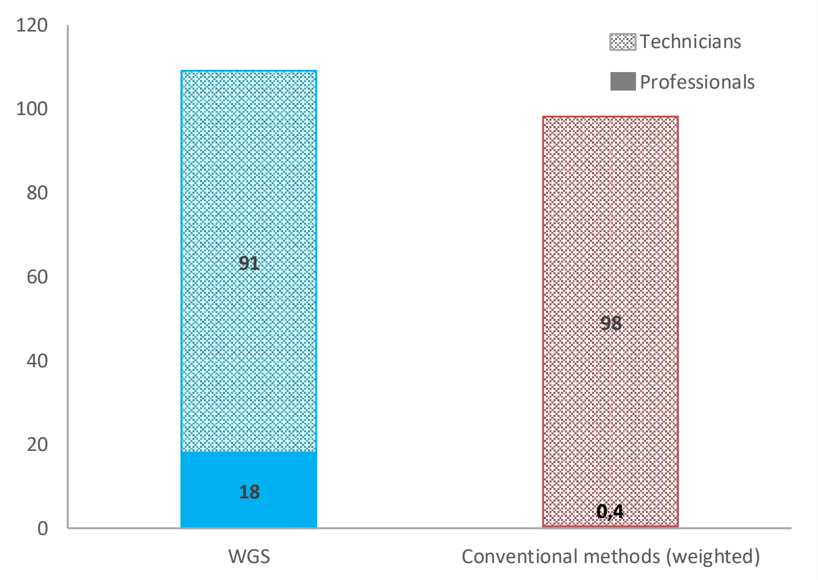<br><br>WGS requires slightly less technician staff time than conventional methods (91 minutes vs 98 minutes). It also requires an additional 18 minutes of professional time, mostly at the bioinformatics stage, whereas conventional methods on average require less than 1 minute of professional staff time – although this was noted to be due to troubleshooting required. Once monetised, staff costs are therefore still higher for WGS (€ 58.78) than for conventional methods (€ 46.31), and make up the most expensive cost item for WGS overall.
Comparison of other costs	As described above under 'Additional Information', a subset of samples continued to be subject to virus isolation, HI, virus neutralisation and NA star (conventional methods C-F) in parallel to WGS during the reference period. These have been accounted for in the WGS workflow as 'supplementary conventional tests', totalling € 3.68 per sample (note that this is the average

	cost across all 630 samples, which reflects the low intensity of the conventional testing that was carried out in parallel to WGS). No other costs were reported for the conventional methods workflow.

Comparison of overall costs	*Cost type*	*Cost per sample (WGS)*	*Cost per sample (conventional methods)*
	Equipment costs	€ 2.50	€ 2.66
	Consumables	€ 33.52	€ 34.39
	Other costs	€ 3.68 (for supplementary conventional tests in parallel to WGS)	€ 0
	Staff time professionals	*18 minutes*	*0.4 minutes*
	Staff time technicians	*91 minutes*	*98 minutes*
	Staff costs, based on labour cost data for the Netherlands (for EU)	*€ 58.78 (€ 50.70)*	€ 46.31 (€ 40.17)
	**Total**	**€ 98.48**	**€ 83.36**

Differential costs	**The cost difference between WGS and conventional methods is € 15.12 per sample**. A sample analysed with WGS costs approximately 18% more than analysis with conventional methods (when taking into account the use rate of the various methods). As indicated in the figures above, the largest differences are in staff costs.

V. Effects of using WGS results

a) Turnaround time. Turnaround time is defined as the usual number of days of work from receipt and opening of an incoming sample until the reporting of results. Turnaround time does not include weekends and holidays, except in case that work has been conducted on these days, e.g. for a sequencing run or other analyses.

Turnaround time	The turnaround time using the GridION is typically 2 days of work. This can be compressed to just 8-10 hours in an outbreak context, with some basic information about the sample available within the first 2-3 hours.<br>In contrast, the turnaround time for conventional methods (PCR and Sanger sequencing) is approximately 3 days of work. In an outbreak context, this can be brought down to about 20 hours with Sanger sequencing directly on clinical material, which is performed in parallel to cultivation of the virus (which still takes 3 days).<br>In an outbreak context, the average one day reduction in turnaround time due to WGS is reported to be very significant.

b) Positive effects of using WGS for pathogen identification and surveillance during the reference period

Sampling and sampling strategies	‣ No effects on sampling or sampling strategies were reported by Erasmus MC for 2018-2019, as they receive clinical samples submitted by hospitals. However, Erasmus MC considered that better sampling methods could be expected in the future as a result of WGS. Erasmus MC anticipates that the NGS-first surveillance will allow for the specific identification of samples that are worthy of further phenotypic characterisation, reducing this pipeline to a maximum of 12 samples annually (i.e. down from the 50 samples that were complementing the WGS workflow considered in this case study, see 'additional information', above).

Analytical results and processes	▸ Very significant positive effects were observed by Erasmus MC with respect to more detailed results produced due to NGS technology. This is due to the fact that all virus samples were now being sequenced, whereas prior to the introduction of the GridION only ~5% would have undergone further analysis using Sanger sequencing.<br>▸ Erasmus MC reported no effects on the accuracy or specificity of results, and in fact reported negative effects on the specificity of results (see 'Negative effects of WGS' below).<br>▸ Moderate effects were reported with respect to a reduction in time needed for analysis. While the hands-on staff time needed increased for WGS compared to conventional methods, overall a reduction in turnaround time was reported for WGS (see above). This is due to the fact that more waiting periods (e.g. for viral amplification) are required for conventional methods compared to WGS. No effects were observed with respect to simplified workflows or a reduction in consumables.
Outbreak identification and response	▸ Erasmus MC reported very significant positive effects for the earlier detection of an initial outbreak and for improved detection that outbreaks are related. However, it specified that in an international context, the benefits from improved detection that outbreaks are related depended on whether partner institutions had also adopted WGS. It indicated that the benefits of WGS for detection of international outbreaks were limited if the partners still relied on conventional methods, as the results from these methods were often not comparable with results from WGS.<br>▸ Erasmus MC indicated that it had insufficient information with respect to possible effects on improved information through WGS for imposing additional control measures or reductions in the duration of an outbreak, in the number of secondary outbreaks, or in overall costs for outbreak identification and response. However, such effects were considered very likely to materialise in the long run (especially for other pathogens). For example, it indicated that the faster turnaround time with Nanopore sequencing could allow patients to be isolated earlier or receive more personalised medical treatment (however, this was not considered to be relevant with respect to the case study pathogen).
Research and methods applied	▸ No concrete effects on research or methods applied were reported by Erasmus MC.
Effects on wider society	▸ No concrete effects on wider society were observed by Erasmus MC during the case study period, although it was considered that such effects would likely emerge over time.
c) Negative effects of using WGS	
Negative effects of using WGS	▸ Erasmus MC reported negative effects on the sensitivity of results with WGS due to the fact that it now skips the viral cultivation step and uses a PCR approach directly on clinical samples. This is reported to save time, but results in slightly less sensitivity (535 test results on 630 samples). Erasmus MC clarified that this is a 'problem' of internal workflow, however, not of the technology, and that the problem is not limited to Nanopore sequencing but concerns WGS in general.<br>▸ Erasmus MC reported limitations of Nanopore sequencing related to a failure of basecalling for homopolymeric regions in the sequences (i.e. errors in reading multiples of the same nucleotide base appearing consecutively in the DNA sequences). Erasmus MC indicated that this is a known problem specific to Nanopore sequencing and that the technology is expected to improve in the near future.

VI. Outlook	
Balance of costs and benefits achieved	▸ Erasmus MC indicated that Nanopore sequencing is a 'game changer', yet not as much as they would like due to the high prices of the required flowcells. While the costs are lower compared to e.g. Illumina sequencing, the costs are still significant. However, it was also noted that in an outbreak context 'time is more important than money', and the reduction in turnaround time was therefore considered to be very valuable.
Potential for cost reductions	▸ Erasmus MC considered that current prices (e.g. for flowcells) were relatively high, and that substantial cost reductions could be achieved through negotiation with suppliers, or increased competitive pressure.<br>▸ Erasmus MC indicated that the 2018-2019 season included professional staff time spent troubleshooting issues with the WGS workflow, and that this would likely be substantially less in future seasons.<br>▸ Erasmus MC reported that costs could be further reduced by automation of the RNA isolation process during library preparation, and by loading higher sample volumes (e.g. up to 40 samples) on a single flowcell.
Future opportunities and challenges	▸ Erasmus MC considered that Nanopore sequencing technology was constantly improving, with the above mentioned failure of basecalling for homopolymeric regions likely to be fixed in the very near future.<br>▸ The high price of the flowcells, which are only provided by one company (Oxford Nanopore), was noted as a challenge by Erasmus MC. The company also places contractual restrictions on the use of the flowcells purchased through the institutional contract between Erasmus MC and Oxford Nanopore, e.g. regarding their use outside the premises of Erasmus MC, and thereby limiting usefulness for field research and real-time analysis of outbreaks by Erasmus MC staff visiting other countries, such as China (however, the contract is in the process of being re-negotiated to remove these geographical restrictions at least partly).<br>▸ Erasmus MC reported that better communication was needed with hospitals to ensure that the hospitals send samples with higher viral loads in the future in order to counteract the lower sensitivity that can result from the use of metagenomic analysis without viral amplification.

VII. Key sources/references	
Cost questionnaire	Cost questionnaire completed by Erasmus MC
Preparatory phone interview	a) Background information and description of activities
Case study visit and follow up	b) Additional data and clarifications provided by the institution.
Scientific literature	As Nanopore sequencing was introduced for routine influenza surveillance at Erasmus MC for the first time during the case study period, no scientific literature related to the case study has been published yet by Erasmus MC.
Other	c) Erasmus MC Department of Viroscience website, https://www6.erasmusmc.nl/viroscience/

4.4. Istituto Zooprofilattico Sperimentale della Lombardia e dell'Emilia-Romagna (IZSLER)

**Salmonella and Listeria surveillance – IZSLER, Italy**	
**I. Institution**	
**Name of institution**	Istituto Zooprofilattico Sperimentale della Lombardia e dell'Emilia-Romagna (IZSLER)
**Type of institution**	Public veterinary institution
**Description**[19]	The Lombardy and Emilia-Romagna Experimental Zooprophylaxis Institute (IZSLER) is a public body entrusted with independent management, administrative and technical powers. It operates as a technical scientific institution of the state, the regions and the autonomous provinces.<br>IZSLER's territory of jurisdiction comprises the regions of Lombardy and Emilia-Romagna in northern Italy and it is part of a network of regional institutes that covers all of Italy.<br>The Institute's main tasks are the following:<br>▸ Animal diseases and zoonoses diagnostic service;<br>▸ Laboratory control on foodstuffs for human and animal consumption;<br>▸ Epidemiological monitoring in the ambit of animal health and in that of hygiene of zootechnic and foodstuff production;<br>▸ Analytic and advisory support to the carrying out of epidemic prevention, sanitation and eradication plans;<br>▸ Applied research in the field of breeding hygiene and improvement of zootechnic production and animal wellbeing;<br>▸ Applied and basic experimental research in the veterinary and food area.<br>IZSLER's High Specialisation Centres carry out highly specialised activities in the field of animal health, food hygiene and zootechnic hygiene. In particular, IZSLER was appointed as the National Reference Centre for numerous diseases by the Ministry of Health, as the OIE Reference Laboratory for Foot-and-Mouth Disease, Swine Influenza, Myxomatosis, and Haemorrhagic Diseases of Lagomorphs, and as the FAO collaboration centre for Foot-and-Mouth Disease.
**Location**	While IZSLER's main office is located in Brescia, Italy, units are distributed on a provincial basis to cover the Lombardy Territorial Area and the Emilia-Romagna Territorial Area.
**II. Surveillance activities covered by case study**	
**Activity**	Routine laboratory surveillance
**Reference period**	01/2017 – 12/2017
**Pathogen(s) covered**	Salmonella, Listeria
**Summary of routine surveillance activities using WGS**	Since 2012, IZSLER routinely processes isolates of *Salmonella enterica* from human, animal and food sources as part of the One Health surveillance of foodborne infections based on PFGE, MLVA and serotyping. Isolates belonging to significant outbreaks have been sequenced and compared with SNPs and Gene-by-Gene approaches to highlight phylogenetic relationships and attribute source of infections. The same workflow is applied to isolates

[19] Source: http://www.izsler.it/izs_home_page/who_we_are_/00000047_English.html

	of *Listeria monocytogenes*. WGS is currently used as a confirmation method, and has also been used to retrospectively study past outbreaks.[c)-f)] The reference period of 2017 was a transition year, which extended to include 2018; the institute will switch to the full routine use of WGS in 2019, thereby stopping the use of conventional methods in parallel. The main reason for this is the information potential of whole genome sequencing and the potential for improving surveillance/public health. According to IZSLER, this was also requested by the industry, as major food producers, including export industries, are located in the region, e.g. in Parma.		
**Type of sample**	Isolates		
**Region covered by sampling**	Emilia-Romagna, Italy		
**Number of samples analysed in reference period**	*Pathogen*	*Samples analysed by conventional methods*	*Samples sequenced using WGS*
	Salmonella	1500	110 (7.3% of samples)
	Listeria	65	65 (100% of samples)
**Conventional methods used**	▸ Salmonella: Serotyping (100% of samples), PFGE (100%), PCR Verification for Typhimurium (50%), MLVA (60%)<br>▸ Listeria: PFGE (100%)		
**Sample preparation WGS**	▸ Manual		
**Sequencer used for WGS**	▸ MiSeq (Illumina)		
**Batch size for WGS analysis**	▸ The typical batch size for WGS analysis during the reference period was 24.		
**Reference dataset used for WGS**	▸ IZSLER uses its own reference dataset based on the analyses conducted, and regularly checks international databases for relevant new entries, which are then included into the database if necessary. The institution indicated that public databases have the advantage that data is available and can always be re-analysed, but noted that issues remain regarding data and metadata quality in such public databases.		
**Additional information**	▸ In the reference year, the institute had not used WGS to identify outbreaks but only to confirm or further analyse outbreaks that had already been identified through the use of conventional methods. Therefore, all sequenced isolates had already been typed using conventional methods.<br>▸ As indicated above, IZSLER has responsibilities with regard to both animal health and food safety. For the two pathogens covered by this case study, the institute routinely analyses isolates originating from animal infections, food samples, and human cases of infection, as part of a One Health approach to surveillance.		

III. Detailed overview of costs of WGS and conventional methods

In the following, all costs are provided on a per-sample basis. Equipment costs are annualised and incorporate the annual maintenance costs as reported by the institution. They are adjusted for the percentage use of the equipment for the listed pathogens samples during the reference period (i.e. if a sequencer was also used for other purposes, this is taken into account). Consumables costs are adjusted for the failure rate (i.e. the percentage of consumables wasted, e.g. due to failed runs). Staff time is provided in terms of the minutes of hands-on staff time per sample, for both professionals and technicians. For the calculation of total costs, staff time is then monetised based on Eurostat data on country-specific labour costs for 2017 (by staff category), plus a 25% surcharge for overheads. For comparison purposes only, we have also provided staff costs monetised based on EU average labour costs.

More detailed cost data is provided in Annex I.

a) Costs of using WGS

Sample preparation and sequencing	*Cost type*	*Cost per sample*
	Equipment costs	€ 123.07
	Consumables	€ 165.37
	Other costs	€ 0
	Staff time professionals	*0 minutes*
	Staff time technicians	*35 minutes*
	Staff costs, monetised based on labour cost data for Italy (in brackets: based on labour cost data for the EU as a whole)	€ 13.93 (14.29)
	**Total**	**€ 302.38**

Bioinformatics and other analyses	*Cost type*	*Cost per sample*
	Equipment costs	€ 40.41
	Other costs	€ 0
	Staff time professionals	*70 minutes*
	Staff time technicians	*0 minutes*
	Staff costs, based on labour cost data for Italy (for EU)	€ 52.35 (52.65)
	**Total**	**€ 92.77**

b) Costs of conventional methods

Serotyping (used for 100% of Salmonella samples)	*Cost type*	*Cost per sample*
	Equipment costs	€ 0
	Consumables	€ 7.76
	Other costs	€ 0
	Staff time professionals	*3 minutes*
	Staff time technicians	*38 minutes*
	Staff costs, based on labour cost data for Italy (for EU)	€ 17.36 (17.77)
	**Total**	**€ 25.12**

PFGE (100% of Salmonella and Listeria samples)	*Cost type*	*Cost per sample*
	Equipment costs	€ 22.84
	Consumables	€ 14.42
	Other costs	€ 0.00
	Staff time professionals	*2.5 minutes*
	Staff time technicians	*38 minutes*
	Staff costs, based on labour cost data for Italy (for EU)	€ 16.99 (17.40)
	**Total**	**€ 54.25**
PCR Verification for Typhimurium (50% of Salmonella samples)	*Cost type*	*Cost per sample*
	Equipment costs	€ 10.18
	Consumables	€ 2.78
	Other costs	€ 0
	Staff time professionals	*1 minute*
	Staff time technicians	*11 minutes*
	Staff costs, based on labour cost data for Italy (for EU)	€ 4.73 (4.84)
	**Total**	**€ 17.68**
MLVA (60% of Salmonella samples)[20]	*Cost type*	*Cost per sample*
	Equipment costs	€ 0
	Consumables	€ 0
	Other costs	€ 43.13
	Staff time professionals	*0 minute*
	Staff time technicians	*0 minute*
	Staff costs, based on labour cost data for Italy (for EU)	€ 0 (0)
	**Total**	**€ 43.13**

[20] Note that ISZLER has MLVA conducted externally by another lab in the network and therefore incurs no staff, consumables, or equipment costs of its own. The cost shown here is the estimated cost price.

IV. Costs of using WGS compared to the costs of conventional methods	
The following comparison of costs per sample using WGS compared to conventional methods takes into account the fact that the number of samples processed differed between conventional methods, e.g. serotyping is used for 100% of Salmonella samples, but MLVA is only used for 60% of Salmonella samples. The average cost of the conventional methods provided here is therefore a weighted figure which accounts for the use rate of the various methods. See Annex I for more details.	
Comparison of equipment costs	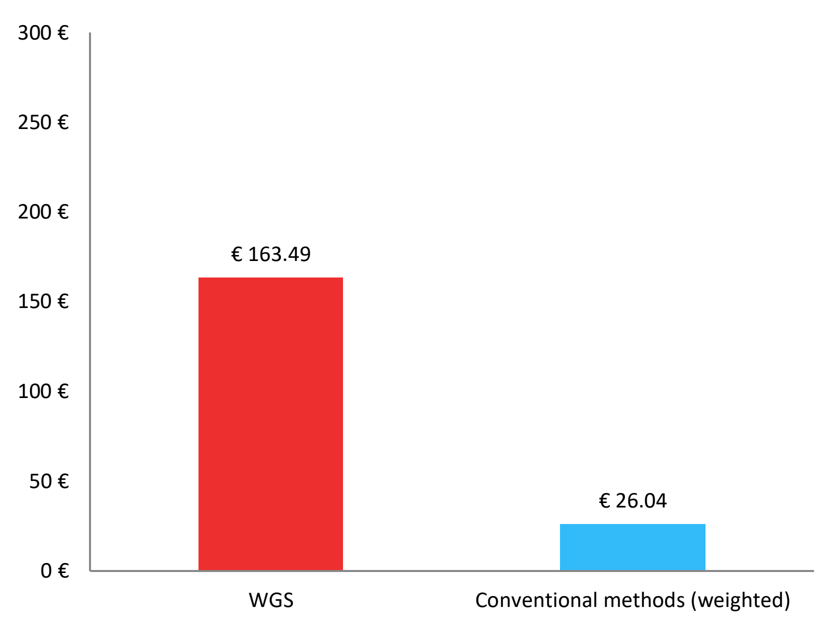 Equipment costs are significantly higher for WGS (€ 163.49 vs. € 26.04 per sample), mostly due to purchase and maintenance costs of the sequencer itself. IZSLER indicated during the case study visit that larger sequencers were generally better from a cost perspective, but require a large batch size to be cost-effective. However, in a surveillance context it is not always possible to postpone analysis until a certain number of samples have accumulated.
Comparison of costs of consumables	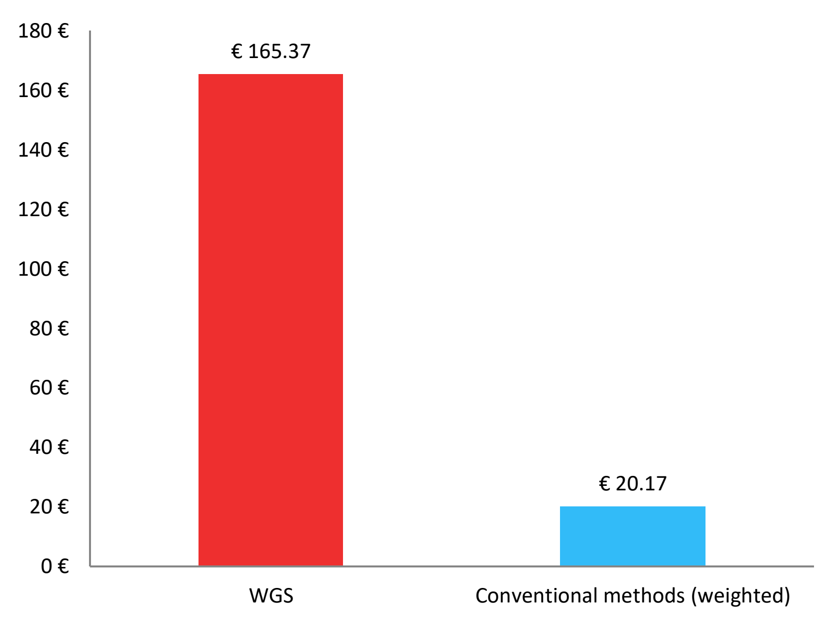 Costs of consumables for WGS are also higher than the weighted average of

conventional methods (€ 165.37 vs. € 20.17 per sample), due to the cost of consumables used for library preparation (€ 46.85 per sample using WGS) and even more importantly the cost of consumables used for sequencing (€ 114.20 per sample using WGS).

Comparison of staff time used

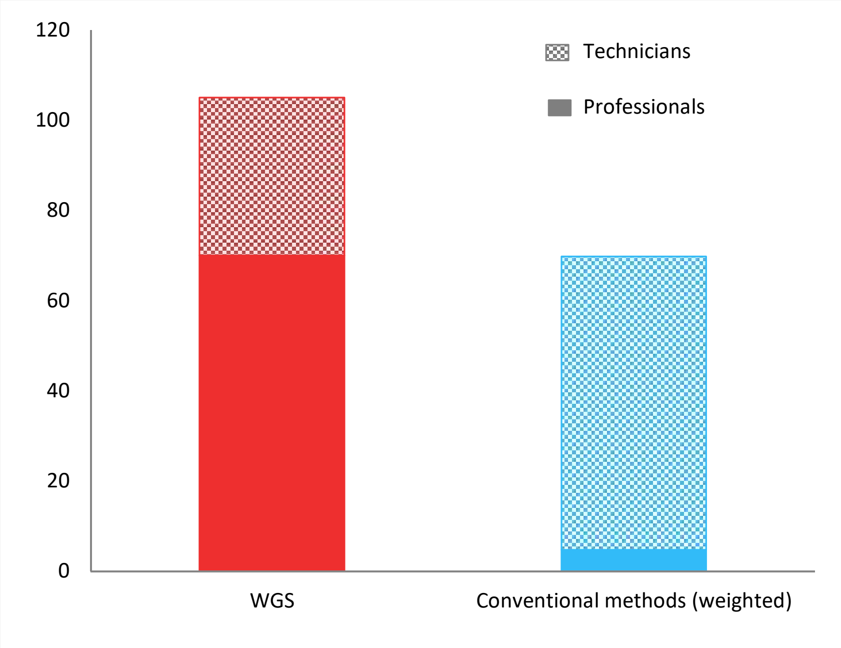

The amount of staff time needed for WGS is higher than for conventional methods, and the proportion of professionals' time to technicians' time is much larger for WGS. This is entirely due to the bioinformatics analysis required for WGS, as this stage is performed exclusively by professional staff, while sample preparation and sequencing are conducted exclusively by technicians. However, IZSLER indicated during the case study visit that they anticipated the bioinformatics stage to be automated for routine surveillance in the future.

Taking the different staff categories into account, monetised staff costs per sample for WGS are approximately two times the amount required for conventional methods (see table below).

Comparison of overall costs

Cost type	*Cost per sample (WGS)*	*Cost per sample (conventional methods)*
Equipment costs	€ 163.49	€ 26.04
Consumables	€ 165.37	€ 20.17
Other costs	€ 0	€ 16.27
Staff time professionals	*70 minutes*	*5 minutes*
Staff time technicians	*35 minutes*	*65 minutes*
Staff costs (monetisation based on labour cost data for Italy)	€ 66.28	€ 29.39
Staff costs (monetisation based on labour cost data for the EU)	*€ 66.94*	*€ 30.09*

	**Total**	**€ 395.14**	**€ 91.87**

Summary of differential costs	**The cost difference between WGS and conventional methods is € 303.27 per sample**. A sample analysed with WGS costs more than four times the amount of conventional methods (**€** 395.14 vs **€** 91.87). As indicated in the figures above, this difference is mainly due to consumables costs and equipment costs.

V. Effects of using WGS results

a) Turnaround time. Turnaround time is defined as the usual number of days of work from receipt and opening of an incoming sample until the reporting of results. Turnaround time does not include weekends and holidays, except in case that work has been conducted on these days, e.g. for a sequencing run or other analyses.

Turnaround time	▸ The turnaround time for the analysis of a sample using WGS for pathogen identification is 7 days of work, compared to 10 days of work for using the specified conventional method(s) for pathogen identification.

b) Positive effects of using WGS for pathogen identification and surveillance during the reference period

Sampling and sampling strategies	▸ Little or no positive effects of using WGS on sampling and sampling strategies are expected from IZSLER's perspective as these are not the institution's responsibility and are independent from the institution's laboratory function. In addition, the number of samples is largely independent from the method used for analysis.
Analytical results and processes	▸ IZSLER considered that the use of WGS for pathogen identification and surveillance has led to very significant positive effects on analytical results and processes. It reported significant improvement regarding the accuracy, sensitivity and specificity of results produced.<br>▸ IZSLER also indicated that WGS had led to simplified laboratory work flows, *inter alia* through the reduction of the number of hands-on steps. It also considered that WGS had led to a reduction in the amount of consumables needed for analysis and in staff time required.
Outbreak identification and response	▸ IZSLER considered that the use of WGS for pathogen identification and surveillance has led to very significant positive effects for outbreak identification and response, and sees a reduction in the related overall costs.<br>▸ IZSLER reported significant improvements regarding earlier detection of initial outbreaks, detection that isolates are related, and information on outbreak epidemiology (e.g. linking cases to the source). In the institution's experience, the high resolution power of WGS is making a striking difference in pathogen typing and source attribution; this was the finding of several scientific papers published by IZSLER retrospectively examining past salmonella and listeria outbreaks using WGS.[c)-f)] In particular, a 2018 paper published by IZSLER using WGS to examine an outbreak of salmonella in 2013 concluded that PFGE and MLVA did not have the necessary resolution or accuracy, respectively, to reliably link isolates to the outbreak source, and could in fact produce misleading results.[c)-f)]<br>▸ Substantial advantages of WGS were therefore found to derive from the superior accuracy in the attribution of contamination responsibilities along the food chain. For example, during the above mentioned outbreak in 2013, the PFGE based surveillance system identified an outbreak of monophasic *Salmonella Typhimurium* with the potential involvement of a salami producer, a specific abattoir and a farmer. WGS and phylogenetic analyses were able to confirm the salami producer involvement in the case

	but cleared both the farmer and the abattoir of any responsibility.[d)]<br>▸ IZSLER considered that WGS has also led to significant improvements regarding the information for imposing additional control/biosecurity measures. For instance, the nature (monoclonal vs polyclonal) and distribution of contamination inside food-processing facilities can be finely reconstructed by WGS. As a consequence, de-contamination of facilities can be managed and verified with high confidence.<br>▸ As regards the surveillance of human infections, IZSLER also considered that WGS helps identify true outbreaks, thus preventing false alerts to public health officials, and reducing the number of infections. The above quoted scientific paper concluded that, had WGS been in routine use at the time of the 2013 outbreak, the source of the outbreak could have potentially been identified up to two months earlier, possibly preventing dozens of infections if the correct mitigation measures had been taken in time. IZSLER considered that this is improving consumers' confidence in the competent authorities and in food business operators.
Research and methods applied	▸ Regarding the positive effects on research and methods applied, the institution reported very significant improvement in the understanding of disease transmission and a positive impact on epidemiological investigations.
Effects on wider society	▸ IZSLER indicated that the use of WGS has led to a significant reduction in the negative effects of food chain contamination on industry and trade relationships, and provided the example of a controversy between two operators of the Parma Ham industry following the finding of *Listeria monocytogenes* with the same PFGE type in their plants. The plants operated sequentially along the same processing chain; one was the ham producer and the second was the deboner. Considering the apparently identical contamination (based on PFGE), the operators blamed each other as the source of the contamination. WGS was able to clearly demonstrate that the isolates from the deboner and producer were unrelated despite identical having an identical PFGE type. As a result, not only were both required to improve their own hygiene procedures, but also no further commercial or legal controversy was justified.<br>▸ The positive impact on the food industry is also evidenced by the interest of operators in WGS and the fact that major operators have started doing their own in-house testing with WGS.
c) Negative effects of using WGS	
Negative effects of using WGS	So far, the use of WGS for pathogen identification and surveillance has not had negative effects for IZSLER, other than the currently higher costs compared to conventional methods.<br>However, IZSLER indicates that the high resolution power of WGS might lead to the identification of a high number of smaller outbreaks which may strain existing (staff and analytical) capacities.
**VI. Outlook**	
Balance of costs and benefits achieved	▸ IZSLER noted that in comparison with conventional methods, using WGS is currently more expensive but should eventually reach comparable cost levels, while providing more information.
Potential for cost reductions of using WGS for pathogen identification and surveillance in the future	▸ There is a high potential for simplification of the type of samples needed for WGS with the use of metagenomics. However, IZSLER indicated that this is not expected to materialise for another 5 to 10 years.<br>▸ It is also expected that significant cost reduction for WGS could be achieved by scaling-up the analytical process through automation of the

(through e.g. techno-logical advances)	DNA extraction and library preparation steps. IZSLER considered that the process could eventually be almost entirely automated.<br>▸ Savings in the number of required staff are expected: the number of required staff for Salmonella analysis is expected to be at least halved, while maintaining the same staff categories.<br>▸ Technological developments might have an impact on equipment costs, although the institution noted that it is difficult to foresee how the situation will develop regarding sequencers and related equipment in the coming years.
Future opportunities and challenges	▸ The cross-pathogen potential of WGS technology is a very important advantage from IZSLER's perspective. While many conventional typing methods are pathogen-specific, using WGS can reduce the variety of methods to a single technique or to a single process. The institution noted that it is very confident that using WGS will simplify the analytical process and will improve the overall management of the laboratory.<br>▸ With WGS, IZSLER indicated that it will be able to satisfy a broader range of requests from public health labs, e.g. on Campylobacter, as WGS would allow them to easily switch to another pathogen in cases where there is ad hoc need to support an outbreak investigation.<br>▸ As indicated above, the use of WGS may lead to the identification of a high numbers of matches, i.e. potential outbreaks, which raises the question of whether they would have capacity to investigate these potential outbreaks and of the definition of an outbreak. There could also be a need for further standardisation on the approach for outbreak investigation.<br>▸ IZSLER noted that with the current uptake of and growing interest in WGS there is a potential for fragmentation of the system, and emphasised the importance of standards for sequencing and sharing of results. According to IZSLER quality issues with respect to public databases also indicate a need for further standards and quality assurance in this respect.

**VII. Key sources/references**	
Cost questionnaire	Cost questionnaire completed by IZLER
Preparatory phone interview	a) Background information and description of activities
Case study visit and follow up	b) Additional data and clarifications provided
Scientific literature	c) Comandatore, F., et al (2017). Genomic Characterization Helps Dissecting an Outbreak of Listeriosis in Northern Italy. *PLoS Currents*, 9, 1–21. http://doi.org/10.1371/currents.outbreaks.633fd8994e9f06f31b3494567c7e504c<br>d) Morganti, M., et al. (2018). Rise and fall of outbreak-specific clone inside endemic pulsotype of salmonella 4,[5],12:i:-; insights from high resolution molecular surveillance in Emilia-Romagna, Italy, 2012 to 2015. *Eurosurveillance*, 23(13), 1–11. http://doi.org/10.2807/1560-7917.ES.2018.23.13.17-00375<br>e) Morganti, M., et al. (2015). Processing-dependent and clonal contamination patterns of Listeria monocytogenes in the cured ham food chain revealed by genetic analysis. *Applied and Environmental Microbiology*, 82(3), 822–831. http://doi.org/10.1128/AEM.03103-15<br>f) Scaltriti, E., et al. (2015). Differential single nucleotide polymorphism-based analysis of an outbreak caused by Salmonella enterica serovar Manhattan reveals epidemiological details missed by standard pulsed-field gel electrophoresis. *Journal of Clinical Microbiology*, 53(4), 1227–1238. http://doi.org/10.1128/JCM.02930-14

4.5. Administración Nacional de Laboratorios e Institutos de Salud (ANLIS)

**Salmonella and E. coli surveillance – ANLIS, Argentina**	
**I. Institution**	
**Name of institution**	Instituto Nacional de Enfermedades Infecciosas - Administración Nacional de Laboratorios e Institutos de Salud (INEI-ANLIS)
**Type of institution**	Public institution under the Ministry of Health
**Description**	The National Administration of Laboratories and Health Institutes is an organisation that implements the policies of the Argentinian Ministry of Health with respect to the prevention, referential diagnostics, research, and treatment of infectious, genetic, nutrition-based and non-transmissible diseases. It is also responsible for the production and quality control of immunobiological products, for the execution of health programs related to its areas of responsibility, for the coordination of laboratory networks in the country, and in the conduct of epidemiological studies.<br>The National Institute for Infectious Diseases at ANLIS conducts and collaborates in research and methodological development concerning infectious diseases including zoonoses, foodborne infections, water infections and new microbial etiologies. It acts as the national reference laboratory for the diagnosis of viral, bacterial, fungal, and parasitic diseases.
**Location**	Buenos Aires, Argentina
**II. Surveillance activities covered by case study**	
**Activity**	Routine laboratory surveillance
**Reference period**	06/2017 – 05/2018
**Pathogen(s) covered**	Salmonella, E. coli
**Summary of routine surveillance activities using WGS**	WGS has been used at INEI-ANLIS for the routine surveillance of foodborne pathogens since 2015, having been introduced as part of a WHO Pilot Project in cooperation with the GenomeTrakr programme at the US Food and Drug Administration (US-FDA).[e-h] Although WGS has been implemented on a routine basis for Salmonella, E. coli and Shigella, conventional methods are still being used in parallel for these pathogens due to concerns regarding the cost and availability of the relevant reagents. There are currently no plans to replace these conventional methods in the short-term.<br>The surveillance of foodborne pathogens in Argentina is conducted through the National Diarrheal Network, in which food and clinical laboratories from the whole country participate. Depending on the pathogens, they send a number of the isolates identified to INEI-ANLIS. For Salmonella subspecies, local and provincial laboratories have the capacity to serotype the two most common serovars of Salmonella in Argentina (Salmonella enterica ser. Typhimurium and Salmonella enterica ser. Enteritidis). From these two serovars, local laboratories are required to send each month 20% of their isolates to INEI-ANLIS for further analysis. However, local laboratories must send all other serovars they isolate. To study circulating clones, INEI-ANLIS serotypes all isolates received and uses PFGE for all Salmonella enterica ser. Enteritidis and Typhimurium isolates received and for a selection of the other serovars, as well as all suspected outbreak isolates. For WGS surveillance a selection of all the isolates received at INEI-ANLIS is sequenced, including all suspected outbreak isolates.
**Type of sample**	Isolates (for E. Coli only: also samples)

**Region covered by sampling**	Argentina		
**Number of samples analysed in reference period**	*Pathogen*	*Samples analysed by conventional methods*	*Samples sequenced using WGS*
	Salmonella	The cost calculation is based on experiences with the listed conventional methods, assuming the same number of samples as with WGS	128
	E. Coli		192
**Conventional methods used**	- Salmonella: Biochemical testing (100% of samples), Serotyping (100%), MaldiTOF (5%), PFGE (70%)<br>- E. coli: Biochemical testing (100% of samples), PCR typing (100%), MaldiTOF (5%), PFGE (100%)		
**Sample preparation WGS**	- Manual preparation of isolates		
**Sequencer used for WGS**	- Illumina MiSeq		
**Batch size for WGS analysis**	- The typical batch size for WGS analysis during the reference period was 16 samples per run.		
**Reference dataset used for WGS**	INEI-ANLIS uses genomic data from publically available databases which is then complemented with genomic data from its own sequencing activities.		

III. Detailed overview of costs of WGS and conventional methods

In the following, all costs are provided on a per-sample basis. Equipment costs are annualised and incorporate the annual maintenance costs as reported by the institution. They are adjusted for the percentage use of the equipment for the listed pathogens samples during the reference period (i.e. if a sequencer was also used for other purposes, this is taken into account). Consumables costs are adjusted for the failure rate (i.e. the percentage of consumables wasted, e.g. due to failed runs). Staff time is provided in terms of the minutes of hands-on staff time per sample, for both professionals and technicians. For the calculation of total costs, staff time is then monetised based on estimated labour costs provided by INEI-ANLIS, plus a 25% surcharge for overheads.

More detailed cost data is provided in Annex I.

a) Costs of using WGS

Sample preparation and sequencing	*Cost type*	*Cost per sample*
	Equipment costs	€ 35.45
	Consumables	€ 104.62
	Other costs	€ 0.00
	Staff time professionals	*31 minutes*
	Staff time technicians	*0 minutes*
	Staff costs, monetised based on labour cost data for Argentina	€ 2.33
	**Total**	**€ 142.40**

Bioinformatics and other analyses	*Cost type*	*Cost per sample*
	Equipment costs	€ 7.57
	Other costs	€ 0.00
	Staff time professionals	*60 minutes*
	Staff time technicians	*0 minutes*

	Staff costs, based on labour cost data for Argentina	€ 4.52
	**Total**	**€ 12.09**
b) Costs of conventional methods[21]		
Biochemical testing and serotyping (used for 100% of Salmonella samples)	*Cost type*	*Cost per sample*
	**Total**	**€ 35.41**
Biochemical testing and PCR (100% of E. Coli samples)	*Cost type*	*Cost per sample*
	**Total**	**€ 39.83**
MaldiTOF (5% of Salmonella and 5% of E. coli samples)	*Cost type*	*Cost per sample*
	**Total**	**€ 61.96**
PFGE (70% of Salmonella samples and 100% of E. coli samples)	*Cost type*	*Cost per sample*
	**Total**	**€ 6.64**

IV. Costs of using WGS compared to the costs of conventional methods

The following comparison of costs per sample using WGS compared to conventional methods takes into account the fact that the number of samples processed differed between conventional methods, e.g. biochemical testing is used for 100% of Salmonella samples, but MaldiTOF is only used for 5% of Salmonella samples. The average cost of the conventional methods provided here is therefore a weighted figure which accounts for the use rate of the various methods. See Annex I for more details.

Comparison of overall costs	*Cost type*	*Cost per sample (WGS)*	*Cost per sample (conventional methods)*
	Equipment costs	€ 43.02	-
	Consumables	€ 104.62	-
	Other costs	€ 0.00	-
	Staff time professionals	*91 minutes*	-
	Staff time technicians	*0 minutes*	-
	Staff costs (monetisation based on labour cost data for Argentina)	€ 6.85	-
	**Total**	**€ 154.49**	**€ 46.61**

[21] Note that costs for conventional methods were provided as lump sum figures representing the costs that were charged to external clients for the relevant tests, including equipment, consumables and staff time.

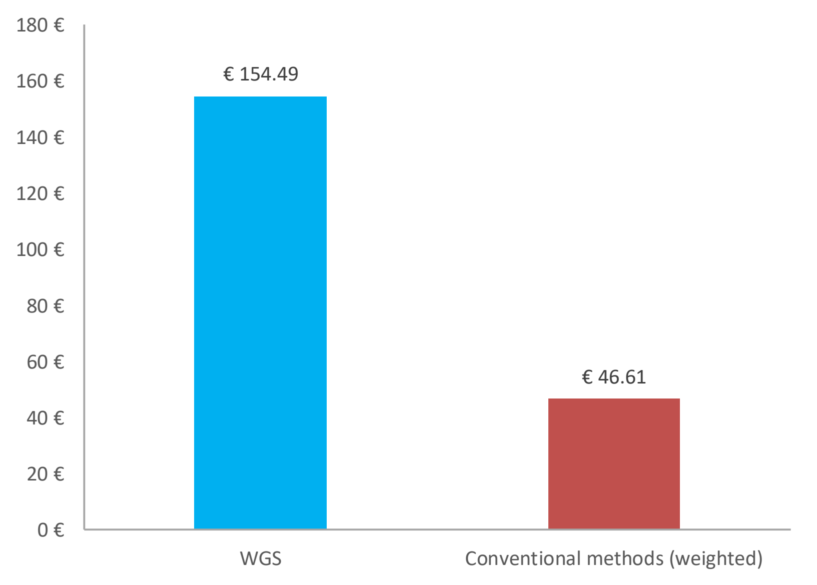

Summary of differential costs	**The cost difference between WGS and conventional methods is € 107.88 per sample**. A sample analysed with WGS costs approximately 3.3 times the amount of conventional methods (€ 154.49 vs € 46.61).

V. Effects of using WGS results

a) Turnaround time. Turnaround time is defined as the usual number of days of work from receipt and opening of an incoming sample until the reporting of results. Turnaround time does not include weekends and holidays, except in case that work has been conducted on these days, e.g. for a sequencing run or other analyses.

Turnaround time	The turnaround time for the analysis of a sample using WGS can last 5-10 days. In the case of an outbreak where the isolates are prioritised for analysis, this can be reduced to 5-6 days.<br>The turnaround time using conventional methods lasts:<br>▸ 4-7 days for pathogen identification at the species level;<br>▸ 5-15 days for characterisation (including the serotype and toxin profile for E. coli); and<br>▸ 5 days must be added for identification of clonal relationship of isolates using PFGE.<br>In a salmonella outbreak, for example, the complete turnaround time for all three steps using conventional methods can last between 7 and 15 days.

b) Positive effects of using WGS for pathogen identification and surveillance during the reference period

Sampling and sampling strategies	▸ Little or no positive effects of using WGS on sampling and sampling strategies were identified by INEI-ANLIS, although it considered that there could be a minor effect on the simplification of sample storage or transport.
Analytical results and processes	▸ INEI-ANLIS considered that the use of WGS had significant effects on improved accuracy, sensitivity, specificity and level of detail of the results produced. Results of the WHO Pilot Project to introduce WGS in Argentina also showed that WGS could obtain additional information on virulence factors.[f)]<br>▸ INEI-ANLIS also indicated that WGS had led to simplified laboratory work flows and could lead to a substantial reduction in required staff numbers (if it were fully implemented and used to replace conventional methods such

	as serotyping or PFGE). However, it did not see any effects on the reduction of staff time needed for the analysis (see also above the comparison of staff time used for WGS and conventional methods), due to the increased staff time needed for the bioinformatics analysis.
Outbreak identification and response	▸ INEI-ANLIS considered that the use of WGS for pathogen identification and surveillance had significant effects with respect to improved detection that outbreaks are related and improved information on outbreak epidemiology. It cited scientific publications by its staff showing the use of WGS in retrospectively distinguishing between multiple outbreaks of Shigella sonnei in Argentina.[c-d] The study showed that even with a lack of supporting routine data WGS was an indispensable method for the tracking and surveillance of bacterial pathogens during outbreaks and was becoming a vital tool for the monitoring of antimicrobial resistant strains of *S. sonnei*.[d]<br>▸ The WHO Pilot Project concluded, however, that maximising the benefit of genomic outbreak data requires long-term contextual (i.e. routine surveillance) data from local and international sources.[g]<br>▸ INEI-ANLIS did not report any effects with respect to improved information for imposing additional control or biosecurity measures, nor did it indicate any effects concerning a reduction in the duration of an outbreak or a reduction in the disease burden for humans. INEI-ANLIS reported that this was due to the delay in receiving samples (see description of surveillance system above), so that typically the outbreak is already detected at the time that samples are received from local and provincial laboratories. The lack of timely availability of WGS results means that links between isolates are usually discovered too late to be of practical relevance. It was also reported that communication between the genomics team and the epidemiological team at INEI-ANLIS, as well as with the provincial public health authorities was insufficient for effective use of the additional information provided by WGS for outbreak response.
Research and methods applied	▸ The institution reported significant positive effects related to the better understanding of disease transmission and the development of better diagnostic tests. However, it did not report any effects regarding an improvement in epidemiological methods so far.
Effects on wider society	▸ INEI-ANLIS did not identify any significant effects on the wider society. It indicated that the nature of the surveillance system, gaps in communication between different units and institutions, and a lack of implementation of public health measures in response to the available data have limited the potential impact of WGS for reducing the negative effects of outbreaks for the wider society.
c) Negative effects of using WGS	
Negative effects of using WGS	INEI-ANLIS did not identify any negative effects of using WGS.
**VI. Outlook**	
Balance of costs and benefits achieved	▸ On balance, the benefits of using WGS outweigh the costs, given the improvements in the accuracy of results and turnaround time (for the full analysis). With the appropriate capacity-building, WGS also brings different actors of public health together.
Potential for cost reductions of using WGS for pathogen identification and	▸ Advances in sequencing technology and increasingly automated analysis of sequencing results are expected to drive further cost reductions in using WGS for pathogen identification and surveillance.

surveillance in the future (through e.g. techno-logical advances)	▸ INEI-ANLIS considered that the cross-pathogen potential of WGS was one of the most important areas of potential cost reduction. It pointed out that at the present time, INEI-ANLIS already had a genomic platform for all pathogens in their institute with equipment, reagent and personnel costs all centralised.
Future opportunities and challenges	▸ A key challenge identified affecting present and future use of WGS is the high cost of consumables, which are significantly more expensive than in other countries, such as the US or the UK. This is aggravated by exchange rate fluctuations and import duties, which make it very difficult for INEI-ANLIS to reliably purchase consumables for conducting WGS on a routine basis. It will be difficult to fully switch to WGS as long as this reliability and affordability of supplies is not ensured (either through changes in the pricing policies of producers and distributors of consumables, or through agreements with international organisations to ensure regular supply).

**VII. Key sources/references**	
Cost questionnaire	Cost questionnaire completed by INEI-ANLIS
Preparatory phone interview	a) Background information and description of activities
Case study visit and follow up	b) Additional data and clarifications provided
Scientific literature	c) Baker, K. S., J. Campos, M. Pichel, A. Della Gaspera, F. Duarte-Martínez, E. Campos-Chacón, H. M. Bolaños-Acuña, et al. 2017. "Whole Genome Sequencing of Shigella Sonnei through PulseNet Latin America and Caribbean: Advancing Global Surveillance of Foodborne Illnesses." Clinical Microbiology and Infection 23 (11): 845–53. doi:10.1016/j.cmi.2017.03.021.<br>d) Chinen, Isabel, Marcelo Galas, Ezequiel Tuduri, Maria Rosa Vinas, Carolina Carbonari, Anabella Della Gaspera, Daniela Napoli, et al. 2016. "Whole Genome Sequencing Identifies Independent Outbreaks of Shigellosis in 2010 and 2011 in La Pampa Province, Argentina." *BioRxiv*. doi:10.1101/049940.<br>e) World Health Organisation (WHO). 2018. "Implementing Whole Genome Sequencing to Support Public Health Surveillance in Argentina."<br>f) World Health Organization (WHO). 2018. "Annex 1. Contribution/Implementation of Whole Genome Sequencing to the National Surveillance of the Shiga Toxin Producing E. Coli O157:H7 in Argentina." WHO Pilot Project.<br>g) World Health Organization (WHO). 2018. "Annex 2. Contribution of Whole Genome Sequencing to the National Surveillance of Shigella Sonnei in Argentina Introduction." WHO Pilot Project.<br>h) World Health Organization (WHO). 2018. "Annex 3. Contribution/ Implementation of Whole Genome Sequencing to the National and International Surveillance of Salmonella Spp." WHO Pilot Project.
Other	i) Website, ANLIS http://www.anlis.gov.ar/

4.6. Maryland Department of Health (MDH)

<table>
<tr><th colspan="4">Foodborne pathogen surveillance – Maryland Department of Health, USA</th></tr>
<tr><td colspan="4">I. Institution</td></tr>
<tr><td>Name of institution</td><td colspan="3">Maryland Department of Health (MDH)</td></tr>
<tr><td>Type of institution</td><td colspan="3">State department for public health</td></tr>
<tr><td>Description[h)]</td><td colspan="3">The Maryland Department of Health (MDH) is the public health department of the US state of Maryland. It is responsible for dealing with communicable diseases, tainted foods, and dangerous products. The Laboratories Administration of MDH provides diagnostic and reference services to Maryland hospitals, as well as support to local health departments. Environmental testing is also conducted. The Laboratories Administration consists of a Central Laboratory in Baltimore and Regional Laboratories in Cumberland and Salisbury. The public health laboratories perform over 10 million laboratory tests annually on human specimens and environmental samples submitted by county health departments and clinics, private physicians, hospitals, correctional facilities, private medical laboratories, and the Maryland Department of the Environment.</td></tr>
<tr><td>Location</td><td colspan="3">Maryland, USA</td></tr>
<tr><td colspan="4">II. Surveillance activities covered by case study</td></tr>
<tr><td>Activity</td><td colspan="3">Routine laboratory surveillance</td></tr>
<tr><td>Reference period</td><td colspan="3">01/2017 – 12/2017</td></tr>
<tr><td>Pathogen(s) covered</td><td colspan="3">Salmonella spp., E. Coli, Shigella spp., Campylobacter spp., Vibrio spp., Listeria</td></tr>
<tr><td>Summary of routine surveillance activities using WGS</td><td colspan="3">Since 2013, the MDH Laboratories Administration has routinely utilised WGS to sequence infectious agents recovered from clinical specimens and environmental samples that are submitted to the public health laboratory as part of state-wide public health infectious disease surveillance programs or as part of outbreak/case investigations.</td></tr>
<tr><td>Type of sample</td><td colspan="3">Isolates</td></tr>
<tr><td>Region covered by laboratory surveillance</td><td colspan="3">Maryland</td></tr>
<tr><td rowspan="7">Number of samples analysed in reference period</td><td>Pathogen</td><td>Samples analysed by conventional methods</td><td>Samples sequenced using WGS</td></tr>
<tr><td>Salmonella spp.</td><td rowspan="6">The cost calculation is based on experiences with the listed conventional methods, assuming the same number of samples as with WGS</td><td>1010</td></tr>
<tr><td>E. coli</td><td>81</td></tr>
<tr><td>Shigella spp.</td><td>134</td></tr>
<tr><td>Campylobacter spp.</td><td>504</td></tr>
<tr><td>Vibrio spp.</td><td>38</td></tr>
<tr><td>Listeria spp.</td><td>35</td></tr>
<tr><td>Conventional methods used as reference for costing</td><td colspan="3">▸ Salmonella spp.: PFGE (100% of samples)
▸ Shigella spp.: PFGE (100%)
▸ E. coli: PFGE (100%), Real-Time PCR (100%)
▸ Campylobacter spp.: PFGE (100%), MALDI-TOF (100%)
▸ Vibrio spp.: PFGE (100%), Real-Time PCR (100%)
▸ Listeria: PFGE (100%)</td></tr>
</table>

	MDH reported that a two-stage approach was used for analysis of isolates during the case study period. The isolates were first analysed in another unit applying standard methods (e.g. serotyping). In the second stage, the isolates were analysed in parallel using PFGE (plus PCR and MALDI-TOF for certain pathogens) and WGS. The differential costs of the first-stage tests therefore net to zero. However, MDH indicated that it plans to switch fully to WGS in 2019 and do away with the first-stage microbiology tests. This will lead to additional cost savings which are not captured by this case study.
**Sample preparation WGS**	DNA extraction is automated and performed by the Core Sequencing group using the Roche MagNA Pure 24 platform. Library preparation is completed manually with Illumina Nextera XT kits.
**Sequencer used for WGS**	MiSeq (Illumina)
**Batch size for WGS analysis**	Batch size ranged between 16 and 32, with an average batch size of 24 for automated DNA extraction and for library preparation and sequencing.
**Reference dataset used for WGS**	No in-house reference dataset. During the case study period, MDH used CLC genomics software for denovo assembly and the Center for Genomics Epidemiology (CGE) website for sequencing analysis (http://www.genomicepidemiology.org), which is hosted by DTU, one of the project leaders of the COMPARE project.<br>Sequences are uploaded to national and international databases maintained by the FDA (GenomeTrakr) or CDC (PulseNet) and phylogenetic analysis (e.g. cgMLST, wgMLST or SNP analysis) of the generated sequences is used to recognise genetically related clusters of bacterial isolates.

III. Detailed overview of costs of WGS and conventional methods

In the following, all costs are provided on a per-sample basis. Equipment costs are annualised and incorporate the annual maintenance costs as reported by the institution. They are adjusted for the percentage use of the equipment for the listed pathogens samples during the reference period (i.e. if a sequencer was also used for other purposes, this is taken into account). Consumables costs are adjusted for the failure rate (i.e. the percentage of consumables wasted, e.g. due to failed runs). Staff time is provided in terms of the minutes of hands-on staff time per sample, for both professionals and technicians. For the calculation of total costs, staff time is then monetised based on labour cost data provided by the institution, plus a 25% surcharge for overheads. For comparison purposes only, we have also provided staff costs monetised based on EU average labour costs. More detailed cost data is provided in Annex I.

a) Costs of using WGS

Sample preparation and sequencing	*Cost type*	*Cost per sample*
	Equipment costs	€ 28.01
	Consumables	€ 104.40
	Other costs	€ 0
	Staff time professionals	*14 minutes*
	Staff time technicians	*0 minutes*
	Staff costs, monetised based on labour cost data for the US (in brackets: based on labour cost data for the EU as a whole)	€ 9.85 (€ 10.57)
	**Total**	**€ 142.26**

Bioinformatics and other analyses	*Cost type*	*Cost per sample*
	Equipment costs	€ 1.52

	Other costs	€ 0
	Staff time professionals	*15 minutes*
	Staff time technicians	*0 minutes*
	Staff costs, based on labour cost data for the US (for EU)	€ 10.73 (€ 11.51)
	**Total**	**€ 12.25**
b) Costs of conventional methods		
Method A: PFGE	*Cost type*	*Cost per sample*
	Equipment costs	€ 4.18
	Consumables	€ 31.15
	Other costs	€ 0
	Staff time professionals	*58 minutes*
	Staff time technicians	*0 minutes*
	Staff costs, based on labour cost data for the US (for EU)	€ 40.65 (€ 43.62)
	**Total**	**€ 75.97**
Method B: Real-Time PCR	*Cost type*	*Cost per sample*
	Equipment costs	€ 9.55
	Consumables	€ 12.55
	Other costs	€ 0
	Staff time professionals	*30 minutes*
	Staff time technicians	*0 minutes*
	Staff costs, based on labour cost data for the US (for EU)	€ 21.02 (€ 22.56)
	**Total**	**€ 43.13**
Method C: MALDI-TOF	*Cost type*	*Cost per sample*
	Equipment costs	€ 3.69
	Consumables	€ 3.25
	Other costs	€ 0
	Staff time professionals	*2 minutes*
	Staff time technicians	*0 minutes*
	Staff costs, based on labour cost data for the US (for EU)	€ 1.40 (€ 1.50)
	**Total**	**€ 8.35**

IV. Costs of using WGS compared to the costs of conventional methods

The following comparison of costs per sample using WGS compared to the costs of conventional methods considers that the number of samples processed differed for the different conventional methods. The weighted cost of the conventional methods provided here is therefore a weighted figure which accounts for the use rate of the various methods across the different pathogens. See Annex I for more details.

Comparison of equipment costs

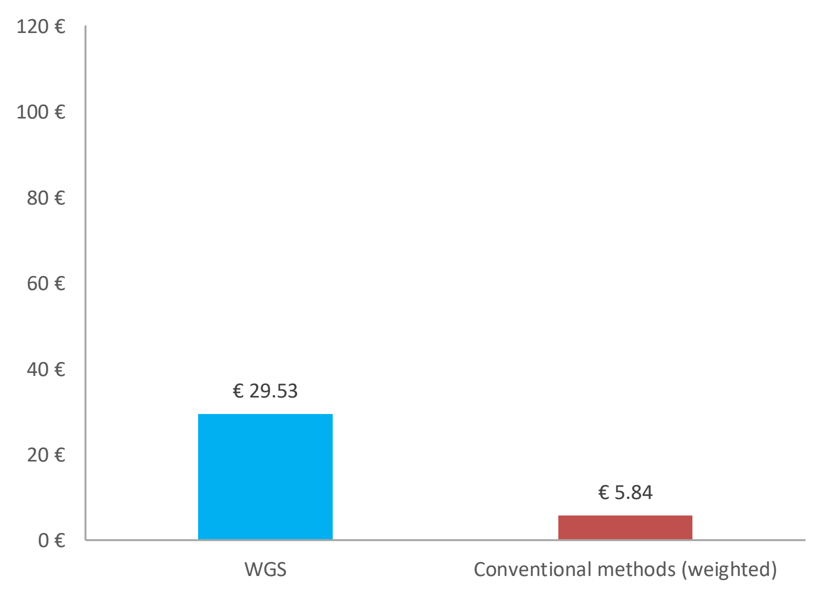

Equipment costs per sample are higher for WGS than for the weighted average of conventional methods (€ 29.53 vs € 5.84). However, the total purchase costs of all equipment for conventional methods (approx. € 302 000) exceeds the total purchase costs of equipment for WGS (approx. € 209 000), indicating that the cost difference is rather due to the lower use rates (5-9%) for some of the conventional equipment. The most expensive equipment cost elements for WGS are the two Illumina MiSeq sequencers, which cost a combined total of € 155 624.

Comparison of costs of consumables

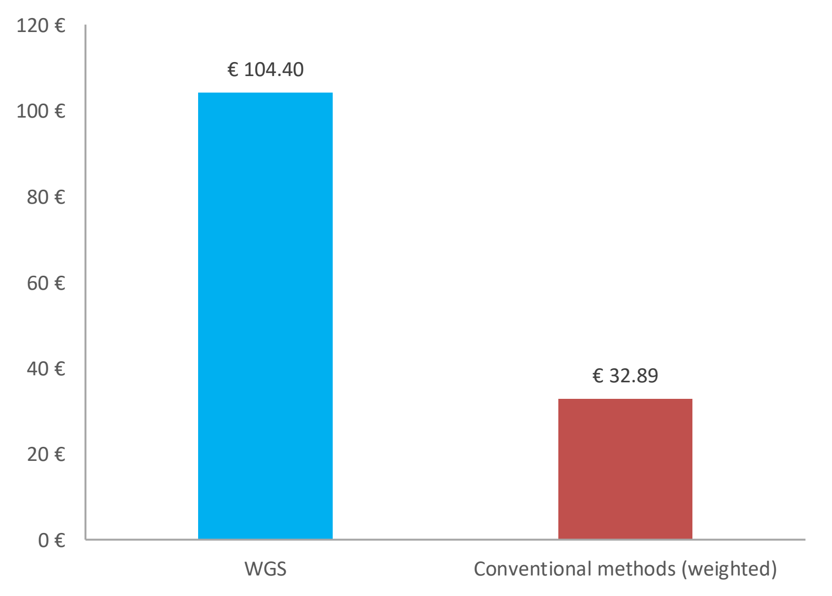

The cost of consumables for WGS is more than three times the cost of consumables for conventional methods (€ 104.40 vs € 32.89), and is also the most expensive cost type for WGS overall. The most expensive consumables cost elements for WGS are the sequencing kits used for the Illumina MiSeq, with per-sample costs ranging from € 20.20 to € 28.56.

Comparison of staff time used (in minutes)

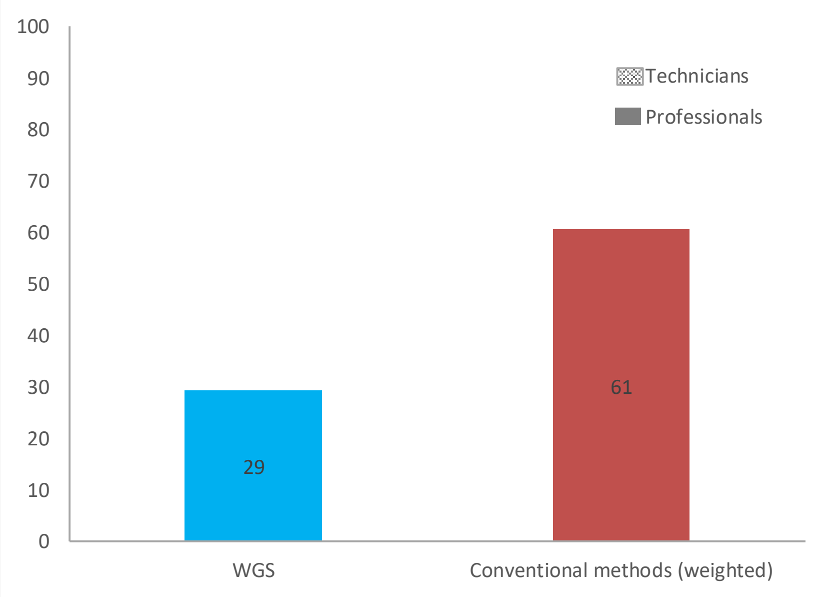

Staff time required for WGS analysis is less than half the staff time required for conventional methods (29 minutes vs 61 minutes). No technician time was reported for either WGS or conventional methods, as no staff member of the MDH lab falls under the case study definition for the 'technician' category. When monetised on the basis of average labour costs, staff costs are considerably cheaper for WGS than for conventional methods (€ 20.58 vs € 42.43).

Comparison of overall costs

Cost type	*Cost per sample (WGS)*	*Cost per sample (conventional methods)*
Equipment costs	€ 29.53	€ 5.84
Consumables	€ 104.40	€ 32.89
Other costs	€ 0	€ 0
Staff time professionals	*29 minutes*	*61 minutes*
Staff time technicians	*0 minutes*	*0 minutes*
Staff costs, based on labour cost data for the US (for EU)	€ 20.58 (€ 22.09)	€ 42.43 (€ 45.53)
**Total**	**€ 154.51**	**€ 81.16**

Differential costs

The cost difference between WGS and conventional methods is € 73.35 per sample. A sample analysed with WGS costs approximately twice the amount of an analysis using conventional methods. As indicated in the figures above, this cost difference is due to equipment and consumables, as staff costs are in fact lower for WGS than for conventional methods.

V. Effects of using WGS results

a) Turnaround time. Turnaround time is defined as the usual number of days of work from receipt and opening of an incoming sample until the reporting of results. Turnaround time does not include weekends and holidays, except in case that work has been conducted on these days, e.g. for a sequencing run or other analyses.

Turnaround time

- The turnaround time for WGS analysis from the time the isolate is received by the Sequencing laboratory to the sharing of WGS results with the CDC and FDA takes 7 working days.

	• For PFGE, the time between receiving an isolate by the PFGE laboratory and uploading the PFGE pattern to the national database is about 4 working days. All *E. coli* and Listeria are processed within a 4-day turnaround time. The turnaround time for other non-priority routine surveillance organisms such as Salmonella and Campylobacter varies from 4-10 work days or even longer depending upon situational factors such as sample load, work priorities, repeats, etc.
**b) Positive effects of using WGS for pathogen identification and surveillance during the reference period**	
Sampling and sampling strategies	• Effects on sampling and sampling strategies were considered to be not applicable to MDH, as it receives clinical isolates from partners.
Analytical results and processes	• MDH observed very significant effects of WGS with respect to the improved accuracy, sensitivity, specificity and detail of the results produced. Retrospective analyses of past outbreaks with WGS conducted by MDH, e.g. related to Vibrio outbreaks in 2010[d)] and in 2012-13,[e-f)] also demonstrate the value of the higher-resolution data provided by WGS and show how this data can provide new analytical insights (e.g. differentiating between west coast and east coast strains of the same sequence type). The higher-quality data available through WGS has also helped to identify emerging threats to public health, e.g. by allowing public health authorities to identify new sequence types that are becoming more prevalent, as was the case with ST361 *Vibrio parahaemolyticus* in 2016.[g)]<br>• MDH considered that there had been a significant effect of WGS on the simplification of laboratory workflows. In particular, it reported that specific instruments settings, methods, and or protocols are needed for PFGE that are organism specific, while this is not the case for WGS.<br>• No positive effects were reported regarding reductions in time needed for the analysis, in consumables needed for the analysis, or in overall costs of the analysis. MDH reported that WGS in fact takes more time than PFGE and is more expensive.
Outbreak identification and response	• Significant or very significant effects were reported with respect to improved detection that outbreaks are related, improved information on outbreak epidemiology, and a reduction in the duration of an outbreak. MDH reported that these benefits had been particularly well-observed in a multi-state outbreak of Salmonella in Mexican papayas in 2017.[c)] Based on the information provided by WGS, the product was pulled from the market, leading MDH to consider that there had also potentially been a slightly positive effect on the disease burden.<br>• With respect to the earlier detection of an outbreak, MDH reported that both PFGE and WGS were carried out in parallel during this period, so this effect was not applicable.<br>• MDH reported that it had no regulatory authority for imposing control measures, and therefore indicated that effects on improved information for imposing additional control or biosecurity measures were not applicable.<br>• No effects were reported by MDH with respect to a reduction in overall costs for outbreak identification and response.
Research and methods applied	• MDH reported significant effects of WGS regarding a better understanding of disease transmission due to the additional information provided.<br>• No effects were reported by MDH regarding an improvement in epidemiological methods or other research benefits. Effects related to the development of better diagnostic tests were considered non-applicable.
Effects on wider society	• MDH indicated that it was not able to provide assessments of the concrete effects of WGS on the wider society during the case study period.

c) Negative effects of using WGS	
Negative effects of using WGS	No negative effects of WGS were reported during the case study period.
**VI. Outlook**	
Balance of costs and benefits achieved	MDH indicated that WGS was still on the whole more expensive than conventional methods, which is confirmed by the case study results. However, the cost difference is expected to be reduced once the application of standard conventional methods (e.g. serotyping) during the first stage of the analysis (not covered by this case study, see above) is discontinued (as it becomes redundant due to WGS).
Potential for cost reductions	MDH considered that costs might come down as WGS technologies are more widely utilized for national or international laboratory surveillance, but did not believe that these cost reductions would be significant in the near future.
Future opportunities and challenges	MDH indicated that the CDC and FDA are currently moving the workflow of WGS analysis to BioNumerics and transitioning pathogen surveillance from using PFGE to WGS. As a result, a national database of genomic data will be available as a data source to all State Public Health Laboratories including MDH to analyse and determine pathogen clusters for outbreaks. While this will help to identify outbreaks more effectively, this could put an extra burden on state public health laboratories through the need to re-train the workforce and add or change existing infrastructure.
**VII. Key sources/references**	
Cost questionnaire	Cost questionnaire completed by Maryland Department of Health
Preparatory phone interview	a) Background information and description of activities
Case study visit and follow up	b) Additional data and clarifications provided
Scientific literature	c) Centers for Disease Control and Prevention (2017) Multistate Outbreak of Salmonella Infections Linked to Imported Maradol Papayas (Final Update). https://www.cdc.gov/salmonella/kiambu-07-17/index.html<br>d) Haendiges, J. et al. (2016) 'A Nonautochthonous U.S. Strain of Vibrio parahaemolyticus Isolated from Chesapeake Bay Oysters Caused the Outbreak in Maryland in 2010', Applied and Environmental Microbiology, 82(11), pp. 3208–3216. doi: 10.1128/aem.00096-16.<br>e) Haendiges, J. et al. (2014) 'Pandemic Vibrio parahaemolyticus, Maryland, USA, 2012', Emerging Infectious Diseases, 20(4), pp. 718–720.<br>f) Haendiges, J. et al. (2015) 'Characterization of Vibrio parahaemolyticus clinical strains from Maryland (2012-2013) and comparisons to a locally and globally diverse V. parahaemolyticus strains by whole-genome sequence analysis', Frontiers in Microbiology, 6(FEB), pp. 1–11. doi: 10.3389/fmicb.2015.00125.<br>g) Xu, F. et al. (2017) 'Sequence Type 631 Vibrio parahaemolyticus, an Emerging Foodborne Pathogen in North America', Journal of Clinical Microbiology, 55(2), pp. 645–648.
Other	h) Maryland Department of Health website, https://health.maryland.gov/laboratories/Pages/-About-The-Labs.aspx

4.7. Public Health Agency Canada (PHAC)

Foodborne pathogen surveillance – Public Health Agency of Canada			
**I. Institution**			
**Name of institution**	Public Health Agency of Canada / Agence de la santé publique du Canada		
**Type of institution**	Federal agency for public health		
**Description**[e-i]	The Public Health Agency of Canada (PHAC) is a federal agency with the mandate to promote health; prevent and control chronic diseases, injuries, and infectious diseases; prepare for and respond to public health emergencies; and strengthen intergovernmental collaboration on public health. PHAC's National Microbiology Laboratory conducts research and lab-based surveillance as well as coordinate emergency preparedness and response activities in the area of public health. The National Microbiology Laboratory is also responsible for coordinating PulseNet Canada, the national surveillance system for foodborne disease outbreaks.		
**Location**	Winnipeg, Manitoba, Canada		
**II. Surveillance activities covered by case study**			
**Activity**	Routine laboratory surveillance		
**Reference period**	05/2017 – 05/2018		
**Pathogen(s) covered**	Salmonella, Listeria		
**Summary of routine surveillance activities using WGS**	All cases of listeriosis in Canada have been characterised by WGS since February 2017, as have all cases of salmonellosis beginning in May 2017. Prior to this, WGS had been used since approximately 2014 to supplement traditional methods, but only during outbreak response for E. coli, Salmonella, and Listeria. All Listeria and Salmonella isolates from food products (as part of PHAC's integrated/targeted sampling and from its food regulatory partners) were also characterized by WGS and included within national surveillance system during the reference period.<br>During the reference period, as part of a transitional arrangement for the implementation of WGS, all samples were collected by laboratories in Canada's ten provinces and then shipped to the National Microbiology Lab for centralised sequencing. PHAC indicated that this workflow was only temporary, and that the larger provinces would soon do their own sequencing as part of a decentralised surveillance model.		
**Type of sample**	Isolates		
**Region covered by laboratory surveillance**	Canada (all provinces and territories)		
**Number of samples analysed in reference period**	*Pathogen*	*Samples analysed by conventional methods*	*Samples sequenced using WGS*
	Salmonella	The cost calculation is based on experiences with the listed conventional methods, assuming the same number of samples as with WGS	8 273
	Listeria		357
**Conventional methods used as reference for costing**	▸ Salmonella: Biochemical testing (100% of samples), Serotyping (100%), PFGE (65%)<br>▸ Listeria: Biochemical testing (100%), PFGE (100%)<br>▸ Conventional methods were carried out in parallel to characterisation using WGS during the reference period. Since then, conventional testing has been		

	largely discontinued, and is now carried out on less than 10% of isolates.
**Sample preparation WGS**	▸ PHAC follows the standard PulseNet International procedures for the use of WGS on Salmonella and Listeria.
**Sequencer used for WGS**	▸ MiSeq (Illumina)
**Batch size for WGS analysis**	▸ Average batch size of 32 isolates
**Reference dataset used for WGS**	▸ PHAC uses reference datasets from NCBI and from the shared schemes through PulseNet International. It has developed its own highly innovative bioinformatics infrastructure with custom pipelines and a custom bioinformatics platform, Integrated Rapid Infectious Disease Analysis (IRIDA), which is entirely open-source, automatic, pathogen-neutral and adapted for a cross-pathogen approach.[f)]

III. Detailed overview of costs of WGS and conventional methods

In the following, all costs are provided on a per-sample basis. Equipment costs are annualised and incorporate the annual maintenance costs as reported by the institution. They are adjusted for the percentage use of the equipment for the listed pathogens samples during the reference period (i.e. if a sequencer was also used for other purposes, this is taken into account). Consumables costs are adjusted for the failure rate (i.e. the percentage of consumables wasted, e.g. due to failed runs). Staff time is provided in terms of the minutes of hands-on staff time per sample, for both professionals and technicians. For the calculation of total costs, staff time is then monetised based on labour cost data provided by the institution, plus a 25% surcharge for overheads. For comparison purposes only, we have also provided staff costs monetised based on EU average labour costs. More detailed cost data is provided in Annex I.

a) Costs of using WGS

Sample preparation and sequencing	*Cost type*	*Cost per sample*
	Equipment costs	€ 7.32
	Consumables	€ 69.75
	Other costs	€ 0.00
	Staff time professionals	*0 minutes*
	Staff time technicians	*19 minutes*
	Staff costs, monetised based on labour cost data for Canada (in brackets: based on labour cost data for the EU)	€ 7.89 (€ 7.85)
	**Total**	**€ 84.95**
Bioinformatics and other analyses	*Cost type*	*Cost per sample*
	Equipment costs	€ 68.59
	Other costs	€ 0.00
	Staff time professionals	*90 minutes*[22]
	Staff time technicians	*0 minutes*
	Staff costs, based on labour cost data for Canada (for EU)	€ 61.82 (€ 67.99)

[22] Note that staff time for bioinformatics includes IT support that relates exclusively for maintenance of the database that is used for routine surveillance activities, as well as analytical time for genomic epidemiology.

	**Total**	**€ 130.41**
b) Costs of conventional methods		
Method A: Biochemical testing (100% of Salmonella samples, 100% of Listeria samples)	*Cost type*	*Cost per sample*
	Equipment costs	€ 0.00
	Consumables	€ 2.42
	Other costs	€ 0.00
	Staff time professionals	*0 minutes*
	Staff time technicians	*40 minutes*
	Staff costs, based on labour cost data for Canada (for EU)	€ 16.41 (€ 16.33)
	**Total**	**€ 18.83**
Method B: Serotyping (100% of Salmonella samples)	*Cost type*	*Cost per sample*
	Equipment costs	€ 0.00
	Consumables	€ 5.12
	Other costs	€ 0.00
	Staff time professionals	*0 minutes*
	Staff time technicians	*40 minutes*
	Staff costs, based on labour cost data for Canada (for EU)	€ 16.41 (€ 16.33)
	**Total**	**€ 21.53**
Method C: PFGE (65% of Salmonella samples, 100% of Listeria samples)	*Cost type*	*Cost per sample*
	Equipment costs	€ 18.51
	Consumables	€ 41.58
	Other costs	€ 0.00
	Staff time professionals	*15 minutes*
	Staff time technicians	*30 minutes*
	Staff costs, based on labour cost data for Canada (for EU)	€ 22.42 (€ 23.38)
	**Total**	**€ 82.51**

IV. Costs of using WGS compared to the costs of conventional methods

The following comparison of costs per sample using WGS compared to the costs of conventional methods considers that the number of samples processed differed for the different conventional methods. The weighted cost of the conventional methods provided here is therefore a weighted figure which accounts for the use rate of the various methods across the different pathogens. See Annex I for more details.

Comparison of equipment costs

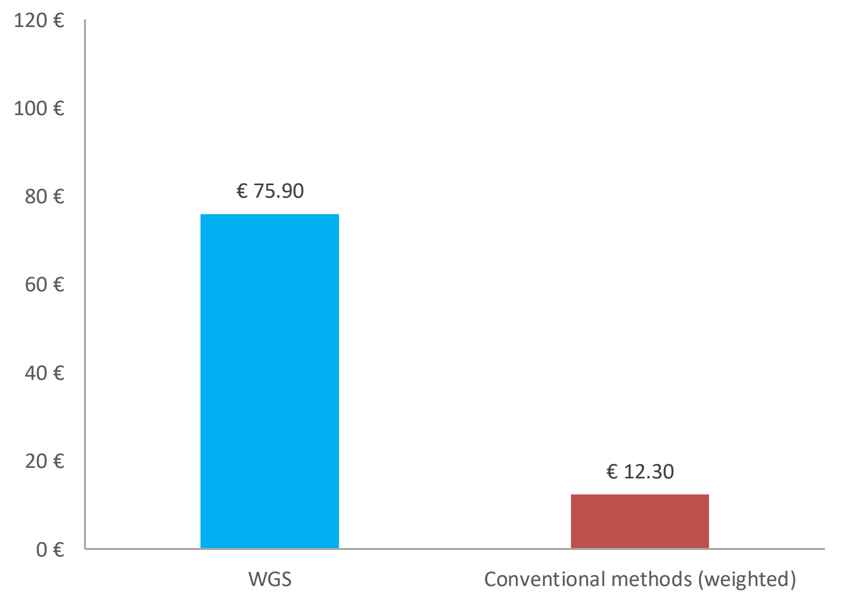

Equipment costs per sample are considerably higher for WGS than for the weighted average of conventional methods (€ 75.90 vs € 12.30). The most expensive equipment cost elements for WGS are not the sequencers themselves (three Illumina MiSeq sequencers, costing a combined total of € 264 345), but the bioinformatics infrastructure, which costs a total of € 2.9 million for the necessary high performance computing hardware (storage, network and servers) and BioNumerics software licences.

Comparison of costs of consumables

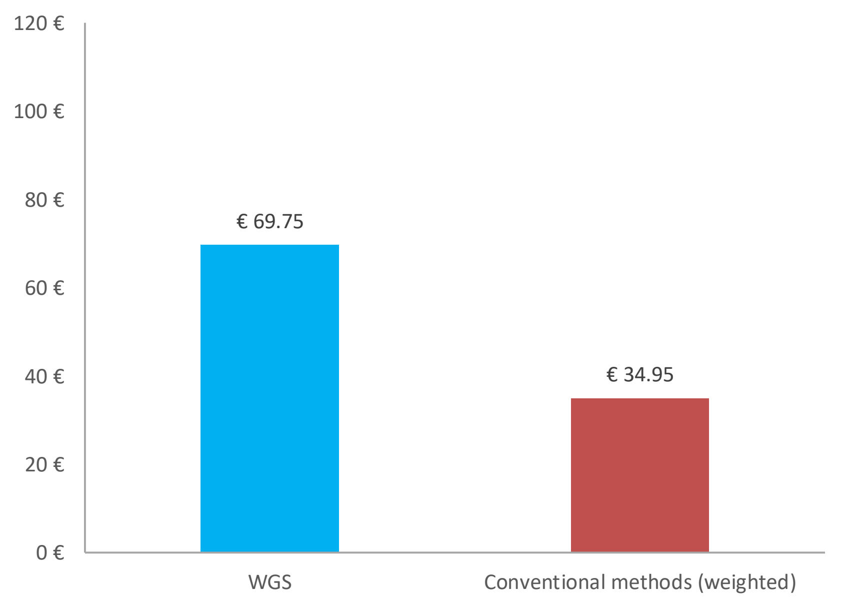

The cost of consumables for WGS is more expensive than the cost of consumables for conventional methods (€ 69.75 vs € 34.95). The most expensive consumables cost elements for WGS are the sequencing kits used for the Illumina MiSeq, costing € 33.60 per sample. The cost of conventional methods is largely driven by the consumables costs for PFGE (€ 41.58 per sample), which are significantly higher than consumables costs for either biochemical testing (€ 2.42 per sample) or serotyping (€ 5.12).

Comparison of staff time used (in minutes)

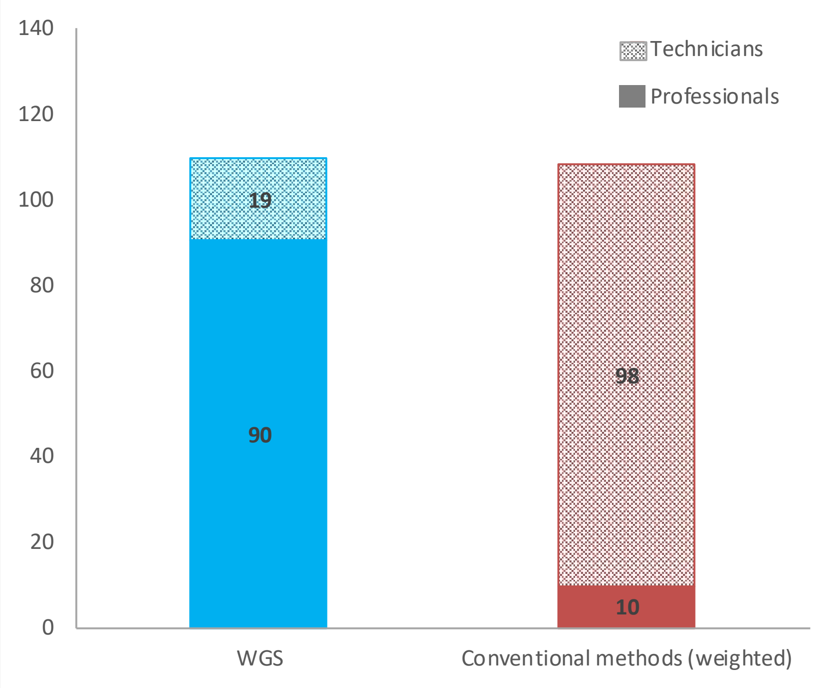

Total staff time in minutes is roughly equal between WGS and conventional methods (a total of 110 minutes per sample versus a total of 108 minutes per sample). While the staff time for WGS largely consists of professional staff time (which all takes place at the bioinformatics stage), the staff time for conventional methods consists almost entirely of technician staff time. When monetised on the basis of average labour costs, staff time is more expensive for WGS than for conventional methods (€ 69.71 vs € 47.05).

Comparison of overall costs

Cost type	*Cost per sample (WGS)*	*Cost per sample (conventional methods)*
Equipment costs	€ 75.90	€ 12.30
Consumables	€ 69.75	€ 34.95
Other costs	€ 0.00	€ 0.00
Staff time professionals	*90.4 minutes*	*9.8 minutes*
Staff time technicians	*19.2 minutes*	*108.1 minutes*
Staff costs, based on labour cost data for Canada	€ 69.71	€ 47.05
**Total**	**€ 215.36**	**€ 94.29**

Differential costs

The cost difference between WGS and conventional methods is € 121.07 per sample. A sample analysed with WGS costs approximately 128% more than an analysis using conventional methods. As indicated in the figures above, WGS is more expensive than conventional methods for all cost types.

V. Effects of using WGS results

a) Turnaround time. Turnaround time is defined as the usual number of days of work from receipt and opening of an incoming sample until the reporting of results. Turnaround time does not include weekends and holidays, except in case that work has been conducted on these days, e.g. for a sequencing run or other analyses.

Turnaround time

- The turnaround time for WGS analysis from the time the isolate is received

	to the reporting of results is 10-14 days, with potentially an additional 5-21 days for shipping time.<br>- For conventional methods, the turnaround time is 1.5-5 days of work.<br>The higher turnaround time for WGS is primarily due to the batching required (i.e. waiting to accumulate enough samples to run the sequencers cost-efficiently) as well as the time to ship isolates from provincial labs across Canada to the central laboratory in Winnipeg for sequencing. The shipping time is unique to the WGS transition period and was not relevant for conventional methods, as conventional methods were previously done entirely at the provincial level. PHAC indicated that the turnaround time for WGS would likely be faster than for conventional methods once the transition to more decentralised model (i.e. with sequencing done in individual provinces) was complete.<br>The difference in turnaround time in the transition to WGS is 'extremely relevant' for PHAC. All of its provincial and federal laboratory partners who rely on the surveillance data generated by PHAC have had to adjust their own workflows as a result, which was reported to be quite disruptive. The delay in turnaround time with WGS has increased concern that the recall of information when patients are questioned on their food histories may be compromised, as well as concern that outbreaks may be detected slower.
b) Positive effects of using WGS for pathogen identification and surveillance during the reference period	
Sampling and sampling strategies	- No effects on sampling and sampling strategies were observed by PHAC.
Analytical results and processes	- PHAC observed very significant effects of WGS with respect to the improved accuracy, sensitivity, specificity and detail of the results produced.<br>- PHAC considered that there had been a significant effect of WGS on the simplification of laboratory workflows. In particular, it reported that a reduction in the number of different tests had simplified workflows. At the same time, however, it indicated that the replacement workflow with WGS is significantly more complex with respect to coordination. This is due to the integration of more units in the workflow, as traditional methods had previously been handled exclusively by the enterics lab, while the WGS workflow now spans the enterics lab, the genomics core lab, and the bioinformatics section.<br>- With respect to the time needed for the analysis, PHAC reported a very significant negative effect (see turnaround time above). It also reported no effect of WGS on staff time.<br>- PHAC reported a significant effect of WGS in terms of the reduction of overall costs for the analysis, as well as a moderate effect of WGS in terms of a reduction in consumables needed for the analysis. It indicated that WGS enabled PHAC to discontinue expensive tests like PFGE and serotyping; however, these were only discontinued after the reference period.
Outbreak identification and response	- Significant or very significant effects were reported with respect to improved detection that outbreaks are related, improved information on outbreak epidemiology, and improved information for imposing additional control or biosecurity measures. PHAC reported, for example, that PFGE had been detecting Listeria outbreaks were none existed, diverting epidemiological resources to investigating outbreaks that were not real. PHAC noted that WGS therefore allowed them to devote more resources to investigating 'true clusters'.<br>- PHAC reported that within a few weeks of implementing WGS for Salmonella surveillance, it began to detect outbreaks of S. Enteritidis that were not discernible by PFGE. Overall, the number of S. Enteritidis outbreaks detected with laboratory data in Canada increased from less than 20 each year in 2012-2016 to more than 100 in 2017, the first year that WGS was

	introduced for routine use.<br>▸ Within the first 6 months of using WGS, 14 different outbreaks of S. Enteritidis were detected and solved, and led to recalls of various types of chicken products. Utilising all of the WGS data allowed PHAC to estimate the burden of illness from the products overall, and this led to a national food policy change. For example, data from WGS detected multiple S. Enteritidis outbreaks linked to raw frozen breaded chicken products, which were estimated to comprise approximately 40% of the disease burden of S. Enteritidis each year. On the basis of this evidence, the Government of Canada adopted much stricter regulations for producers of raw frozen breaded chicken products in 2018.[i)]<br>▸ Nevertheless, PHAC reported that it did not have specific information regarding effects of WGS on a reduction in the duration of an outbreak, reductions in the disease burdens for humans or animals, or a reduction in the overall costs for outbreak identification and response. Although PHAC could point to cases where WGS had made a difference (such as with Salmonella-contaminated chicken products), it indicated that it had not yet undertaken a 'before' and 'after' measurement in this respect.<br>▸ With respect to the earlier detection of an outbreak, PHAC reported a negative effect of WGS due to the lengthening of the turnaround time.
Research and methods applied	▸ PHAC reported very significant effects of WGS regarding a better understanding of disease transmission, as well as moderate effects on the development of better diagnostic tests. It indicated that WGS offers an unprecedented level of potential research questions that may help to mitigate future disease burdens.<br>▸ Very significant effects were reported in terms of other benefits for research. In particular, PHAC reported that data generated through WGS were being used in research for scheme development, genome-wide association studies, machine learning, and antimicrobial resistance.<br>▸ PHAC also indicated that there was a very significant positive effect as the infrastructure and protocols developed for WGS in the context of foodborne disease were being leveraged for other disease areas.
Effects on wider society	▸ PHAC reported moderate effects of WGS in reducing negative effects of outbreaks on consumer trust in food.<br>▸ No other effects on the wider society were reported. PHAC indicated that there was likely a positive effect with respect to reducing the costs of outbreaks for the wider society, but that this had not yet been measured.
c) Negative effects of using WGS	
Negative effects of using WGS	▸ The increase in turnaround time (above) was considered to be one of the most significant negative effects of using WGS, since provincial laboratories could not afford to add WGS to their services and were therefore required to ship all their samples to the National Microbiology Laboratory.<br>▸ Transition costs from the former PFGE-based system to a WGS-based system were reported to be considerable, since conventional methods and WGS were temporarily performed in parallel. PHAC also indicated that there were many challenges in knowledge translation so that all laboratories and epidemiologists across the country could use the results from WGS for public health and regulatory decision-making. This put a significant (cost) burden on PHAC to provide extensive and ongoing training around the country.
**VI. Outlook**	
Balance of costs and	▸ Despite the challenges of longer turnaround times and the transition cost,

benefits achieved	PHAC reported that the use of WGS as the primary surveillance method is widely supported in Canada due to the significant improvement in the accuracy of the data and the actions that are taken from it. It reported that the use of WGS data has significantly increased confidence in taking action (regulatory or otherwise).
Potential for cost reductions	▸ PHAC indicated that their current focus was on reducing turnaround time while keeping costs manageable, and that shorter read kits, the ability to sequence in smaller batches in the provincial labs, and (assumed future) shorter run times on the sequencers themselves would ideally contribute towards this goal.<br>▸ PHAC indicated that since most laboratories (and their purchasing of reagents) work on a pathogen/organism basis, there is still work to be done to realise maximum cost efficiencies through WGS.
Future opportunities and challenges	▸ PHAC considered that the information provided by WGS has the potential for substantial impacts of surveillance, outbreak detection and response, and is poised to mitigate the burden of foodborne disease with international cooperation. It considered that metagenomics was a promising area of future research. Finally, PHAC considered that with WGS technology, it was now possible to make the One Health approach a reality.<br>▸ However, PHAC reported that in practice, WGS is a 'severe disruption' to existing public health systems and implementation is very challenging, as illustrated by the transitional system in Canada. It considered that changes in organisational thinking (e.g. in how laboratories and surveillance systems are arranged) will be one of the largest future challenges.

VII. Key sources/references

Cost questionnaire	Cost questionnaire completed by the National Microbiology Laboratory at the Public Health Agency of Canada
Preparatory phone interview	a) Background information and description of activities
Case study visit and follow up	b) Additional data and clarifications provided
Scientific literature	c) Remore J. et al (2018), 'Evaluation of whole-genome sequencing for outbreak detection of Verotoxigenic Escherichia coli O157:H7 from the Canadian perspective', BMC Genomics, 19(1):870. doi: 10.1186/s12864-018-5243-3.<br>d) Yachison, C.A., et al (2017), 'The Validation and Implications of Using Whole Genome Sequencing as a Replacement for Traditional Serotyping for a National Salmonella Reference Laboratory', Front Microbiology, 8:1044. doi: 10.3389/fmicb.2017.01044.
Other	e) PHAC website, https://www.canada.ca/en/public-health.html<br>f) National Microbiology Lab website https://www.canada.ca/en/public-health/programs/national-microbiology-laboratory.html<br>g) PulseNet Canada website https://www.canada.ca/en/public-health/programs/pulsenet-canada.html<br>h) IRIDA website https://www.irida.ca/<br>i) PHAC, Public Health Notice - Outbreaks of Salmonella infections linked to raw chicken, including frozen raw breaded chicken products https://www.canada.ca/en/public-health/services/public-health-notices/2018/outbreaks-salmonella-infections-linked-raw-chicken-including-frozen-raw-breaded-chicken-products.html

4.8. Public Health England (PHE)

**Foodborne pathogen surveillance – PHE, UK**			
**I. Institution**			
**Name of institution**	Public Health England (PHE)		
**Type of institution**	Executive agency of the Department of Health and Social Care		
**Description**[n)]	The Gastrointestinal Bacteria Reference Unit (GBRU) at Public Health England is the national reference laboratory for gastrointestinal bacterial pathogens for England, Wales and Northern Ireland from clinical, food and environmental samples. The GBRU also undertakes research into the genetic diversity of pathogens and the development of improved detection and characterisation techniques for food, water and environmentally borne diseases and offers expert advice, education and training on public health aspects of food microbiology and safety.<br>In 2012, Public Health England established a central genomics service at PHE Colindale to provide sequencing capabilities for microbiology services across PHE. Whilst initially focused on a few pathogens, including Salmonella, WGS is now being used by Public Health England for routine identification, characterisation and typing of Salmonella, Listeria, E. coli & Shigella, and Campylobacter isolates from England, Wales and Northern Ireland.[f)]		
**Location**	Greater London, UK		
**II. Surveillance activities covered by case study**			
**Activity**	Routine laboratory surveillance		
**Reference period**	04/2016 – 03/2017		
**Pathogen(s) covered**	Salmonella, Listeria, E. coli & Shigella, Campylobacter		
**Summary of routine surveillance activities using WGS**	WGS has been used for routine surveillance for all referred isolates of the listed pathogens since 2015 (Campylobacter since January 2016).		
**Type of sample**	Bacterial isolates from clinical, food and environmental samples		
**Region covered by laboratory surveillance**	England, Wales and Northern Ireland		
**Number of samples analysed in reference period**	*Pathogen*	*Samples analysed by conventional methods*	*Samples sequenced using WGS*
	Salmonella	The cost calculation is based on previous experiences with the listed conventional methods, assuming the same number of samples as with WGS	10174
	Listeria		1000
	E. coli & Shigella		4294
	Campylo-bacter		350
**Conventional methods used as reference for costing**	‣ Salmonella: Taqman PCR (73% of samples), Monophasic PCR for S. Typhimurium (10%), Serotyping (98%), Phage typing (99%), D-Tartrate (3%), Glucose gas test (3%), MLVA (48%), PFGE (3%), Antimicrobial resistance (AMR) testing (68%). Use of MLVA and PFGE for Salmonella was previously based on exceedance levels for certain serotypes/phage types.<br>‣ Listeria: PCR (x2; 100% each), fAFLP (100%).<br>‣ E. coli and Shigella: Real-time PCR (100%), Serotyping (100%), Phage typing (100%), Biochemistry (100%), MLVA (100%).		

	‣ Campylobacter: Real-time PCR (100%), Serotyping (12%), Phage typing (38%), MLST (52%). PHE indicated that serotyping and phage typing would have only been done in outbreaks.<br>‣ Sample preparation for serotyping was partly automated through the use of a robot for the preparation of antisera plates.
**Sample preparation WGS**	‣ Automated laboratory processes with minimal hands-on time (for example, DNA extraction is partially automated through the use of an automated DNA extraction machine).
**Sequencer used for WGS**	‣ Illumina HiSeq
**Batch size for WGS analysis**	‣ The data provided for the reference period assumes a run of 96 samples (or batches of 40 for sample processing)
**Reference dataset used for WGS**	‣ PHE uses its own in-house database for SNP analysis on a routine basis as well as other public databases on an ad hoc basis as required.

III. Detailed overview of costs of WGS and conventional methods

In the following, all costs are provided on a per-sample basis. Equipment costs are annualised and incorporate the annual maintenance costs as reported by the institution. They are adjusted for the percentage use of the equipment for the listed pathogens samples during the reference period (i.e. if a sequencer was also used for other purposes, this is taken into account). Consumables costs are adjusted for the failure rate (i.e. the percentage of consumables wasted, e.g. due to failed runs). Staff time is provided in terms of the minutes of hands-on staff time per sample, for both professionals and technicians. For the calculation of total costs, staff time is then monetised based on Eurostat data on country-specific labour costs for 2017 (by staff category), plus a 25% surcharge for overheads. For comparison purposes only, we have also provided staff costs monetised based on EU average labour costs. More detailed cost data is provided in Annex I.

a) Costs of using WGS[23]

Sample preparation and sequencing	*Cost type*	*Cost per sample*
	Equipment costs	€ 30.34
	Consumables	€ 53.92
	Other costs	€ 0
	Staff time professionals	*6.85 minutes*
	Staff time technicians	*17.15 minutes*
	Staff costs, monetised based on labour cost data for the UK (in brackets: based on labour cost data for the EU as a whole)	€ 11.67 (€ 12.15)
	**Total**	**€ 95.93**

Bioinformatics and other analyses	*Cost type*	*Cost per sample*
	Equipment costs	€ 4.89
	Other costs	€ 0
	Staff time professionals	*36 minutes*

[23] PHE provided cost data in pounds sterling. These have been converted to Euro using the European Central Bank’s yearly average reference exchange rate for the relevant year (i.e. the year of purchase for equipment, or 2017 otherwise).

	Staff time technicians	*0 minute*
	Staff costs, based on labour cost data for the UK (for EU)	€ 23.78 (€ 27.08)
	**Total**	**€ 28.67**

b) Costs of conventional methods

Note that detailed costing data were not available for every conventional test, as many of the conventional methods had been discontinued with the introduction of WGS. In consultation with PHE, it was decided to use similar tests for which data were available as a cost proxy. For example, as MLVA, MLST, and fAFLP are all enzyme reactions, the cost for MLVA was used as a proxy for the cost of MLST and fAFLP. Conventional tests were costed across all pathogens (e.g. the same per-sample cost calculation for Serotyping applies to Salmonella, Listeria, E. coli and Shigella, and Campylobacter).

Method A: PCR (Taqman)	*Cost type*	*Cost per sample*
	Equipment costs	€ 2.60
	Consumables	€ 2.12
	Other costs	€ 2.35
	Staff time professionals	*0 minutes*
	Staff time technicians	*5.63 minutes*
	Staff costs, based on labour cost data for the UK (for EU)	€ 2.35 (€ 2.30)
	**Total**	**€ 7.07**

Method B: PCR (Monophasic)	*Cost type*	*Cost per sample*
	Equipment costs	€ 2.60
	Consumables	€ 2.44
	Other costs	€ 0
	Staff time professionals	*0 minutes*
	Staff time technicians	*3.96 minutes*
	Staff costs, based on labour cost data for the UK (for EU)	€ 1.65 (€ 1.62)
	**Total**	**€ 6.69**

Method C: PCR (RT, other)	*Cost type*	*Cost per sample*
	Equipment costs	€ 5.12
	Consumables	€ 9.49
	Other costs	€ 0
	Staff time professionals	*2.50 minutes*
	Staff time technicians	*3.00 minutes*
	Staff costs, based on labour cost data for the UK (for EU)	€ 2.90 (€ 3.11)
	**Total**	**€ 17.51**

Method D: MLVA/MLST/fAFLP	*Cost type*	*Cost per sample*
	Equipment costs	€ 0
	Consumables	€ 3.87
	Other costs	€ 0
	Staff time professionals	*0 minutes*
	Staff time technicians	*7.71 minutes*
	Staff costs, based on labour cost data for the UK (for EU)	€ 3.21 (€ 3.15)
	**Total**	**€ 7.08**
Method E: Serotyping	*Cost type*	*Cost per sample*
	Equipment costs	€ 1.08
	Consumables	€ 13.36
	Other costs	€ 0
	Staff time professionals	*0 minutes*
	Staff time technicians	*27.25 minutes*
	Staff costs, based on labour cost data for the UK (for EU)	€ 11.35 (€ 11.13)
	**Total**	**€ 15.79**
Method F: Phage Typing	*Cost type*	*Cost per sample*
	Equipment costs	€ 0.08
	Consumables	€ 3.48
	Other costs	€ 0
	Staff time professionals	*2.25 minutes*
	Staff time technicians	*12.50 minutes*
	Staff costs, based on labour cost data for the UK (for EU)	€ 6.69 (€ 6.80)
	**Total**	**€ 10.26**
Method G: PFGE[24]	*Cost type*	*Cost per sample*
	Equipment costs	€ -
	Consumables	€ -
	Other costs	€ 97.82
	Staff time professionals	-
	Staff time technicians	-
	Staff costs, based on labour cost data for the UK (for EU)	€ - (€ -)

[24] Note that detailed cost data were not available for PFGE, so PHE's internal estimate of € 97.82 per sample was used as a unit cost.

	**Total**	**€ 97.82**
Method H: D-Tartrate	*Cost type*	*Cost per sample*
	Equipment costs	€ 0
	Consumables	€ 7.26
	Other costs	€ 0
	Staff time professionals	*0 minutes*
	Staff time technicians	*25.00 minutes*
	Staff costs, based on labour cost data for the UK (for EU)	€ 10.42 (€ 10.21)
	**Total**	**€ 17.67**
Method I: Glucose Gas	*Cost type*	*Cost per sample*
	Equipment costs	€ 0
	Consumables	€ 0.79
	Other costs	€ 0
	Staff time professionals	*0 minutes*
	Staff time technicians	*10.00 minutes*
	Staff costs, based on labour cost data for the UK (for EU)	€ 4.17 (€ 4.08)
	**Total**	**€ 4.96**
Method J: AMR	*Cost type*	*Cost per sample*
	Equipment costs	€ 0
	Consumables	€ 1.40
	Other costs	€ 0
	Staff time professionals	*0 minutes*
	Staff time technicians	*2.00 minutes*
	Staff costs, based on labour cost data for the UK (for EU)	€ 0.83 (€ 0.82)
	**Total**	**€ 2.23**
Method K: Biochemistry	*Cost type*	*Cost per sample*
	Equipment costs	€ 10.43
	Consumables	€ 25.97
	Other costs	€ 0
	Staff time professionals	*6.00 minutes*
	Staff time technicians	*36.00 minutes*
	Staff costs, based on labour cost data for the UK (for EU)	€ 18.96 (€ 19.21)
	**Total**	**€ 55.36**

IV. Costs of using WGS compared to the costs of conventional methods

The following comparison of costs per sample using WGS compared to the costs of conventional methods considers that the number of samples processed differed for the different conventional methods. The weighted cost of the conventional methods provided here is therefore a weighted figure which accounts for the use rate of the various methods across the different pathogens. See Annex I for more details.

Comparison of equipment costs

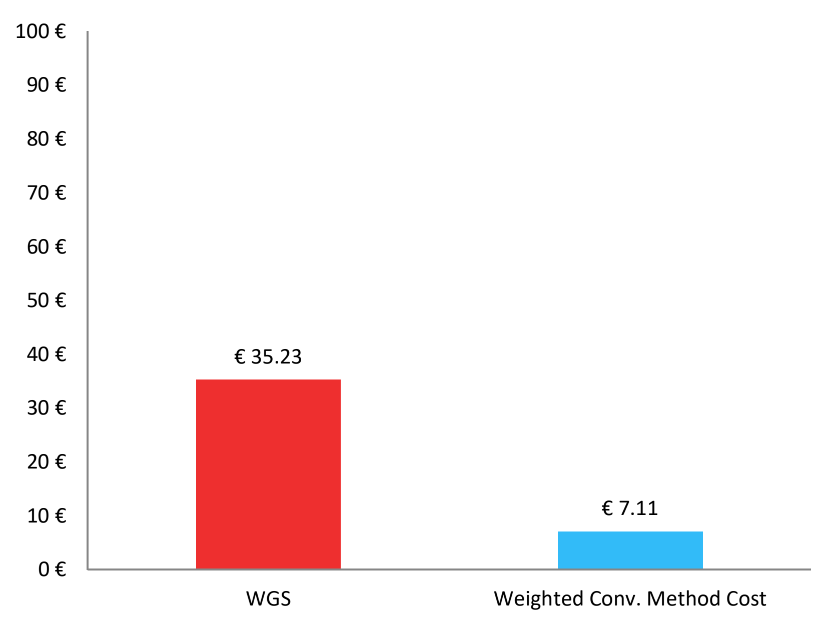

Equipment costs *per sample* at PHE are higher for WGS than for the weighted conventional methods (€ 35.23 vs € 7.11), although the large volume of samples processed (15 791 during the reference period) keeps equipment costs for both WGS and conventional methods low on a per-sample basis. The large sample size and the relatively lower cost of the equipment used for the conventional methods brings the per-sample weighted cost for the conventional methods down to just € 7.11.

Comparison of costs of consumables

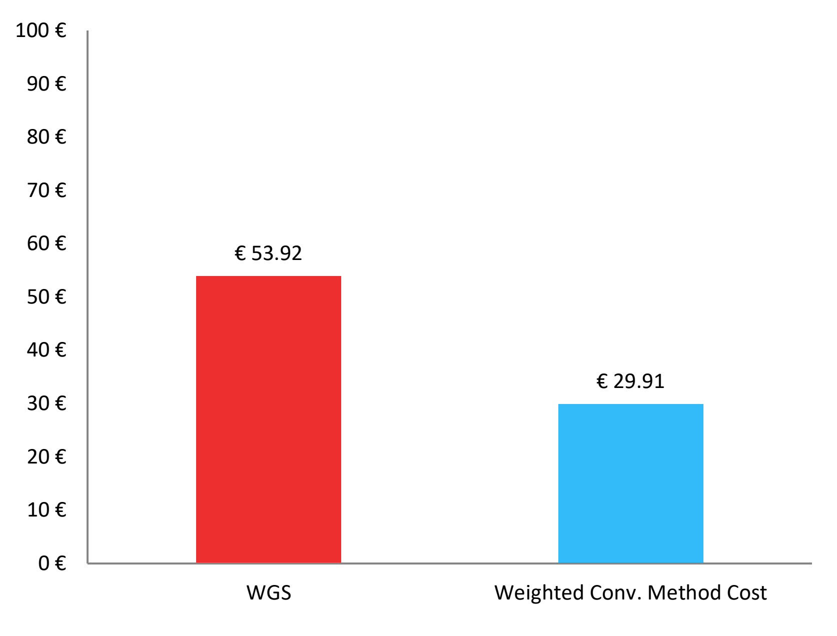

Consumables costs for WGS (€ 53.92) are higher than for conventional methods (€ 29.91). The higher costs for WGS result from the higher per-

sample costs of the various kits used for library preparation, particularly the Nextera DNA Library Prep Kit for 96 samples.

Comparison of staff time used (in minutes)

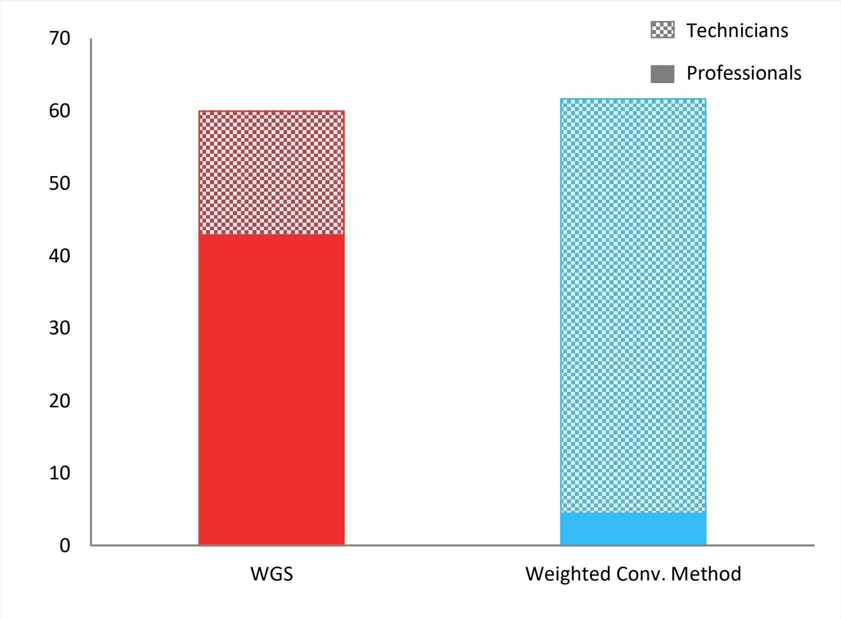

The amount of staff time needed for WGS analysis (a total of 60 minutes per sample) is slightly lower than the amount of staff time needed to carry out the various conventional methods (62 minutes). Note however that as with the other cost categories, the staff time required for conventional methods was weighted to take into account the fact that multiple tests were often performed on the same samples. The staff time required for individual conventional tests ranged from a low of 2 minutes per sample (for AMR testing) to a high of 42 minutes per sample (for the biochemistry tests).

Compared to conventional methods, analysis with WGS requires a significantly larger proportion of professional staff time. As a result, once staff time has been monetised, WGS has higher staff costs (€ 35.44) than the weighted conventional methods (€ 26.77).

Comparison of overall costs

Cost type	*Cost per sample (WGS)*	*Cost per sample (conventional methods)*
Equipment costs	€ 35.23	€ 7.11
Consumables	€ 53.92	€ 29.91
Other costs	€ 0	€ 1.67
Staff time professionals	*42.85 minutes*	*4.43 minutes*
Staff time technicians	*17.15 minutes*	*57.23 minutes*
Staff costs, based on labour cost data for the UK (for EU)	€ 35.44 (€ 39.23)	€ 26.77 (€ 26.70)
**Total**	**€ 124.59**	**€ 65.46**

Differential costs

The cost difference between WGS and conventional methods is € 59.13 per sample. A sample analysed with WGS costs approximately twice the amount of analysis with conventional methods. As indicated in the figures above, the largest differences are in equipment and consumables costs.

**V. Effects of using WGS results**	
a) Turnaround time. Turnaround time is defined as the usual number of days of work from receipt and opening of an incoming sample until the reporting of results. Turnaround time does not include weekends and holidays, except in case that work has been conducted on these days, e.g. for a sequencing run or other analyses.	
Turnaround time	▸ The turnaround time for the analysis of a sample using WGS for pathogen identification is 10 days of work. This figure includes weekends, as machines can be set to run over the weekend.<br>▸ The turnaround time using the specified conventional methods for pathogen identification is dependent on the pathogen. For example, the turnaround time would be 10-15 days of work for Salmonella (14-21 days including weekends, as machines can be set to run over the weekend), or 3 days of work for L. monocytogenes (5 days including weekends). However, these estimates do not include typing, but just confirmation of identification and serotyping.<br>▸ PHE considered that for most pathogens there has been an improvement in turnaround times with WGS. However, this depends on the type of analysis needed: for example, some of PHE's clients only need confirmation of identity, which takes longer with WGS than using conventional methods (i.e. PCR identification). As a result, in cases where identification is required urgently, PHE still does PCR identification tests.
b) Positive effects of using WGS for pathogen identification and surveillance during the reference period	
Sampling and sampling strategies	▸ PHE reported no effects at all on sampling and sampling strategies.
Analytical results and processes	▸ PHE indicated that WGS had very significant positive effects on analytical results and processes. It considered that WGS had significantly improved the accuracy, sensitivity, specificity, and level of detail in the results produced, citing papers in which the higher resolution data from WGS was used to produce results above and beyond what would be possible with conventional methods alone.[f)-k)] For example, PHE indicated that WGS can show how strains diversify over time, allowing strains to be identified as being phylogenetically linked, while under past methods these would have been seen to be unrelated strains.<br>▸ PHE indicated that WGS had led to considerable streamlining in their laboratory. It reported that WGS had simplified laboratory work flows, noting that WGS was able to replace the numerous tests that had previously been performed on each pathogen with a single, unified workflow. PHE also indicated that WGS had led to a reduction in (analytical) time, staff time, and consumables. PHE reported having reduced their lab staff considerably since introducing WGS.<br>▸ Another benefit noted by PHE was the ability to better monitor its own laboratory processes. PHE was able to introduce processes to report on the use of WGS (e.g. related to the number of samples processed) and indicated that it used this data to track trends in WGS usage, predict future costs, and try to reduce costs in the future.
Outbreak identification and response	▸ PHE indicated that the impact of using WGS in pathogen surveillance has been ‘transformational’. It stated that WGS has dramatically changed outbreak detection, namely that more outbreaks were being detected than previously;[h)] that large multinational outbreaks are being detected that would have not been detected and confirmed with certainty before;[d)] and that ‘slow burn’ outbreaks with few cases over several years can also now be detected. For example, WGS was able to identify an outbreak of

	Salmonella enteritidis in reptile feeder mice that had previously been continuing undetected over a period of four years with at least 162 cases identified between 2012 and 2015.[c)]<br>▸ PHE also indicated that one of the benefits of WGS was the ability to monitor the effectiveness of public health interventions. As an example, it cited the case of a large EU-wide Salmonella outbreak in eggs, where action was taken to address the problem but WGS was able to identify the re-emergence of human cases, indicating an ongoing issue. With previous typing methods it would not have been possible to show it was the same strain with the level of certainty provided by WGS.<br>▸ PHE reported that WGS can be used for more precise case definitions in outbreak investigations. It noted that WGS provided a tool to rule cases as being in or out of the outbreak far more accurately, making subsequent epidemiological investigations more powerful by not including cases that were not actually part of the outbreak. For example, WGS was used by PHE to discriminate between three separate outbreaks of Shigella in the English Orthodox Jewish community which were circulating at the same time.[i),m)]<br>▸ PHE noted that WGS also allowed them to identify whether an outbreak isolate was likely to have come from outside the UK through clustering with travel-related isolates or comparisons with sequence data in external databases. It considered that WGS enabled the tracking and dissemination of emerging strains at a global scale.<br>▸ In sum, PHE indicated that WGS had highly significant effects on the earlier detection of an initial outbreak, improved detection that outbreaks are related, improved information on outbreak epidemiology, and improved information for imposing additional control or biosecurity measures. It also considered that WGS had contributed to a reduction in the duration of outbreaks, and had likely contributed to a reduction of the disease burden in humans (although it stated that it had not observed this directly, and that this effect might take longer to see).
Research and methods applied	▸ PHE reported very significant positive effects of using WGS regarding better understanding of disease transmission. For example, PHE described a case where an E. coli O157 isolate causing an outbreak via salad leaves was matched to isolates from UK sheep, leading it to determine that the salad leaves most likely became contaminated as a result of being grown or irrigated with river water contaminated by run-off from nearby fields where sheep had been grazing.<br>▸ PHE also noted other benefits for research, in particular the fact that large amounts of WGS data (sequence data) are now made publicly available and can be used freely for analysis. It also noted that WGS data made it easier to collaborate internationally, since it is now possible to send sequence data instead of isolates.<br>▸ Moderate effects of WGS were observed by PHE with respect to improvements in epidemiological methods. PHE indicated that the use of WGS in case definitions improves the power of analytical epidemiological studies, citing the previously-mentioned study concerning a long-undetected Salmonella outbreak linked to reptile feeder mice.[c)]<br>▸ Moderate effects of WGS were also observed regarding the development of better diagnostic tests. For example, PHE cited a paper co-authored by its staff which demonstrates the use of WGS as a resource for the development and evaluation of molecular diagnostic assays for Campylobacter.[l)] PHE also noted that it had recently developed and implemented a PCR assay to distinguish between typhi/paratyphi and non-typhoidal strains of Salmonella, and that it had been able to design the primers and probes and carry out extensive validation of these on a panel of over 1000 WGS results from different Salmonella samples.
Effects on wider society	▸ PHE considered that it was not able to fully assess the effects of using WGS

	on the wider society. Nevertheless, it did indicate that WGS had led to a reduction of costs of outbreaks for the wider society, citing the general principle that identifying an outbreak and putting in preventative measures should lead to the prevention of further cases going forward.<br>- PHE also considered that WGS had likely reduced the negative effects of outbreaks on consumer trust in food.
c) Negative effects of using WGS	
Negative effects of using WGS	PHE indicated that since switching to WGS, it is detecting far more outbreaks than previously (particularly with respect to Salmonella), and that this has resource implications for their epidemiological investigations.[g),h)] PHE indicated that it currently doesn't have the resources to investigate all the linked cases that they see with WGS. However, it noted that if more outbreaks are resolved, then this would lead to a reduction in the disease burden overall.
**VI. Outlook**	
Balance of costs and benefits achieved	- PHE considered that their costs had increased due to an increase in the number of outbreaks detected through WGS. However, it expected that if preventative measures are successfully implemented on the basis of better outbreak detection, improved understanding, investigation and implementation of effective control measures, the overall costs should come down from both a societal and an institutional perspective.
Potential for cost reductions	- PHE expected costs to come down in the long term as laboratories reorganise their operations around WGS (e.g. by replacing conventional typing methods for other gastrointestinal pathogens, through streamlining processes and needing fewer staff). It considered that there would likely be future improvements in bioinformatics, i.e. in algorithm development, which could further streamline the analysis and reduce costs.<br>- PHE also expected to see a long term reduction in the costs of outbreak detection and response through the prevention of future cases.
Future opportunities and challenges	- PHE considered that the full potential of WGS technology has probably not yet been fully realised, and that WGS will lead to better information on transmission of gastrointestinal pathogens and improve epidemiological investigations. It reported that some effects of WGS (e.g. on staff costs and laboratory organisation, but also on wider effects such improved epidemiological investigations and the reduction of the overall disease burden) would take longer to see.<br>- PHE considered that the MinION had a lot of potential for outbreak response in the future, and could also provide a way for laboratories to diversity their technology against price increases through supplier monopolies (e.g. from supplies who are the sole producers of necessary sequencing kits). It also considered that the MinION could be a valuable tool in developing countries, and thought there was potential for these countries to 'leapfrog' previous technology and jump right into sequencing.<br>- PHE noted that back-compatibility could be a concern going forward, as the new information provided by WGS is very different from what was collected before (e.g. through phage typing). This could cause difficulties in inter-agency communication with agencies that do not yet use WGS.<br>- Training and communications were noted as a present and future challenge, since PHE noted that WGS has a steep learning curve and re-training can require significant resources.<br>- Another future challenge noted by PHE related to the availability of bioinformatics skills, since the bioinformatics analysis requires a very

	specific set of skills in computer science, statistics, biology, and epidemiology, and people with this expertise can be difficult to recruit.
**VII. Key sources/references**	
Cost questionnaire	Cost questionnaire completed by PHE
Preparatory phone interview	a) Background information and description of activities
Case study visit and follow up	b) Additional data and clarifications provided
Scientific literature	c) Kanagarajah, S., Waldram, A., Dolan, G., Jenkins, C., Ashton, P. M., Martin, A. I. C., ... et al. (2018). Whole genome sequencing reveals an outbreak of Salmonella Enteritidis associated with reptile feeder mice in the United Kingdom, 2012-2015. Food microbiology, 71, 32-38.<br>d) Inns, T., Ashton, P. M., Herrera-Leon, S., Lighthill, J., Foulkes, S., Jombart, T., et al. (2017). Prospective use of whole genome sequencing (WGS) detected a multi-country outbreak of Salmonella Enteritidis. Epidemiology & Infection, 145(2), 289-298.<br>e) Ashton, P. M., Nair, S., Peters, T. M., Bale, J. A., Powell, D. G., Painset, A.,et al. (2016). Identification of Salmonella for public health surveillance using whole genome sequencing. PeerJ, 4, e1752.<br>f) Ashton, P., Nair, S., Peters, T., Tewolde, R., Day, M., Doumith, M., et al. (2015). Revolutionising public health reference microbiology using whole genome sequencing: Salmonella as an exemplar. bioRxiv, 033225.<br>g) Waldram, A., Dolan, G., Ashton, P. M., Jenkins, C., & Dallman, T. J. (2018). Epidemiological analysis of Salmonella clusters identified by whole genome sequencing, England and Wales 2014. Food microbiology, 71, 39-45.<br>h) Mook P, Gardiner D, Verlander NQ, McCormick J, Usdin M, Crook P, Jenkins C, Dallman TJ. Operational burden of implementing Salmonella Enteritidis and Typhimurium cluster detection using whole genome sequencing surveillance data in England: a retrospective assessment. Epidemiol Infect. 2018 Jul 2:1-9.<br>i) Vanessa Rew, Piers Mook, Suzan Trienkens, Kate S Baker, Timothy J Dallman, Claire Jenkins, Paul D Crook and Nicholas R Thomson. Whole-genome sequencing revealed concurrent outbreaks of shigellosis in the English Orthodox Jewish Community caused by multiple importations of Shigella sonnei from Israel. Microbial Genomics, 2018:4.<br>j) Butcher H, Elson R, Chattaway MA, Featherstone CA, Willis C, Jorgensen F, Dallman TJ, Jenkins C, McLauchlin J, Beck CR, Harrison S. Whole genome sequencing improved case ascertainment in an outbreak of Shiga toxin-producing Escherichia coli O157 associated with raw drinking milk. Epidemiol Infect. 2016 Oct;144(13):2812-23. Epub 2016 Mar 10<br>k) Timothy J. Dallman, Marie A. Chattaway, Piers Mook, Gauri Godbole, Paul D. Crook, Claire Jenkins. Use of whole-genome sequencing for the public health surveillance of Shigella sonnei in England and Wales, 2015. 2016, Journal of Medical Microbiology 65: 882-884<br>l) Jansen van Rensburg MJ, Swift C, Cody AJ, Jenkins C, Maiden MC. Exploiting Bacterial Whole-Genome Sequencing Data for Evaluation of Diagnostic Assays: Campylobacter Species Identification as a Case Study. J Clin Microbiol. 2016 Dec;54(12):2882-2890. Epub 2016 Oct 12<br>m) J. Mcdonnell, T. Dallman, S. Atkin, D. A. Turbitt, T. R. Connor, K. A. Grant, N. R. Thomson And C. Jenkins. Retrospective analysis of whole genome sequencing compared to prospective typing data in further informing the epidemiological investigation of an outbreak of Shigella sonnei in the UK Epidemiol. Infect. (2013), 141, 2568–2575. Cambridge University Press 2013
Other	n) Website, Gastrointestinal bacteria reference unit (GBRU) https://www.gov.uk/guidance/gbru-reference-and-diagnostic-services<br>o) Pathogen Genomics Into Practice, PHG Foundation, 2015.

Appendix. Cost data collected from case study institutions

ANNEX : Data collected for cost calculation - APHA

I. WGS

Equipment

In the following, the equipment used for sample preparation, sequencing, bioinformatics and other analyses considered for the cost calculation is listed. For each piece of equipment, the table provides the total unit price at the time of purchase (including VAT), annual maintenance costs, and predicted lifespan. Only equipment was considered that costed EUR 400 or more that qualify as capital expenditure relevant for WGS, such as sequencing machines and durable lab equipment as well as specific software purchasing or licensing fees. Not included were basic laboratory equipment (e.g. refrigerators, centrifuges or pipettes), standard office computers and standard office software.

This approach was similarly applied for all methods listed below.

	Total purchase price (Euro)	*Annual maintenance costs (Euro)*	*Predicted lifespan (years)*
Illumina MiSeq	€ 104 826	€ 12 000	10
Computer	€ 2 355	€ 0	5

Consumables

In the following, the consumables used for sample preparation and sequencing considered for the cost calculation are listed. Consumables include items that are used up in laboratory processes, such as chemicals, petri dishes, etc. For each item, the table provides the cost per sample, the step of analysis it is used for and the failure rate. The failure rate refers to the percentage of consumables that are wasted, e.g. due to failed runs, and is taken into account in the cost calculation.

This approach was similarly applied for all methods listed below.

	Cost per sample (Euro)	*Step of analysis*	*% failure*
Qiagen viral RNA extraction kit	€ 4.59	Sample processing	1
Roche cDNA synthesis kit	€ 69.58	Library preparation	1
Nextera XT kit	€ 748.57	Sequencing	1

Staff time per sample in minutes

The following provides the estimated staff time per sample spent on each step, separately for professionals and for technicians. The amount of 'hands-on staff time' is indicated, i.e. the amount of staff time actually used to perform an activity, and not the duration of the activity, including for maintenance of equipment and staff time used for failed runs. Where several samples are treated at the same time, total staff time is divided to obtain the per-sample staff time. For example, if sample processing for 40 samples takes 2 hours and 40 minutes for a laboratory technician, this figure is converted to minutes (160 minutes), and divided by 40, resulting in a technician staff time of 4 minutes per sample.

This approach was similarly applied for all methods listed below.

Staff category / *Step*	*Professionals* (staff time in minutes)*	*Technicians** (staff time in minutes)*
Sample processing	0	60
Library preparation	0	60
Sequencing	0	90
Bioinformatics & other analyses	60	0

Reference dataset	0	0

The definition of these catgories is based on the International Standard Classification of Occupations of the International Labour Office (ILO).

**For "Professionals", occupations typically involve the performance of tasks that require complex problem-solving, decision-making and creativity based on an extensive body of theoretical and factual knowledge in a specialised field. The knowledge and skills required are typically obtained as the result of study at a higher educational institution for a period of 3-6 years following completion of secondary education leading to the award of a first degree or higher qualification. This category includes PhD candidates and Post-docs.*

***For "Technicians", occupations typically involve the performance of complex technical and practical tasks that require an extensive body of factual, technical and procedural knowledge in a specialised field. The knowledge and skills required are usually obtained as the result of study at a higher educational institution for a period of 1-3 years following completion of secondary education. This category includes laboratory assistants.*

II. Conventional method A: Sanger Sequencing

Equipment

	Total purchase price (Euro)	*Annual maintenance costs (Euro)*	*Predicted lifespan (years)*
ABI Capillary sequencer 37/30	€ 198 667	€ 8 000	10
G storm thermocycler	€ 2 355	€ 388	5
LazerGene software licence	€ 16 474	0	1

Consumables

	Cost per sample (Euro)	*% failure*
Viral RNA extraction kit	€ 4.59	1
PCR kit	€ 4.75	1
Gel extraction kit	€ 1.68	0
Labelling kit	€ 10.28	5

Staff time per sample in minutes

	Professionals	*Technicians*
Staff time in minutes	60	360

III. Key variables

Labour costs

The following table provides the hourly labour cost data (in Euro) used for monetisation of staff time. Figures below refer to Eurostat data on labour costs for 2017 (by staff category), plus a 25% surcharge for overheads.

	Professionals	*Technicians*
UK	€ 39.63	€ 25.00
EU	€ 45.13	€ 24.50

Source: Eurostat, Labour cost levels by NACE Rev. 2 activity [lc_lci_lev]. Construct: Labour cost for LCI

(compensation of employees plus taxes minus subsidies). NACE categories: Professional, scientific and technical activities; Administrative and support service activities. Extracted in June 2018.	
Other	
…	

ANNEX : Data collected for cost calculation - FLI

I. WGS

Equipment

In the following, the equipment used for sample preparation, sequencing, bioinformatics and other analyses considered for the cost calculation is listed. For each piece of equipment, the table provides the total unit price at the time of purchase (including VAT), annual maintenance costs, and predicted lifespan. Only equipment was considered that costed EUR 400 or more that qualify as capital expenditure relevant for WGS, such as sequencing machines and durable lab equipment as well as specific software purchasing or licensing fees. Not included were basic laboratory equipment (e.g. refrigerators, centrifuges or pipettes), standard office computers and standard office software. Note that the predicted lifespan of equipment is based on standard values and applied uniformly across case studies. Lifespans used for accounting purposes by each case institution may differ.

This approach was similarly applied for all methods listed below.

	Total purchase price (Euro)	*Annual maintenance costs (Euro)*	*Predicted lifespan (years)*
Covaris sonicator	€ 27 300	€ 0	10
Agilent bioanalyzer	€ 22 000	€ 0	10
Ion Torrent PGM bundle	€ 93 000	€ 11 500	10
Server for assembly computation	€ 34 700	€ 0	5

Consumables

In the following, the consumables used for sample preparation and sequencing considered for the cost calculation are listed. Consumables include items that are used up in laboratory processes, such as chemicals, petri dishes, etc. For each item, the table provides the cost per sample, the step of analysis it is used for and the failure rate. The failure rate refers to the percentage of consumables that are wasted, e.g. due to failed runs, and is taken into account in the cost calculation.

This approach was similarly applied for all methods listed below.

	Cost per sample (Euro)	*Step of analysis*	*% failure*
96-Well PCR-plates qPCR	€ 0.86	Sample processing	10
96-Well PCR-plates PCR	€ 0.69		
Reaction tubes 1.5 ml	€ 0.40		
Reaction tubes 2 ml	€ 0.54		
Pipette tips 1000 µl	€ 1.12		
Pipette tips 200 µl	€ 1.05		
Pipette tips 100 µl	€ 1.05		
Pipette tips 10 µl	€ 1.05		
Pipette tips 2 µl	€ 1.05		
RNA-Purification	€ 5.59		
Gelextraction/DNA-Purification	€ 2.04		
DNA/RNA-Extraction	€ 3.71		
RT-PCR	€ 5.15		
PCR	€ 1.33		
Lab gloves	€ 3.76		

Covaris-Vials	€ 6.91		
Agilent Bioanalyzer RNA Pico Kit	€ 5.00		
GeneRead Library Prep Kit	€ 29.35	Library preparation	10
Adapter	€ 12.01		
Agilent Bioanalyzer DNA HS Kit	€ 5.86		
KAPA Library Quant IonTorrent	€ 23.64		
Onetouch Reagents	€ 21.31	Sequencing	10
Enrichment Beads	€ 0.86		
Chips (316v2)	€ 50.52		
Sequencing Reagents	€ 45.16		
Nitrogen	€ 0.48		
W2-Bottles	€ 1.21		

Staff time per sample in minutes

The following provides the estimated staff time per sample spent on each step, separately for professionals and for technicians. The amount of 'hands-on staff time' is indicated, i.e. the amount of staff time actually used to perform an activity, including maintenance of equipment and staff time used for failed runs, but excluding unsupervised processes (e.g. time that the sequencer is running unsupervised). Where several samples are treated at the same time, total staff time is divided to obtain the per-sample staff time. For example, if sample processing for 40 samples takes 2 hours and 40 minutes for a laboratory technician, this figure is converted to minutes (160 minutes), and divided by 40, resulting in a technician staff time of 4 minutes per sample.

This approach was similarly applied for all methods listed below.

Staff category<br>*Step*	*Professionals** (staff time in minutes)*	*Technicians** (staff time in minutes)*
Sample processing	8	40
Library preparation	3	60
Sequencing	7	35
Bioinformatics & other analyses	20	0
Reference dataset	10	0

The definition of these categories is based on the International Standard Classification of Occupations of the International Labour Office (ILO).

**For "Professionals", occupations typically involve the performance of tasks that require complex problem-solving, decision-making and creativity based on an extensive body of theoretical and factual knowledge in a specialised field. The knowledge and skills required are typically obtained as the result of study at a higher educational institution for a period of 3-6 years following completion of secondary education leading to the award of a first degree or higher qualification. This category includes PhD candidates and Post-docs.*

***For "Technicians", occupations typically involve the performance of complex technical and practical tasks that require an extensive body of factual, technical and procedural knowledge in a specialised field. The knowledge and skills required are usually obtained as the result of study at a higher educational institution for a period of 1-3 years following completion of secondary education. This category includes laboratory assistants.*

**II. Conventional method A: Sanger Sequencing**			
Equipment			
	Total purchase price (Euro)	*Annual maintenance costs (Euro)*	*Predicted lifespan (years)*
ABI Sequencer	€ 120 000	€ 8 000	10

Consumables		
	Cost per sample (Euro)	*% failure**
96-Well PCR-plates qPCR	€ 0.86	10
96-Well PCR-plates PCR	€ 0.69	
Reaction tubes 1.5 ml	€ 0.40	
Reaction tubes 2 ml	€ 0.54	
Pipette tips 1000 µl	€ 1.12	
Pipette tips 200 µl	€ 1.05	
Pipette tips 100 µl	€ 1.05	
Pipette tips 10 µl	€ 1.05	
Pipette tips 2 µl	€ 1.05	
RNA-Purification	€ 10.00	
Gelextraction/DNA-Purification	€ 24.25	
RT-PCR	€ 34.87	
Lab gloves	€ 3.76	
EtOH	€ 0.05	0
2-Mercaptoethanol	€ 0.02	
Agarose	€ 1.95	
TBE-Buffer (0.5X)	€ 0.52	
Ethidiumbromid-Lsg.	€ 0.26	
52/4000 Seq.-Kit	€ 120.00	
Nucleoseq Columns	€ 72.28	
Formamide	€ 0.50	
Capillary array	€ 39.80	
Sequencing buffer	€ 0.13	
Polymer POP7	€ 36.60	

Staff time per sample in minutes		
	Professionals	*Technicians*
Staff time in minutes	260	240

III. Key variables

Labour costs		
The following table provides the hourly labour cost data (in Euro) used for monetisation of staff time. Figures below refer to Eurostat data on labour costs for 2017 (by staff category), plus a 25% surcharge for overheads.		
	Professionals	*Technicians*
Germany	€ 53.3	€ 26.8
EU	€ 45.1	€ 24.5
Source: Eurostat, Labour cost levels by NACE Rev. 2 activity [lc_lci_lev]. Construct: Labour cost for LCI (compensation of employees plus taxes minus subsidies). NACE categories: Professional, scientific and technical activities; Administrative and support service activities. Extracted in June 2018.		

ANNEX : Data collected for cost calculation - EMC

I. WGS

Equipment

In the following, the equipment used for sample preparation, sequencing, bioinformatics and other analyses considered for the cost calculation is listed. For each piece of equipment, the table provides the total unit price at the time of purchase (including VAT), annual maintenance costs, and predicted lifespan. Only equipment was considered that costed EUR 400 or more that qualify as capital expenditure relevant for WGS, such as sequencing machines and durable lab equipment as well as specific software purchasing or licensing fees. Not included were basic laboratory equipment (e.g. refrigerators, centrifuges or pipettes), standard office computers and standard office software. Note that the predicted lifespan of equipment is based on standard values and applied uniformly across case studies. Lifespans used for accounting purposes by each case institution may differ.

This approach was similarly applied for all methods listed below.

	Total purchase price (Euro)	*Annual maintenance costs (Euro)*	*Predicted lifespan (years)*
Gel electrophoreses system	€ 4 000	€ 0	10
PCR machine	€ 5 000	€ 0	10
Qubit	€ 3 000	€ 0	10
Magnate 96 wells	€ 800	€ 0	10
GridION	€ 45 000	€ 4 500	10
Computer (server)	€ 15 060	€ 0	5
Computer (back-up)	€ 0	€ 700	1
Computer (CLC)	€ 1 000	€ 0	5
CLC Software	€ 500	€ 0	1

Consumables

In the following, the consumables used for sample preparation and sequencing considered for the cost calculation are listed. Consumables include items that are used up in laboratory processes, such as chemicals, petri dishes, etc. For each item, the table provides the cost per sample, the step of analysis it is used for and the failure rate. The failure rate refers to the percentage of consumables that are wasted, e.g. due to failed runs, and is taken into account in the cost calculation.

This approach was similarly applied for all methods listed below.

	Cost per sample (Euro)	*Step of analysis*	*% failure*
RNA isolation kit	€ 6.00	Sample processing	20
RT-PCR kit	€ 5.00		
Consumables	€ 3.00		
Ligase	€ 0.50	Library Preparation	0
Sequencing kit	€ 2.50		
Consumables	€ 2.50		
Flowcell	€ 11.00	Sequencing	2

Staff time per sample in minutes

The following provides the estimated staff time per sample spent on each step, separately for professionals and for technicians. The amount of 'hands-on staff time' is indicated, i.e. the amount of staff

time actually used to perform an activity, including maintenance of equipment and staff time used for failed runs, but excluding unsupervised processes (e.g. time that the sequencer is running unsupervised). Where several samples are treated at the same time, total staff time is divided to obtain the per-sample staff time. For example, if sample processing for 40 samples takes 2 hours and 40 minutes for a laboratory technician, this figure is converted to minutes (160 minutes), and divided by 40, resulting in a technician staff time of 4 minutes per sample.

This approach was similarly applied for all methods listed below.

Staff category<br>*Step*	*Professionals**<br>*(staff time in minutes)*	*Technicians***<br>*(staff time in minutes)*
Sample processing	0	48
Library preparation	0	13
Sequencing	6	6
Bioinformatics & other analyses	12	24
Reference dataset	0	0

The definition of these catgories is based on the International Standard Classification of Occupations of the International Labour Office (ILO).

**For "Professionals", occupations typically involve the performance of tasks that require complex problem-solving, decision-making and creativity based on an extensive body of theoretical and factual knowledge in a specialised field. The knowledge and skills required are typically obtained as the result of study at a higher educational institution for a period of 3-6 years following completion of secondary education leading to the award of a first degree or higher qualification. This category includes PhD candidates and Post-docs.*

***For "Technicians", occupations typically involve the performance of complex technical and practical tasks that require an extensive body of factual, technical and procedural knowledge in a specialised field. The knowledge and skills required are usually obtained as the result of study at a higher educational institution for a period of 1-3 years following completion of secondary education. This category includes laboratory assistants.*

II. Conventional method A: Real Time PCR

Equipment

	Total purchase price (Euro)	*Annual maintenance costs (Euro)*	*Predicted lifespan (years)*
Lightcycler	€ 40 200	€ 3 931	10
Magnapure 96	€ 125 619	€ 9 309	10

Consumables

	Cost per sample (Euro)	*% failure**
RNA isolation kit	€ 6.00	0
Real Time PCR kit (5x per sample)	€ 25.00	

Staff time per sample in minutes

	Professionals	*Technicians*
Staff time in minutes	0	84

III. Conventional method B: Sanger Sequencing

Equipment			
	Total purchase price (Euro)	*Annual maintenance costs (Euro)*	*Predicted lifespan (years)*
3130XL sequencer	€ 44 118	€ 13 759	10
Computer + DNAstar	€ 500	€ 0	5

Consumables		
	Cost per sample (Euro)	*% failure**
RT-PCR Kit (2x per sample HA NA)	€ 20.00	0
Big Dye Terminator	€ 0.75	
Consumables	€ 3.00	

Staff time per sample in minutes		
	Professionals	*Technicians*
Staff time in minutes	0	60

III. Conventional method C: Virus isolation

Equipment			
	Total purchase price (Euro)	*Annual maintenance costs (Euro)*	*Predicted lifespan (years)*
CO2 incubators	€ 14 528	€ 0	10

Consumables		
	Cost per sample (Euro)	*% failure**
Culture media and plasticware	€ 10.00	0

Staff time per sample in minutes		
	Professionals	*Technicians*
Staff time in minutes	0	30

IV. Conventional method D: Hemagglutination inhibition

Equipment			
	Total purchase price (Euro)	*Annual maintenance costs (Euro)*	*Predicted lifespan (years)*
Tecan EVO	€ 59 000	€ 6 000	15

Consumables		
	Cost per sample (Euro)	*% failure**
Plasticware, red blood cells, ferret sera	€ 3.00	0

Staff time per sample in minutes			
	Professionals	*Technicians*	
Staff time in minutes	5	18	

**V. Conventional method E: Virus neutralisation**			
Equipment			
	Total purchase price (Euro)	*Annual maintenance costs (Euro)*	*Predicted lifespan (years)*
CTL-immunospot	€ 100 000	€ 0	10
Consumables			
	Cost per sample (Euro)	*% failure**	
Plasticware, red blood cells, ferret sera	€ 13.00	0	
Staff time per sample in minutes			
	Professionals	*Technicians*	
Staff time in minutes	5	102	

**VI. Conventional method F: NA star**			
Equipment			
	Total purchase price (Euro)	*Annual maintenance costs (Euro)*	*Predicted lifespan (years)*
Tecan Infinite	€ 25 000	€ 2 500	15
Consumables			
	Cost per sample (Euro)	*% failure**	
Chemicals	€ 2.00	0	
Staff time per sample in minutes			
	Professionals	*Technicians*	
Staff time in minutes	0	42	

**XIII. Key variables**		
Labour costs		
The following table provides the hourly labour cost data (in Euro) used for monetisation of staff time. Figures below refer to Eurostat data on labour costs for 2017 (by staff category), plus a 25% surcharge for overheads.		
	Professionals	*Technicians*
Netherlands	€ 53.20	€ 28.20

EU	€ 45.10	€ 24.50
Source: Eurostat, Labour cost levels by NACE Rev. 2 activity [lc_lci_lev]. Construct: Labour cost for LCI (compensation of employees plus taxes minus subsidies). NACE categories: Professional, scientific and technical activities; Administrative and support service activities. Extracted in June 2018.		
Other		
…		

ANNEX: Data collected for cost calculation - IZLER

I. WGS

Equipment

In the following, the equipment used for sample preparation, sequencing, bioinformatics and other analyses considered for the cost calculation is listed. For each piece of equipment, the table provides the total unit price at the time of purchase (including VAT), annual maintenance costs, and predicted lifespan. Only equipment was considered that costed EUR 400 or more that qualify as capital expenditure relevant for WGS, such as sequencing machines and durable lab equipment as well as specific software purchasing or licensing fees. Not included were basic laboratory equipment (e.g. refrigerators, centrifuges or pipettes), standard office computers and standard office software. Note that the predicted lifespan of equipment is based on standard values and applied uniformly across case studies. Lifespans used for accounting purposes by each case institution may differ.

This approach was similarly applied for all methods listed below.

	Total purchase price (Euro)	*Annual maintenance costs (Euro)*	*Predicted lifespan (years)*
Biorad-T100 thermal cycler	€ 4 000	€ 0	10
Biorad-CFX96 RT-System	€ 24 400	€ 0	10
Microplate-Genie-Shaker	€ 700	€ 0	10
MiSeq (Illumina, USA)	€ 100 000	€ 12 000	10
Workstations (3 pieces)	€ 5 000	€ 0	5
Storage unit	€ 18 500	€ 0	5
Bionumerics License	€ 10 720	€ 0	10

Consumables

In the following, the consumables used for sample preparation and sequencing considered for the cost calculation are listed. Consumables include items that are used up in laboratory processes, such as chemicals, petri dishes, etc. For each item, the table provides the cost per sample, the step of analysis it is used for and the failure rate. The failure rate refers to the percentage of consumables that are wasted, e.g. due to failed runs, and is taken into account in the cost calculation.

This approach was similarly applied for all methods listed below.

	Cost per sample (Euro)	*Step of analysis*	*% failure*
Qiagen DNAeasy Kit	€ 4.00	Sample processing	1
Tips	€ 0.25		
Eppendeorfs vials	€ 0.01		
Gloves	€ 0.01		
General Reagents	€ 0.01		
Tips 200ul	€ 0.37	Library preparation	5
Tips 100 ul	€ 0.36		
Tips 1000 ul	€ 0.01		
Nextera Xt index	€ 2.49		
Agencourt Ampure XP	€ 1.77		
Tips 20 ul	€ 0.37		
PCR-tube	€ 0.02		

Micro-Plate	€ 0.29		
Gloves	€ 0.01		
Deepwell plate	€ 0.25		
Microseal A	€ 0.48		
Microseal B	€ 0.08		
Nextera XT DNA SAMP Prep	€ 38.12		
MiSeq Reagent Kit V2 (2x250)	€ 113.07	Sequencing	1

Staff time per sample in minutes

The following provides the estimated staff time per sample spent on each step, separately for professionals and for technicians. The amount of 'hands-on staff time' is indicated, i.e. the amount of staff time actually used to perform an activity, including maintenance of equipment and staff time used for failed runs, but excluding unsupervised processes (e.g. time that the sequencer is running unsupervised). Where several samples are treated at the same time, total staff time is divided to obtain the per-sample staff time. For example, if sample processing for 40 samples takes 2 hours and 40 minutes for a laboratory technician, this figure is converted to minutes (160 minutes), and divided by 40, resulting in a technician staff time of 4 minutes per sample.

This approach was similarly applied for all methods listed below.

Staff category<br>*Step*	*Professionals**<br>*(staff time in minutes)*	*Technicians***<br>*(staff time in minutes)*
Sample processing	0	20
Library preparation	0	10
Sequencing	0	5
Bioinformatics & other analyses	60	0
Reference dataset	10	0

The definition of these categories is based on the International Standard Classification of Occupations of the International Labour Office (ILO).

**For "Professionals", occupations typically involve the performance of tasks that require complex problem-solving, decision-making and creativity based on an extensive body of theoretical and factual knowledge in a specialised field. The knowledge and skills required are typically obtained as the result of study at a higher educational institution for a period of 3-6 years following completion of secondary education leading to the award of a first degree or higher qualification. This category includes PhD candidates and Post-docs.*

***For "Technicians", occupations typically involve the performance of complex technical and practical tasks that require an extensive body of factual, technical and procedural knowledge in a specialised field. The knowledge and skills required are usually obtained as the result of study at a higher educational institution for a period of 1-3 years following completion of secondary education. This category includes laboratory assistants.*

II. Conventional method A: Serotyping

Equipment

No equipment other than basic laboratory equipment is used for serotyping, therefore there are no associated costs.

Consumables

	Cost per sample (Euro)	% failure*
Media	€ 2.29	0.1
Antisera	€ 4.84	
Plasticware and gloves	€ 0.62	

Staff time per sample in minutes		
	Professionals	*Technicians*
Staff time in minutes	3	38

III. Conventional method B: PFGE

Equipment			
	Total purchase price (Euro)	*Annual maintenance costs (Euro)*	*Predicted lifespan (years)*
Shacking waterbath	€ 3 000	€ 0	10
Biorad Mapper Apparatus	€ 21 000	€ 0	10
Image Acquisition apparatus	€ 12 000	€ 0	10
Bionumerics License	€ 11 170	€ 0	10

Consumables		
	Cost per sample (Euro)	*% failure**
Media	€ 0.17	3
Buffers	€ 12.31	
Restriction Enzymes	€ 1.06	
Plasticware and gloves	€ 0.46	

Staff time per sample in minutes		
	Professionals	*Technicians*
Staff time in minutes	2.5	38

IV. Conventional method C: PCR Verification

Equipment			
	Total purchase price (Euro)	*Annual maintenance costs (Euro)*	*Predicted lifespan (years)*
Biorad-T100 thermal cycler	€ 4 000	0	10
Image Acquisistioin apparatus	€ 12 000	0	10

Consumables		
	Cost per sample (Euro)	*% failure**

Media	€ 0.02	5
Baffers and reagents	€ 2.03	
Oligos and Taq	€ 0.36	
Plasticware and gloves	€ 0.24	
Staff time per sample in minutes		
	Professionals	*Technicians*
Staff time in minutes	1	10
**V. Conventional method D: MLVA**		
MLVA is outsourced to another lab in the institute's network, for a cost of € 43.13 per sample.		
**VI. Key variables**		
Labour costs		
The following table provides the hourly labour cost data (in Euro) used for monetisation of staff time. Figures below refer to Eurostat data on labour costs for 2017 (by staff category), plus a 25% surcharge for overheads.		
	Professionals	*Technicians*
Italy	€ 44.9	€ 23.9
EU	€ 45.1	€ 24.5
Source: Eurostat, Labour cost levels by NACE Rev. 2 activity [lc_lci_lev]. Construct: Labour cost for LCI (compensation of employees plus taxes minus subsidies). NACE categories: Professional, scientific and technical activities; Administrative and support service activities. Extracted in June 2018.		
Exchange rate (if relevant)		
…		
…		
Other		
…		
…		

ANNEX: Data collected for cost calculation - ANLIS

I. WGS

Equipment

In the following, the equipment used for sample preparation, sequencing, bioinformatics and other analyses considered for the cost calculation is listed. For each piece of equipment, the table provides the total unit price at the time of purchase (including VAT), annual maintenance costs, and predicted lifespan. Only equipment was considered that costed EUR 400 or more that qualify as capital expenditure relevant for WGS, such as sequencing machines and durable lab equipment as well as specific software purchasing or licensing fees. Not included were basic laboratory equipment (e.g. refrigerators, centrifuges or pipettes), standard office computers and standard office software. Note that the predicted lifespan of equipment is based on standard values and applied uniformly across case studies. Lifespans used for accounting purposes by each case institution may differ.

This approach was similarly applied for all methods listed below.

	Total purchase price (Euro)	*Annual maintenance costs (Euro)*	*Predicted lifespan (years)*
Qiacube DNA	€ 13 724	€ 974	10
Qubit 3.0	€ 1 743	€ 0	10
Bioshake iQ Thermomixer	€ 1 201	€ 0	10
MiSeq Illumina	€ 75 273	€ 6 072	10
Server	€ 19 474	€ 0	5
Computer	€ 3 614	€ 452	5
Computer	€ 3 614	€ 452	5

Consumables

In the following, the consumables used for sample preparation and sequencing considered for the cost calculation are listed. Consumables include items that are used up in laboratory processes, such as chemicals, petri dishes, etc. For each item, the table provides the cost per sample, the step of analysis it is used for and the failure rate. The failure rate refers to the percentage of consumables that are wasted, e.g. due to failed runs, and is taken into account in the cost calculation.

This approach was similarly applied for all methods listed below.

	Cost per sample (Euro)	*Step of analysis*	*% failure*
Qiacube box	€ 1.70	Sample processing	0
2mL Eppendorf DNA LoBindMicrocentifuge Tubes	€ 0.00		
Filter tips 200ul (1024) for Qiacube	€ 1.04		
Filter tips 1000ul (1024) for Qiacube	€ 0.70		
96 samples (Illumina, Cat # FC-131-1096)	€ 27.66	Library preparation	5
96 indices, 384 samples (Illumina, Cat # FC-131-1002)	€ 2.24		
Agencourt AMPure XP Beads, 60 ml (Beckman	€ 0.69		

Coulter, Cat # A63881)			
Qubit reagent BR	€ 0.46		
Qubit reagent HS	€ 0.46		
100 ul Filter tips	€ 0.11		
10 ul Filter tips	€ 0.11		
1000 ul filter tips	€ 0.07		
General consumables	€ 1.77		
MiSeq Reagent Kit v2 500 cycles	€ 62.79	Sequencing	5

Staff time per sample in minutes

The following provides the estimated staff time per sample spent on each step, separately for professionals and for technicians. The amount of 'hands-on staff time' is indicated, i.e. the amount of staff time actually used to perform an activity, including maintenance of equipment and staff time used for failed runs, but excluding unsupervised processes (e.g. time that the sequencer is running unsupervised). Where several samples are treated at the same time, total staff time is divided to obtain the per-sample staff time. For example, if sample processing for 40 samples takes 2 hours and 40 minutes for a laboratory technician, this figure is converted to minutes (160 minutes), and divided by 40, resulting in a technician staff time of 4 minutes per sample.

This approach was similarly applied for all methods listed below.

Staff category<br>*Step*	*Professionals**<br>*(staff time in minutes)*	*Technicians***<br>*(staff time in minutes)*
Sample processing	11	0
Library preparation	18	0
Sequencing	2	0
Bioinformatics & other analyses	60	0
Reference dataset	0	0

The definition of these categories is based on the International Standard Classification of Occupations of the International Labour Office (ILO).

**For "Professionals", occupations typically involve the performance of tasks that require complex problem-solving, decision-making and creativity based on an extensive body of theoretical and factual knowledge in a specialised field. The knowledge and skills required are typically obtained as the result of study at a higher educational institution for a period of 3-6 years following completion of secondary education leading to the award of a first degree or higher qualification. This category includes PhD candidates and Post-docs.*

***For "Technicians", occupations typically involve the performance of complex technical and practical tasks that require an extensive body of factual, technical and procedural knowledge in a specialised field. The knowledge and skills required are usually obtained as the result of study at a higher educational institution for a period of 1-3 years following completion of secondary education. This category includes laboratory assistants.*

II. Conventional method A: Biochemical testing

Equipment

No equipment other than basic laboratory equipment is used for biochemical testing, therefore there are no associated costs.

Consumables		
	Cost per sample (Euro)	*% failure**
General consumables	*Not available*	*Not available*

Staff time per sample in minutes		
	Professionals	*Technicians*
Staff time in minutes	2	13.8

III. Conventional method B: Serotyping

Equipment
No equipment other than basic laboratory equipment is used for biochemical testing, therefore there are no associated costs.

Consumables		
	Cost per sample (Euro)	*% failure**
General consumables	*Not available*	*Not available*

Staff time per sample in minutes		
	Professionals	*Technicians*
Staff time in minutes	10	35

IV. Conventional method C: PCR typing

Equipment			
	Total purchase price (Euro)	*Annual maintenance costs (Euro)*	*Predicted lifespan (years)*
Biorad Mycycler thermal cycler	€ 2 466	0	10

Consumables		
	Cost per sample (Euro)	*% failure**
General consumables	*Not available*	*Not available*

Staff time per sample in minutes		
	Professionals	*Technicians*
Staff time in minutes	20	0

V. Conventional method D: MaldiTOF

Equipment			
	Total purchase price (Euro)	*Annual maintenance costs (Euro)*	*Predicted lifespan (years)*

MaldiTOF	€ 188 239	0	10
Consumables			
	Cost per sample (Euro)	*% failure**	
General consumables	*Not available*	*Not available*	
Staff time per sample in minutes			
	Professionals	*Technicians*	
Staff time in minutes	10	0	
**VI. Conventional method E: PFGE**			
Equipment			
	Total purchase price (Euro)	*Annual maintenance costs (Euro)*	*Predicted lifespan (years)*
PFGE Biorad	€ 32 157	0	10
Consumables			
	Cost per sample (Euro)	*% failure**	
General consumables	*Not available*	*Not available*	
Staff time per sample in minutes			
	Professionals	*Technicians*	
Staff time in minutes	25	0	
**VII. Key variables**			
Labour costs			
The following table provides the hourly labour cost data (in Euro) used for monetisation of staff time. Figures below refer to data provided by ANLIS on labour costs for professional staff for 2017, plus a 25% surcharge for overheads. Labour costs for technician staff were imputed from professional staff costs.			
	Professionals	*Technicians*	
Argentina	€ 4.52	€ 2.67	

ANNEX : Data collected for cost calculation - MDH

I. WGS

Equipment

In the following, the equipment used for sample preparation, sequencing, bioinformatics and other analyses considered for the cost calculation is listed. For each piece of equipment, the table provides the total unit price at the time of purchase (including VAT), annual maintenance costs, and predicted lifespan. Only equipment was considered that costed EUR 400 or more that qualify as capital expenditure relevant for WGS, such as sequencing machines and durable lab equipment as well as specific software purchasing or licensing fees. Not included were basic laboratory equipment (e.g. refrigerators, centrifuges or pipettes), standard office computers and standard office software. Note that the predicted lifespan of equipment is based on standard values and applied uniformly across case studies. Lifespans used for accounting purposes by each case institution may differ.

This approach was similarly applied for all methods listed below.

	Total purchase price (Euro)	*Annual maintenance costs (Euro)*	*Predicted lifespan (years)*
MagNA Pure 24	€ 44 260	€ 8 062	10
Multichannel & Single Channel Pipettes	€ 3 203	€ 0	5
Illumina MiSeq	€ 84 093	€ 13 694	10
Illumina MiSeq	€ 71 531	€ 13 694	10
CLC Genomics WorkBench	€ 3 895	€ 974	10
BaseSpace annual iCredit subscription	€ 0	€ 1 328	1
PC	€ 1 770	€ 0	5

Consumables

In the following, the consumables used for sample preparation and sequencing considered for the cost calculation are listed. Consumables include items that are used up in laboratory processes, such as chemicals, petri dishes, etc. For each item, the table provides the cost per sample, the step of analysis it is used for and the failure rate. The failure rate refers to the percentage of consumables that are wasted, e.g. due to failed runs, and is taken into account in the cost calculation.

This approach was similarly applied for all methods listed below.

	Cost per sample (Euro)	*Step of analysis*	*% failure*
MagNA Pure 24 Processing Cartridge	€ 3.58	Sample processing	1
Magnapure 24 Total NA isolation kit	€ 5.60		
MagNA Pure Filter Tips 1000uL	€ 0.86		
MagNA Pure Tube (2mL)	€ 0.94		
Sealing Foil	€ 0.14		
MagNA Pure 24 Tip Park & Piercing tools	€ 0.24		
*Nextera XT Library Prep (v2 kit)**	€ 26.28	Library preparation	5.25

Nextera XT Library Prep (v3 kit)†	€ 26.28		
*Index Set A**	€ 2.12		
Index Set A†	€ 2.12		
*Index Set C**	€ 2.12		
Index Set C†	€ 2.12		
Ampure XP	€ 1.33		
Disposables (racks, pipette tips, gown, gloves, etc)	€ 10.62		*Factored into per-sample cost*
*500 Cycle v2 Kit**	€ 56.43	Sequencing	7.4
600 Cycle v3 Kit†	€ 40.19		6.6

*Note: MDH used both v2 (batch size of 16) and v3 (batch size of 32) library preparation and sequencing kits during the case study period, and indicated that these kits were used about equally. The per-sample costs for these consumables have been adjusted for their relative use and batch sizes. Items in the list above indicated with * belong to the v2 kit and † to the v3 kit.*

Staff time per sample in minutes

The following provides the estimated staff time per sample spent on each step, separately for professionals and for technicians. The amount of 'hands-on staff time' is indicated, i.e. the amount of staff time actually used to perform an activity, including maintenance of equipment and staff time used for failed runs, but excluding unsupervised processes (e.g. time that the sequencer is running unsupervised). Where several samples are treated at the same time, total staff time is divided to obtain the per-sample staff time. For example, if sample processing for 40 samples takes 2 hours and 40 minutes for a laboratory technician, this figure is converted to minutes (160 minutes), and divided by 40, resulting in a technician staff time of 4 minutes per sample.

This approach was similarly applied for all methods listed below.

Staff category<br>*Step*	*Professionals**<br>*(staff time in minutes)*	*Technicians***<br>*(staff time in minutes)*
Sample processing	1.87	0
Library preparation	12.19	0
Sequencing	0	0
Bioinformatics & other analyses	15.31	0
Reference dataset	0	0

The definition of these catgories is based on the International Standard Classification of Occupations of the International Labour Office (ILO).

**For "Professionals", occupations typically involve the performance of tasks that require complex problem-solving, decision-making and creativity based on an extensive body of theoretical and factual knowledge in a specialised field. The knowledge and skills required are typically obtained as the result of study at a higher educational institution for a period of 3-6 years following completion of secondary education leading to the award of a first degree or higher qualification. This category includes PhD candidates and Post-docs.*

***For "Technicians", occupations typically involve the performance of complex technical and practical tasks that require an extensive body of factual, technical and procedural knowledge in a specialised field.*

The knowledge and skills required are usually obtained as the result of study at a higher educational institution for a period of 1-3 years following completion of secondary education. This category includes laboratory assistants.

II. Conventional method A: PFGE

Equipment

	Total purchase price (Euro)	Annual maintenance costs (Euro)	Predicted lifespan (years)
GelDoc	€ 16 880	€ 531	10
GelDoc	€ 20 140	€ 531	10
GelDoc	€ 23 109	€ 531	10

Consumables

	Cost per sample (Euro)	% failure
Reagents	€ 13.72	2
Lab Supplies	€ 16.82	

Staff time per sample in minutes

	Professionals	Technicians
Staff time in minutes	58	0

III. Conventional method B: Real-Time PCR

Equipment

	Total purchase price (Euro)	Annual maintenance costs (Euro)	Predicted lifespan (years)
ABI 7500	€ 46 669	€ 7 967	10

Consumables

	Cost per sample (Euro)	% failure*
Reagents	€ 7.78	1
Lab Supplies	€ 4.65	

Staff time per sample in minutes

	Professionals	Technicians
Staff time in minutes	30	0

IV. Conventional method C: MALDI-TOF

Equipment

	Total purchase price	Annual maintenance	Predicted lifespan

	(Euro)	costs (Euro)	(years)
MALDI-TOF	€ 195 140	€ 17 704	10

Consumables		
	Cost per sample (Euro)	*% failure**
Reagents	€ 1.33	5
Lab Supplies	€ 1.77	

Staff time per sample in minutes		
	Professionals	*Technicians*
Staff time in minutes	2	0

**XIII. Key variables**		
Labour costs		
The following table provides the hourly labour cost data (in Euro) used for monetisation of staff time. Figures below refer to labour costs provided by the case study institution for country-specific costs and Eurostat data on labour costs for 2017 (by staff category) for EU costs. In both cases, a 25% surcharge has been added for overheads.		
	Professionals	*Technicians*
US	€ 42.05	*N/A*
EU	€ 45.10	€ 24.50
Source: US – data provided by MDH. EU - Eurostat, Labour cost levels by NACE Rev. 2 activity [lc_lci_lev]. Construct: Labour cost for LCI (compensation of employees plus taxes minus subsidies). NACE categories: Professional, scientific and technical activities; Administrative and support service activities. Extracted in June 2018.		

Other	
…	
…	

ANNEX : Data collected for cost calculation - PHAC

I. WGS

Equipment

In the following, the equipment used for sample preparation, sequencing, bioinformatics and other analyses considered for the cost calculation is listed. For each piece of equipment, the table provides the total unit price at the time of purchase (including VAT), annual maintenance costs, and predicted lifespan. Only equipment was considered that costed EUR 400 or more that qualify as capital expenditure relevant for WGS, such as sequencing machines and durable lab equipment as well as specific software purchasing or licensing fees. Not included were basic laboratory equipment (e.g. refrigerators, centrifuges or pipettes), standard office computers and standard office software. Note that the predicted lifespan of equipment is based on standard values and applied uniformly across case studies. Lifespans used for accounting purposes by each case institution may differ.

This approach was similarly applied for all methods listed below.

	Total purchase price (Euro)	*Annual maintenance costs (Euro)*	*Predicted lifespan (years)*
Tapestation	€ 38 883.96	€ 0.00	5
Blue Pippin	€ 10 232.62	€ 0.00	10
QUBIT	€ 2 524.05	€ 0.00	10
Illumina Miseq	€ 88 115.04	€ 9 216.90	10
Illumina Miseq	€ 88 115.04	€ 9 216.90	10
Illumina Miseq	€ 88 115.04	€ 9 216.90	10
Storage, NAS, 26 Nodes	€ 1 381 852.94	€ 0.00	5
Internal Networking	€ 142 691.34	€ 0.00	5
Compute Servers, 30 Nodes	€ 1 269 884.62	€ 0.00	5
BioNumerics Calculation Engine	€ 0.00	€ 10 923.74	10
BioNumerics Server	€ 17 054.37	€ 0.00	10
BioNumerics Client (7.x) x 10	€ 0.00	€ 12 289.21	1
BioNumerics Client (7.x) x 10	€ 75 039.23	€ 0.00	10
BioNumerics master scripts	€ 6 139.57	€ 0.00	10

Consumables

In the following, the consumables used for sample preparation and sequencing considered for the cost calculation are listed. Consumables include items that are used up in laboratory processes, such as chemicals, petri dishes, etc. For each item, the table provides the cost per sample, the step of analysis it is used for and the failure rate. The failure rate refers to the percentage of consumables that are wasted, e.g. due to failed runs, and is taken into account in the cost calculation.

This approach was similarly applied for all methods listed below.

	Cost per sample (Euro)	*Step of analysis*	*% failure*
EZ1 kit	€ 6.25	Sample processing	5
BioRad plates	€ 5.24		

Plates	€ 0.28	Library preparation	5
PCR CleanDX	€ 0.55		
TapeStation Tape + Reagent	€ 0.42		
TapeStation Tips	€ 0.04		
TapeStation 8-strip tubes	€ 0.02		
TapeStation plate	€ 0.00		
Reservoirs	€ 0.08		
Nextera XT Library Kit	€ 16.20		
2X KAPA HiFi HotStart ReadyMix	€ 3.73		
Pippin casette	€ 1.09		
Qubit (2x for each pool, reagent & tubes)	€ 0.21		
PCR CleanDX	€ 0.01		
Micrcon column	€ 0.29		
Cartridge + flow cell (600 v3)	€ 32.00	Sequencing	5

Staff time per sample in minutes

The following provides the estimated staff time per sample spent on each step, separately for professionals and for technicians. The amount of 'hands-on staff time' is indicated, i.e. the amount of staff time actually used to perform an activity, including maintenance of equipment and staff time used for failed runs, but excluding unsupervised processes (e.g. time that the sequencer is running unsupervised). Where several samples are treated at the same time, total staff time is divided to obtain the per-sample staff time. For example, if sample processing for 40 samples takes 2 hours and 40 minutes for a laboratory technician, this figure is converted to minutes (160 minutes), and divided by 40, resulting in a technician staff time of 4 minutes per sample.

This approach was similarly applied for all methods listed below.

Staff category / *Step*	*Professionals** (staff time in minutes)*	*Technicians*** (staff time in minutes)*
Sample processing	0	19.2†
Library preparation	0	
Sequencing	0	
Bioinformatics & other analyses	71.4	0
Reference dataset	19.0	0

† Figure provided for all wet-lab steps, including sample processing, library preparation and sequencing. Based on an average batch size of 32.

The definition of these categories is based on the International Standard Classification of Occupations of the International Labour Office (ILO).

For "Professionals", occupations typically involve the performance of tasks that require complex

problem-solving, decision-making and creativity based on an extensive body of theoretical and factual knowledge in a specialised field. The knowledge and skills required are typically obtained as the result of study at a higher educational institution for a period of 3-6 years following completion of secondary education leading to the award of a first degree or higher qualification. This category includes PhD candidates and Post-docs.
***For "Technicians", occupations typically involve the performance of complex technical and practical tasks that require an extensive body of factual, technical and procedural knowledge in a specialised field. The knowledge and skills required are usually obtained as the result of study at a higher educational institution for a period of 1-3 years following completion of secondary education. This category includes laboratory assistants.*

II. Conventional method A: Biochemical testing

Equipment

Basic laboratory equipment only (conventional test tubes)

Consumables

	Cost per sample (Euro)	*% failure*
General consumables	€ 2.30	5

Staff time per sample in minutes

	Professionals	*Technicians*
Staff time in minutes	0	40

III. Conventional method B: Serotyping

Equipment

Basic laboratory equipment only (conventional slide aggutination methods)

Consumables

	Cost per sample (Euro)	*% failure**
General consumables	€ 4.87	5

Staff time per sample in minutes

	Professionals	*Technicians*
Staff time in minutes	0	40

IV. Conventional method C: PFGE

Equipment

	Total purchase price (Euro)	*Annual maintenance costs (Euro)*	*Predicted lifespan (years)*
PFGE – CHEF DRIII	€ 37 855	€ 6 827	10
PFGE – CHEF DRIII	€ 37 855	€ 6 827	10
PFGE – CHEF DRIII	€ 37 855	€ 6 827	10

PFGE – CHEF DRIII	€ 37 855	€ 6 827	10
PFGE – CHEF DRIII	€ 37 855	€ 6 827	10
PFGE – CHEF DRIII	€ 37 855	€ 6 827	10
PFGE – CHEF DRIII	€ 37 855	€ 6 827	10
PFGE – CHEF DRIII	€ 37 855	€ 6 827	10
PFGE – CHEF DRIII	€ 37 855	€ 6 827	10
PFGE – CHEF DRIII	€ 37 855	€ 6 827	10

Consumables

	Cost per sample (Euro)	*% failure**
General consumables	€ 39.60	5

Staff time per sample in minutes

	Professionals	*Technicians*
Staff time in minutes	14.8	30.0

XIII. Key variables

Labour costs

The following table provides the hourly labour cost data (in Euro) used for monetisation of staff time. Figures below refer to labour costs provided by the case study institution for country-specific costs and Eurostat data on labour costs for 2017 (by staff category) for EU costs. In both cases, a 25% surcharge has been added for overheads.

	Professionals	*Technicians*
Canada	€ 41.03	€ 24.62
EU	€ 45.10	€ 24.50

Source: Canada – data provided by PHAC. EU - Eurostat, Labour cost levels by NACE Rev. 2 activity [lc_lci_lev]. Construct: Labour cost for LCI (compensation of employees plus taxes minus subsidies). NACE categories: Professional, scientific and technical activities; Administrative and support service activities. Extracted in June 2018.

Other

…	
…	

ANNEX : Data collected for cost calculation - PHE

I. WGS

Equipment

In the following, the equipment used for sample preparation, sequencing, bioinformatics and other analyses considered for the cost calculation is listed. For each piece of equipment, the table provides the total unit price at the time of purchase (including VAT), annual maintenance costs, and predicted lifespan. Only equipment was considered that costed EUR 400 or more that qualify as capital expenditure relevant for WGS, such as sequencing machines and durable lab equipment as well as specific software purchasing or licensing fees. Not included were basic laboratory equipment (e.g. refrigerators, centrifuges or pipettes), standard office computers and standard office software. Note that the predicted lifespan of equipment is based on standard values and applied uniformly across case studies. Lifespans used for accounting purposes by each case institution may differ.

This approach was similarly applied for all methods listed below.

	Total purchase price (Euro)	*Annual maintenance costs (Euro)*	*Predicted lifespan (years)*
QIASYMPHONY	€ 59 693	€ 17 681	10
QIASYMPHONY	€ 59 693	€ 17 681	10
ROCHE MAGNA PURE 96	€ 99 195	€ 6 844	10
cBot Cluster Generation System	€ 49 174	€ 4 563	10
cBot Cluster Generation System	€ 49 174	€ 4 563	10
LABCHIP GX	€ 52 950	€ 6 844	10
LABCHIP GX	€ 52 950	€ 6 844	10
ASSY-SCICLONE, G3 WGS, HV HEAD, L GRIP	€ 91 635	€ 10 266	10
ASSY-SCICLONE, G3 WGS, HV HEAD, L GRIP	€ 91 635	€ 10 266	10
ASSY-SCICLONE, G3 WGS, HV HEAD, L GRIP	€ 91 635	€ 10 266	10
LABCHIP-DS SPECTROPHOTOMETER 96	€ 48 584	€ 5 703	10
Glomax: 96 well plate Fluorometer	€ 14 749	€ 2 281	10
Glomax: 96 well plate Fluorometer	€ 14 749	€ 2 281	10
Biomek NXp Span-8 with integrated sealer and chilled storage	€ 160 770	€ 9 125	10
Biomex NXP Multichannel	€ 78 896	€ 8 745	10
Biomex NXP Multichannel	€ 78 896	€ 8 745	10
Biomex NXP Multichannel	€ 78 896	€ 8 745	10

Biomek NXP Span-8	€ 63 600	€ 9 125	10
Illumina HI-SEQ	€ 606 410	€ 57 034	10
Illumina HI-SEQ	€ 606 410	€ 57 034	10
Bioinformatics	Per-sample cost provided by PHE: € 4.89		

Consumables

In the following, the consumables used for sample preparation and sequencing considered for the cost calculation are listed. Consumables include items that are used up in laboratory processes, such as chemicals, petri dishes, etc. For each item, the table provides the cost per sample, the step of analysis it is used for and the failure rate. The failure rate refers to the percentage of consumables that are wasted, e.g. due to failed runs, and is taken into account in the cost calculation.

This approach was similarly applied for all methods listed below.

	Cost per sample (Euro)	*Step of analysis*	*% failure*
Various reagents and consumables	€ 6.84	Sample processing	0
96 indices, 384 samples	€ 1.94	Library preparation	0.1
nextera 96	€ 23.71		
PE Rapid cluster kit 2x96	€ 5.64		
cBot loading kit (rapid only) 2x 96	€ 1.84		
200 cycle rapid v2 2x96	€ 7.77	Sequencing	0.1
Other various costs	€ 1.88		

Staff time per sample in minutes

The following provides the estimated staff time per sample spent on each step, separately for professionals and for technicians. The amount of 'hands-on staff time' is indicated, i.e. the amount of staff time actually used to perform an activity, including maintenance of equipment and staff time used for failed runs, but excluding unsupervised processes (e.g. time that the sequencer is running unsupervised). Where several samples are treated at the same time, total staff time is divided to obtain the per-sample staff time. For example, if sample processing for 40 samples takes 2 hours and 40 minutes for a laboratory technician, this figure is converted to minutes (160 minutes), and divided by 40, resulting in a technician staff time of 4 minutes per sample.

This approach was similarly applied for all methods listed below.

Staff category / Step	*Professionals* (staff time in minutes)*	*Technicians** (staff time in minutes)*
Sample processing	2.65	16.85
Library preparation	1.60	0
Sequencing	2.60	0.30
Bioinformatics & other analyses	36.00	0
Reference dataset	0	0

The definition of these catgories is based on the International Standard Classification of Occupations of the International Labour Office (ILO).

**For "Professionals", occupations typically involve the performance of tasks that require complex problem-solving, decision-making and creativity based on an extensive body of theoretical and factual*

knowledge in a specialised field. The knowledge and skills required are typically obtained as the result of study at a higher educational institution for a period of 3-6 years following completion of secondary education leading to the award of a first degree or higher qualification. This category includes PhD candidates and Post-docs.
***For "Technicians", occupations typically involve the performance of complex technical and practical tasks that require an extensive body of factual, technical and procedural knowledge in a specialised field. The knowledge and skills required are usually obtained as the result of study at a higher educational institution for a period of 1-3 years following completion of secondary education. This category includes laboratory assistants.*

II. Conventional method A: PCR (Taqman)

Equipment

	Total purchase price (Euro)	*Annual maintenance costs (Euro)*	*Predicted lifespan (years)*
TaqMan 7500	€ 43 000	€ 1 141	10
TaqMan 7500	€ 43 000	€ 1 141	10
TaqMan 7500	€ 43 000	€ 1 141	10
TaqMan 7500	€ 43 000	€ 1 141	10
TaqMan 7500	€ 43 000	€ 1 141	10

Consumables

	Cost per sample (Euro)	*% failure**
Cupule	€ 0.08	*Costed into per-sample price*
Molecular water	€ 0.05	
Pipette tips	€ 0.07	
Plastic loops	€ 0.02	
Pre-aliquoted PCR strip HilA	€ 1.78	
Pre-aliquoted PCR strip lacZ+ttR	€ 0.14	

Staff time per sample in minutes

	Professionals	*Technicians*
Staff time in minutes	0	5.63

III. Conventional method B: PCR (Monophasic)

Equipment

	Total purchase price (Euro)	*Annual maintenance costs (Euro)*	*Predicted lifespan (years)*
TaqMan 7500	€ 43 000	€ 1 141	10
TaqMan 7500	€ 43 000	€ 1 141	10
TaqMan 7500	€ 43 000	€ 1 141	10
TaqMan 7500	€ 43 000	€ 1 141	10
TaqMan 7500	€ 43 000	€ 1 141	10

Consumables		
	Cost per sample (Euro)	*% failure**
Cupule	€ 0.08	*Costed into per-sample price*
Molecular water	€ 0.05	
Takyon PCR mastermix	€ 1.07	
fliC probe	€ 0.18	
fljB probe	€ 0.15	
fljB/IS200 probe	€ 0.14	
fliC_fw primer	€ 0.04	
fliC_rev primer	€ 0.05	
fljB_fw primer	€ 0.07	
fljB_rev primer	€ 0.05	
fliB/IS200_fw primer	€ 0.22	
fliB/IS200_rev primer	€ 0.05	
Fast 96 well PCR plate	€ 0.22	
Pipette tips	€ 0.05	
Plastic loops	€ 0.02	
Eppendorf tubes	€ 0.00	

Staff time per sample in minutes		
	Professionals	*Technicians*
Staff time in minutes	0	3.96

IV. Conventional method C: PCR (Real-Time)

Equipment			
	Total purchase price (Euro)	*Annual maintenance costs (Euro)*	*Predicted lifespan (years)*
Thermal cyclers	€ 2 446	€ 570	10
Thermal cyclers	€ 2 446	€ 570	10
Thermal cyclers	€ 2 446	€ 570	10
Thermal cyclers	€ 2 446	€ 570	10
Rotor gene	€ 30 831	€ 1 528	10
Rotor gene	€ 30 831	€ 1 528	10
Robot (Beckman etc.)	€ 61 662	€ 8 003	10

Consumables		
	Cost per sample (Euro)	*% failure**
Pipette tips filter	€ 0.77	*Costed into per-sample price*
Pastette fine tip	€ 0.06	

Pastette graduated	€ 0.03	
Universal Plastic 25ml	€ 0.56	
1.5ml skirted Microtube	€ 0.05	
gloves nitrile	€ 0.06	
Dispojar	€ 4.12	
Rotagene PCR strips	€ 0.09	
Probes	€ 1.39	
Primers	€ 1.39	
Water	€ 0.24	
Takyon	€ 0.74	
Staff time per sample in minutes		
	Professionals	*Technicians*
Staff time in minutes	2.50	3.00
**V. Conventional method D: MLVA/MLST/fAFLP**		
Equipment		
No equipment other than basic laboratory equipment is used for serotyping, therefore there are no associated costs.		
Consumables		
	Cost per sample (Euro)	*% failure**
Cupule	€ 0.08	*Costed into per-sample price*
Molecular water	€ 0.05	
Difco NA plates	€ 0.71	
MOLIS labels	€ 0.06	
Primers	€ 0.41	
Qiagen taq mix	€ 0.27	
2 ml tube	€ 0.04	
Nuclease free water (Severn)	€ 0.01	
filtered tips	€ 0.14	
microamp PCR plate	€ 0.36	
microamp PCR caps	€ 0.03	
Hi-Di	€ 0.04	
PCR plate Foil	€ 0.00	
Liz 1200	€ 0.62	
DBHT Frag. Analysis	€ 1.01	
Tips	€ 0.04	
Staff time per sample in minutes		

	Professionals	Technicians
Staff time in minutes	0	7.71

**VI. Conventional method E: Serotyping**			
Equipment			
	Total purchase price (Euro)	*Annual maintenance costs (Euro)*	*Predicted lifespan (years)*
Thermal cyclers	€ 2 466	€ 570	10
Thermal cyclers	€ 2 466	€ 570	10
Thermal cyclers	€ 2 466	€ 570	10
Thermal cyclers	€ 2 466	€ 570	10
Robot (Beckman etc.)	€ 61 662	€ 8,003	10

Consumables		
	Cost per sample (Euro)	*% failure**
MaConkey plates	€ 0.25	*Costed into per-sample price*
GIA	€ 0.75	
BHI (5ml,UV)	€ 1.86	
BHI (5ml,Tube)	€ 1.42	
Craigies	€ 1.19	
NA slopes (Tubes)	€ 0.71	
DE slopes	€ 0.68	
MOLIS labels	€ 0.02	
Microtitre plates	€ 0.43	
Serum (for'O' microtitre plates, 1:8)-2.7ml/plate	€ 2.89	
Serum (for'H' microtitre plates, 1:32)-2.7ml/plate	€ 0.88	
Serum (for craigies, 1:4)	€ 1.14	
Serum(for slide agglutination)	€ 0.19	
Serum (for titrations)	€ 0.06	
Formal saline	€ 0.41	
Phenol saline	€ 0.02	
Plastic loops	€ 0.11	
Plastic needles	€ 0.02	
Pastettes (short)	€ 0.17	
Gilson tips	€ 0.16	

Staff time per sample in minutes		
	Professionals	*Technicians*
Staff time in minutes	0	27.25

**VII. Conventional method F: Phage Typing**			
Equipment			
	Total purchase price (Euro)	*Annual maintenance costs (Euro)*	*Predicted lifespan (years)*
Thermal cyclers	€ 2 466	€ 570	10
Thermal cyclers	€ 2 466	€ 570	10
Thermal cyclers	€ 2 466	€ 570	10
Thermal cyclers	€ 2 466	€ 570	10

Consumables		
	Cost per sample (Euro)	*% failure**
Difco NA plates	€ 1.42	*Costed into per-sample price*
Dorsets egg slopes	€ 0.68	
Difco nutrient broth (double strength-4ml in tubes)	€ 0.59	
Pastettes	€ 0.07	
Plastic tips (for Pipetmax)	€ 0.14	
Phage suspension (0.16ml/NA plate)	€ 0.08	
Pipette tips	€ 0.05	
MOLIS labels (small)	€ 0.26	
MOLIS labels (V.small)	€ 0.19	

Staff time per sample in minutes		
	Professionals	*Technicians*
Staff time in minutes	2.25	12.5

VIII. Conventional method G: PFGE

No detailed cost data was available for PFGE. PHE's internal calculation of € 97.82 per sample was used instead as a unit cost.

IX. Conventional method H: D-Tartrate

Equipment

No equipment other than basic laboratory equipment is used for serotyping, therefore there are no associated costs.

Consumables		
	Cost per sample (Euro)	*% failure**
D-Tartrate tubes	€ 3.84	*Costed into per-sample price*

Plastic loops	€ 0.05	
Pastettes (short)	€ 0.03	
Lead acetate - saturated solution	€ 3.20	
MOLIS labels	€ 0.13	

Staff time per sample in minutes		
	Professionals	*Technicians*
Staff time in minutes	0	25.00

X. Conventional method I: Glucose gas

Equipment

No equipment other than basic laboratory equipment is used for serotyping, therefore there are no associated costs.

Consumables

	Cost per sample (Euro)	*% failure**
Glucose tube	€ 0.71	*Costed into per-sample price*
Plastic loop	€ 0.02	
MOLIS label	€ 0.06	

Staff time per sample in minutes		
	Professionals	*Technicians*
Staff time in minutes	0	10.00

XI. Conventional method J: AMR

Equipment

No equipment other than basic laboratory equipment is used for serotyping, therefore there are no associated costs.

Consumables

	Cost per sample (Euro)	*% failure**
Mackoney plates	€ 0.07	*Costed into per-sample price*
Saline in tubes	€ 0.07	
Microtitre plate	€ 0.01	
Plates	€ 0.58	
ISO agar + antibiotic	€ 0.33	
Muller hinton agar + antibiotic	€ 0.08	
Chromagenic agar	€ 0.01	
Loops	€ 0.04	

Tips	€ 0.04	
Labels	€ 0.14	
Eppendorf tubes	€ 0.04	

Staff time per sample in minutes		
	Professionals	*Technicians*
Staff time in minutes	0	2.00

XII. Conventional method K: Biochemistry

Equipment			
	Total purchase price (Euro)	*Annual maintenance costs (Euro)*	*Predicted lifespan (years)*
Thermal cyclers	€ 2 466	€ 570	10
Thermal cyclers	€ 2 466	€ 570	10
Thermal cyclers	€ 2 466	€ 570	10
Thermal cyclers	€ 2 466	€ 570	10
Biolog	€ 110 930	€ 11 115	10
Biolog	€ 110 930	€ 11 115	10

Consumables		
	Cost per sample (Euro)	*% failure**
Pipette tips filter	€ 1.54	*Costed into per-sample price*
Pastette fine tip	€ 0.13	
Pastette graduated	€ 0.07	
Universal Plastic 25ml	€ 0.56	
Gloves nitrile	€ 0.11	
Dispojar	€ 0.41	
Microgen plate	€ 9.73	
Inoculators	€ 1.16	
Reservoirs	€ 0.53	
Inoculating fluid	€ 0.44	
Other biochemistry media	€ 11.30	

Staff time per sample in minutes		
	Professionals	*Technicians*
Staff time in minutes	6.00	36.00

XIII. Key variables

Labour costs

The following table provides the hourly labour cost data (in Euro) used for monetisation of staff time.

Figures below refer to Eurostat data on labour costs for 2017 (by staff category), plus a 25% surcharge for overheads.		
	Professionals	*Technicians*
UK	€ 39.6	€ 25.0
EU	€ 45.1	€ 24.5
Source: Eurostat, Labour cost levels by NACE Rev. 2 activity [lc_lci_lev]. Construct: Labour cost for LCI (compensation of employees plus taxes minus subsidies). NACE categories: Professional, scientific and technical activities; Administrative and support service activities. Extracted in June 2018.		
Other		
…		
…		

1 Edejer T. Tan-Torres, R. Baltussen, T. Adam, R. Hutubessy, A. Acharya, D.B. Evans, and CJL. Murray, Making Choice in Health: WHO Guide to Cost-Effectiveness Analysis, 2003.

2 Buchanan, James, Sarah Wordsworth, and Anna Schuh, “Issues Surrounding the Health Economic Evaluation of Genomic Technologies”, Pharmacogenomics, Vol. 14, No. 15, 2013, Appendix 3: Costs which could be included in economic evaluations of genomic technologies. http://www.futuremedicine.com/doi/abs/10.2217/pgs.13.183.

3 Edejer T. Tan-Torres, R. Baltussen, T. Adam, R. Hutubessy, A. Acharya, D.B. Evans, and CJL. Murray, Making Choice in Health: WHO Guide to Cost-Effectiveness Analysis, 2003.

4 Civic Consulting (2016), Study on cost–benefit analysis of reference laboratories for human pathogens: final report, study conducted for CHAFEA of the European Commission and Civic Consulting (2009), Cost of National Prevention Systems for Animal Diseases and Zoonoses in Developing and Transition Countries, study conducted for the OIE.
